# Photoplethysmography upon cold stress—impact of measurement site and acquisition mode

**DOI:** 10.3389/fphys.2023.1127624

**Published:** 2023-06-01

**Authors:** Vincent Fleischhauer, Jan Bruhn, Stefan Rasche, Sebastian Zaunseder

**Affiliations:** ^1^ Laboratory for Advanced Measurements and Biomedical Data Analysis, Faculty of Information Technology, FH Dortmund, Dortmund, Germany; ^2^ Faculty of Medicine Carl Gustav Carus, TU Dresden, Dresden, Germany; ^3^ Professorship for Diagnostic Sensing, Faculty of Applied Computer Science, University Augsburg, Augsburg, Germany

**Keywords:** imaging photoplethysmography (iPPG), cold pressor test (CPT), pulse wave analysis (PWA), blood pressure, photoplethysmography (PPG)

## Abstract

Photoplethysmography (PPG) allows various statements about the physiological state. It supports multiple recording setups, i.e., application to various body sites and different acquisition modes, rendering the technique a versatile tool for various situations. Owing to anatomical, physiological and metrological factors, PPG signals differ with the actual setup. Research on such differences can deepen the understanding of prevailing physiological mechanisms and path the way towards improved or novel methods for PPG analysis. The presented work systematically investigates the impact of the cold pressor test (CPT), i.e., a painful stimulus, on the morphology of PPG signals considering different recording setups. Our investigation compares contact PPG recorded at the finger, contact PPG recorded at the earlobe and imaging PPG (iPPG), i.e., non-contact PPG, recorded at the face. The study bases on own experimental data from 39 healthy volunteers. We derived for each recording setup four common morphological PPG features from three intervals around CPT. For the same intervals, we derived blood pressure and heart rate as reference. To assess differences between the intervals, we used repeated measures ANOVA together with paired t-tests for each feature and we calculated Hedges’ *g* to quantify effect sizes. Our analyses show a distinct impact of CPT. As expected, blood pressure shows a highly significant and persistent increase. Independently of the recording setup, all PPG features show significant changes upon CPT as well. However, there are marked differences between recording setups. Effect sizes generally differ with the finger PPG showing the strongest response. Moreover, one feature (pulse width at half amplitude) shows an inverse behavior in finger PPG and head PPG (earlobe PPG and iPPG). In addition, iPPG features behave partially different from contact PPG features as they tend to return to baseline values while contact PPG features remain altered. Our findings underline the importance of recording setup and physiological as well as metrological differences that relate to the setups. The actual setup must be considered in order to properly interpret features and use PPG. The existence of differences between recording setups and a deepened knowledge on such differences might open up novel diagnostic methods in the future.

## 1 Introduction

Today, photoplethysmography (PPG) is an extremely popular metrological procedure. The technique supports multiple setups, i.e., it applies to various body sites and features different modes of application including finger clips, smart watches and non-contact approaches by cameras denoted as imaging photoplethysmography (iPPG). PPG signals and numerous features that can be derived from them carry wide information on the physiological state Almarshad et al. (2022); Elgendi (2012); Park et al. (2022). Recently, the usage of multiple PPG at a time, sometimes referred to as multisite PPG, has attracted attention as it even extends the possibilities for PPG based analyses Chan et al. (2019). There are, however, still limitations dealing with PPG. Such limitations relate to the origin of PPG signals, local peculiarities of signal acquisition, the interaction of multiple PPG signals, the behavior of features in dependency to influencing factors and features’ interpretation. Research on such aspects can deepen the understanding of prevailing physiological mechanisms, help to optimize metrological equipment and pave the way for improved or novel methods for PPG analysis.

This contribution is dedicated to a deeper characterization of PPG considering different measurement sites and acquisition modes. Our comparison includes contact PPG recorded at the finger, contact PPG recorded at the earlobe and iPPG, i.e., non-contact PPG, recorded at the face. Recordings at the finger are the traditional setup and most common way of application. Earlobe PPG is used less frequently but also well known as it can feature advantages, e.g., with respect to motion artifacts. iPPG is a relatively novel approach, which has become extremely popular over the last years. iPPG uses cameras to record the skin. The technique exploits subtle variations in the intensity of reflected light, which varies with blood filling of superficial vessels. Multiple current reviews provide good overviews on the fundamentals and applications of iPPG Molinaro et al. (2022); Selvaraju et al. (2022); Shao et al. (2021); Zaunseder and Rasche (2022). According to them, the vast majority of available works in the field of iPPG direct at heart rate and heart rate variability. However, there is a growing interest on morphological analyses and iPPG usage beyond heart rate.

Within this contribution, we employ PPG in the aforementioned three setups and focus on morphological features during a cold pressor test (CPT), i.e., a painful stimulus. CPT is a common tool in research and carries potential for diagnostics as well. We hypothesize that PPG derived features undergo changes upon CPT in all recording setups but we would expect differences between them. The presented research is worthwhile from two points of view. On the one hand, a more detailed understanding on the behavior of PPG derived features from single PPG signals and research on the interaction between different PPG is highly beneficial as it might contribute to refine existing analysis approaches or develop novel ones Natarajan et al. (2022). Particularly with respect to iPPG, ongoing debates regarding iPPG’s origin, a limited knowledge on influence factors and a reduced number of works dedicated to morphological analysis require basic research. On the other hand, further research on the CPT is beneficial as the physiological basics are not yet fully understood and standard values have to be established in order to develop strategies to integrate the CPT for diagnostic or prognostic purposes Lamotte et al. (2021).

The remainder of this work is structured as follows. Section 2 provides the background on the CPT and contains the results of a literature review concerning PPG usage during CPT. In section 3 we describe the used data, which originates from own multimodal experiments, the applied processing method and the statistics. Section 4 and section 5 provide results and discuss them.

## 2 Background on PPG during cold stress

### 2.1 Cold pressor test

Cold is known to elicit multiple physiological reactions. The CPT, i.e., the defined application of a cold stimulus, was firstly described for research purposes by Hines and Brown (1936); Lamotte et al. (2021). Since then, the CPT has become a widely used tool to study the cardiovascular system and autonomous nervous system, most often in terms of blood pressure as well as heart rate and its regulation Bali and Jaggi (2015); Mitchell et al. (2004); Mourot et al. (2009). The most common experimental CPT design requires immersing the hand into cold water. According to published works, water temperatures vary between 0°C and 7°C (even higher temperatures up to 20°C have been considered but then the perception is classified as cold sensation rather than as pain). Duration of immersion also varies, typically between 1 and 6 min while termination upon participant’s request is always possible. Immersion generates cold pain triggering sympathetic activation and parasympathetic withdrawal. Sympathetic activation causes a pronounced peripheral vasoconstriction. Positive inotropic and chronotropic effects due to both, sympathetic activation and parasympathetic withdrawal, accompany the peripheral vasoconstriction. Consequently, CPT increases blood pressure and heart rate immediately after immersion. However, heart rate was shown to be affected to a lesser extent or even to decrease again shortly after a first increase. A potential explanation is the baroreceptor reflex. Increased blood pressure triggers the baroreceptors leading to parasympathetic activation and a reduction of heart rate.

As a result, CPT has been consistently shown to yield a rather persistent blood pressure increase during CPT execution. Heart rate, in turn, shows a more indifferent and individual pattern during CPT Bali and Jaggi (2015); Mitchell et al. (2004); Mourot et al. (2009).

### 2.2 PPG during CPT

As stated before, the CPT is a widely used experimental technique. Many works explored and summarized the effect of CPT on blood pressure and heart rate. PPG during CPT is common but most often it serves to capture heart rate and its variability. The morphological analysis of the photoplethysmographic waveform during CPT is less common. To reflect the current state of knowledge on CPT’s effect on the photoplethysmographic waveform, we conducted a systematic literature review using pubmed. The review was done according to the Preferred Reporting Items for Systematic Reviews and Meta-Analyses (PRISMA). Our search considered articles that had the word “cold” and either the word “PPG”, “photoplethysmogra*“, “oximet*” or “oxymet*” in their title or abstract. We considered original articles only (reviews were excluded). Further inclusion criteria were the use of finger and/or earlobe PPG and/or iPPG and the examination of features related to the shape of PPG signals (not only heart rate and heart rate variability). In addition, inclusion required that the cold stimulus of the CPT was not applied at the measurement site (cooling the measurement site introduces additional effects beside the effect of painful stimulus). The search was limited to studies that were conducted on or regarding human subjects. Additional exclusion criteria were the unavailability of full texts or articles written in languages other than English or German.

**FIGURE 1:**
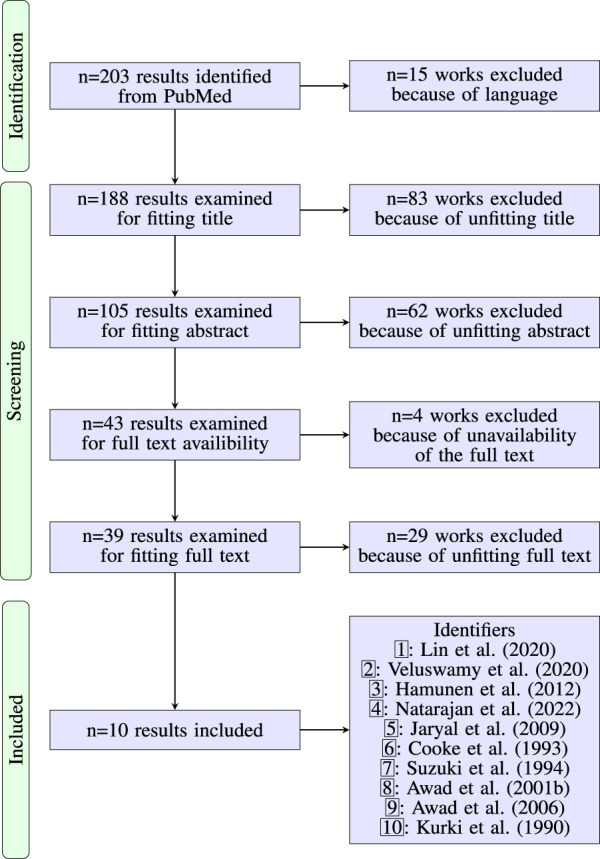
PRISMA flow diagram on the literature research regarding the effect of CPT on the waveform of the PPG. Table 1 contains details for the included works.

Figure 1 shows the PRISMA flow chart. The initial search resulted in 203 articles. 15 articles were excluded for non-fitting language. 188 works were then further reviewed to exclude works that we deemed unfitting based on titles, abstracts or full texts. After screening the results for unfitting titles we excluded 83 articles. Of the remaining 105 articles we excluded another 62 based on their abstracts. Four additional articles were excluded for missing/non-existent full texts. After review of the full texts of the remaining 39 articles, we excluded 29 of them and finally ended up with ten articles that matched our inclusion criteria. Common reasons to exclude articles during review were, for example, the lack of a CPT, cooling at the measurement site and lacking usage of the PPG waveform.

Table 1 summarizes details on the studies that were finally included. The considered works invoked seven to 33 subjects. Healthy subjects were always considered. Five works also included patients. The way cold stress was induced varied between studies. Besides immersion of the hand into water Awad et al. (2001b), Awad et al. (2006); Hamunen et al. (2012); Jaryal et al. (2009); Cooke et al. (1993); Kurki et al. (1990), finger immersion Suzuki et al. (1994), foot immersion Natarajan et al. (2022) and holding an ice bottle in the hand Lin et al. (2020) were described. Used temperatures to yield a cold response varied in a wide range up to 20°C Cooke et al. (1993). Some works also employed multiple temperatures to investigate the effect of the actual temperature Suzuki et al. (1994); Veluswamy et al. (2020). As PPG measurement site, fingers were most common. Regarding the used features, by far the most often used feature was the amplitude. Besides, features related to the slope and rise time are common. Other features, like areas and derivative features, have also been considered (particularly as some researchers invoked large feature sets Lin et al. (2020); Natarajan et al. (2022)) but are not that common.

The studies rather consistently describe a significant effect of CPT on the amplitude with very few exceptions (concerning the earlobe in PPG Awad et al. (2001b) and the finger in Kurki et al. (1990)). Most other features also show significant changes upon CPT. Changes exist (almost) independently of the actual way of stimulation though effect sizes increase with decreasing stimulus temperature. Only few works directly compare PPG from different sites. Awad et al. (2001b), Awad et al. (2006) describe a much more pronounced response within the finger PPG compared to earlobe PPG visible in multiple features including amplitude and area. Natarajan et al. (2022) make use of PPG features to estimate blood pressure. Dependent on the measurement site, different features can make a valuable contribution. Such a finding also hints at a non-uniform local behavior. With respect to pathology, available works indicate changes Kurki et al. (1990); Veluswamy et al. (2020); Jaryal et al. (2009); Cooke et al. (1993). There is, however, no uniform behavior but the effects caused by CPT can be less or more pronounced according to the specific disease.

**TABLE 1:** Overview on the included works according to Figure 1 (IDs are given there). Column “population” provides details on the cohorts. Age is given in years as provided by the original references. Column “features” contains those features that originate from PPG and carry morphological information. Column “main findings” concern findings directly related to CPT. ^†^ “Indices” denote features that are combinations of amplitude and pulse interval. ^‡^ The study features two parts and invokes patients. As CPT only applies to healthy controls, we restrict the provided information to this part. Note that the population is probably the same as in 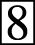
but other features were considered. RSD - reflex sympathetic dystrophy, RSI - repetitive strain injury, PWHA - pulse width at half amplitude.

Id	Population	Site(s)	Feature(s)	Main finding(s)
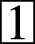	12 (2 f/10 m), healthy, 27.8 ± 5.4 (mean ± std)	finger	amplitudes, slopes, areas, intra-beat time intervals (65 features)	significant changes in almost all features but intra-beat time intervals
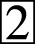	33 (13 f/20 m), sickle cell disease (17)/healthy controls (16), age ≥13	finger	amplitude	significant reduction in amplitude; effect more pronounced (faster, stronger) in sickle cell disease
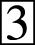	29 (0 f/29 m), age 24 (18–28) (mean (range)), healthy	finger	amplitude, PPG derived indices^†^ (4 features)	significant changes in all features
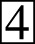	32 (16 f/16 m), age 52 ± 17 (mean ± std), healthy (24)/hypertensive (8)	finger, earlobe, toe	amplitudes, derivative features, areas, time intervals (31 features)	parameters related to fast upstroke provide added value for blood pressure estimation
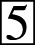	21 (9 f/12 m), diabetes mellitus (10)/healthy controls (11), age 47 ± 8 (mean ± std)	finger	amplitude, slope, crest time, decay time	amplitude and slope affected most by CPT (even after termination); reduced effects in patients
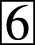	27 (18 f/9 m), RSD (6)/RSI (9)/healthy controls (12)/age 39.0/39.8/30.1 ± 11.7/13.6/8.6 (mean ± std per group)	finger	amplitude	amplitude change in disease less pronounced
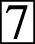	7 (0 f/7 m), healthy, age: 23–29 (range)	finger	amplitude	significant amplitude reduction; strength related to water temperature
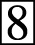	12 (0 f/12 m), healthy, age: 25–50 (range)	finger, earlobe	amplitude	70% reduction in finger amplitude (significant) and 10% reduction in earlobe amplitude (non-significant)
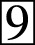	12 (0 f/12 m), healthy, age: 25–50 (range)	finger, earlobe	amplitude, area, upstroke slope, downstroke slope, PWHA	significant decrease in all finger PPG features; only significant change in earlobe PPG increase of PWHA
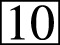	30 (28 f/2 m), Raynaud’s phenomenon (15)/healthy controls (15), age 38.4(25–54)/55.0(38–71) (mean (range) per group)	finger	amplitude	decrease (non-significant) in controls and patients

## 3 Materials and methods

### 3.1 Data

**Overview:** The used data originates from own multimodal experiments invoking healthy volunteers of Caucasian origin. The whole experimental protocol contained different stimuli, namely, paced deep breathing (PDB), multiple orthostatic maneuvers and CPT. Throughout the experiment, we recorded multiple vital signs and videos. Below we detail the experimental procedure and the technical equipment. All subjects gave written consent. The study was approved by the Ethics Committee at TU Dresden (EK 311082018).

**FIGURE 2:**
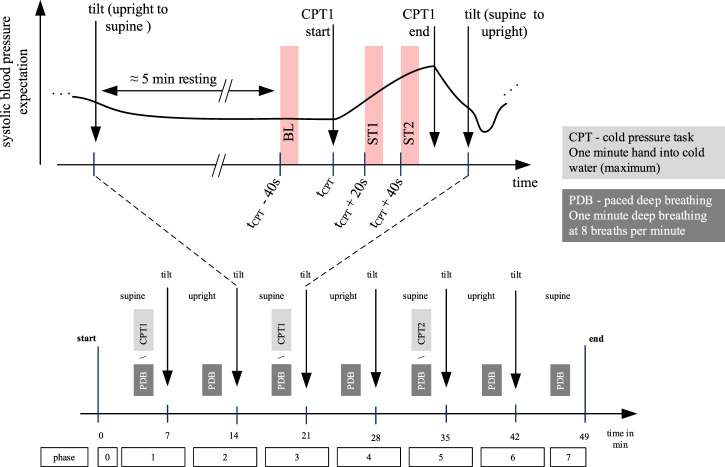
Overview on the whole experimental protocol (lower part) and CPT1 (upper part). This work uses data from CPT1 only, which was randomized executed in phase one or phase three. For each subject we define three time windows of 10 s: baseline (BL), stimulation 1 (ST1) and stimulation 2 (ST2). Independently of the CPT execution in phase one or phase three, there was a resting epoch for approximately 5 minutes before the CPT.

**Procedure:** Figure 2 provides an overview on the whole experimental protocol and the part of it that was considered in this contribution. The experiment lasted approximately 49 min. During execution, the tilt-table was alternated between supine and upright position every 7 min defining seven phases. Between orthostatic maneuvers participants had resting epochs and executed CPT or PDB. Each participant executed at least one CPT (denoted as CPT1), which was randomly assigned to phase one or phase three. A random subset of participants executed another CPT (CPT2) in phase five. The presented investigation uses data from CPT1 (i.e., CPT2, tilting and PDB are not relevant for this contribution). CPT required participants to put their left hand into cold water (temperature was approximately 4°C, which should yield a strong effect according to the literature Suzuki et al. (1994); Veluswamy et al. (2020)). Immersion was intended for 60 s but participants could terminate before that time if they felt (too) uncomfortable. During immersion, participants stayed in supine position and tried to keep the position of their face and right arm as constant as possible. For the analysis, we defined the following three time intervals of 10 s in relation to the time of immersion *t*_CPT_: baseline (BL), starting at *t*_CPT_ − 30 s, stimulation 1 (ST1), starting at *t*_CPT_ + 20 s and stimulation 2 (ST2), starting at *t*_CPT_ + 40 s. According to the preceding interval at rest, we assume BL to represent a stable state, which is interchangeable for phase one and phase three. For ST1 and ST2 we expect a notable effect of the pain stimulus. The time interval ST1 was chosen as we assumed the painful stimulus already having caused an effect. ST2 represents the latest possible interval to see if different recording setups diverge over the experiment. As subjects were told shortly before the end of CPT to be prepared for removing their hands, we avoided to use the last 10 s of the record. Note that we do not expect the reaction upon cold stress to be terminated at ST2. However, our investigation does not aim to explore the full temporal behavior upon cold stress but explores the immediate effect of a painful stimulus considering different measurement sites and acquisition modes. According to Hamunen et al. (2012) there seems to be a “saturation of unpleasantness” after approximately 60 s, which renders 60 s a suitable duration. 

**FIGURE 3:**
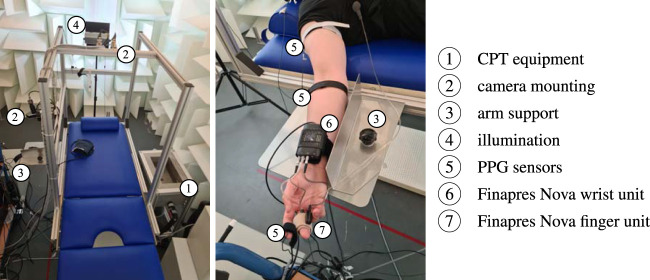
Illustration of the used equipment.

**Equipment:** The used equipment is depicted in Figure 3. Vital signs and RGB videos were continuously recorded throughout the whole experiment. We used two biosignal amplifiers Biopac MP36 (Biopac; Goleta, United States of America) and reflective photoplethysmographic signal transducer SS4LA (Biopac; Goleta, United States of America) to record (contact) PPG signals from right earlobe and right index finger at a sampling rate of 2000 Hz with an emitter/detector wavelength of 860 ± 60 nm (we also recorded PPG at right antecubital fossa and right shoulder area but they are not relevant to this contribution). In addition, we used the Finapres Nova (Finapres Medical Systems; Enschede, Netherlands) to record continuous non-invasive blood pressure and a single lead electrocardiogram (Einthoven II). Videos were recorded by three UI-3060CP-C-HR Rev 2 RGB cameras (IDS Imaging Development Systems GmbH; Obersulm, Germany). The cameras were mounted on the tilt table with fixed orientation regarding the subject during the experiment. For this work, solely camera 2 is of particular interest. This camera recorded the subject’s head at a distance of approximately 40 cm. The recorded area covered the head and a small portion of the shoulders. Videos were captured at a color depth of 12 bit, a frame rate of 25 Hz and a spatial resolution of 1,280 × 960 pixel. All videos were stored in a proprietary format with lossless compression. The recordings took place in a controlled environment using indirect artificial illumination by two spotlights Walimex pro LED Sirius 160 Daylight 65 W (color temperature 5,600 K, color rendering index ≥90 ) (WALSER GmbH *&* Co. KG; Gersthofen, Germany). Inputs from cameras and Finapres were fed into the Biopac MP36 biosignal amplifiers for synchronization of modalities.

**Used data:** Overall, 61 recordings were carried out using the setting described above. We excluded 18 recordings which showed intermittent technical problems (early recordings partially suffered from a software issue, which could lead to missing data in at least one video or signal; we completely discarded such recordings for this analysis). We further excluded four recordings, which were stopped upon participant’s request owing to malaise from tilting. Overall, we include 39 recordings in our analysis (13 female, 26 male; age: 30.5 ± 12.0 years; body height: 177 ± 7.83 cm; body weight: 76.5 ± 14.9 kg). As stated before, from each recording we only consider CPT1.

### 3.2 PPG processing

In general, we applied the same approach to process PPG and iPPG signals. However, iPPG processing first requires signal formation including region of interest (ROI) segmentation, ROI tracking and signal extraction. The further processing of all signals - contact and non-contact PPG signals - included filtering, beat detection, template construction and feature extraction by means of pulse wave decomposition (PWD) and using derivatives of the respective signals.

**FIGURE 4:**
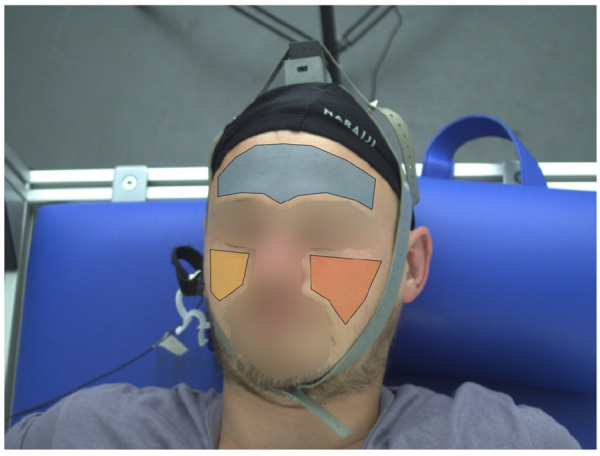
Exemplary ROI definition. Shown are three facial ROIs (forehead (blue polygon), right cheek (yellow polygon), left cheek (orange polygon)). To derive an iPPG signal we use the forehead ROI alone and all ROIs in combination as a “super” ROI.

**iPPG signal formation:** To acquire iPPG signals from our videos, we manually defined polygons for the forehead as our ROIs in the first frame of the first interval (BL). We also defined ROIs for both cheeks and used the combination of all face ROIs as a “super” ROI to mimic common ROIs generated by automatic segmentation. For each of the following intervals ST1 and ST2, we shifted the ROIs to fit slightly changed body positions. The ROIs remained static for the duration of each interval. We spatially smoothed the videos with an averaging filter of 10 pixel width and then obtained iPPG signals by averaging all pixels inside the ROI. The signals were then inverted to resemble the conventional PPG and linear interpolated to a sampling rate of 2000 Hz in order to match the sampling rate of contact PPG. Figure 4 shows an example of the defined ROIs.

**FIGURE 5:**
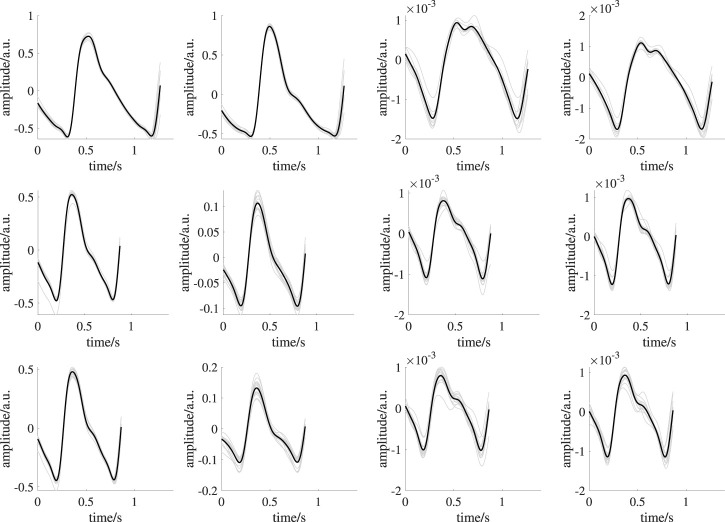
Exemplary template generation of one subject. From left to right: earlobe PPG, finger PPG, forehead iPPG, super ROI iPPG. The upper row shows signals during BL. The middle row shows signals during ST1. The lower row shows signals during ST2. Black lines indicate mean beat templates; gray lines indicate the corresponding beat segments.

**Signal processing:** We filtered the PPG and iPPG signals with a bandpass filter (fifth-order Butterworth filter with cut-off frequencies of 0.4 Hz and 8 Hz). Single beats from the PPG signals were detected with the method of Làzaro et al. (2014). The method considers the steepest ascent as the detection point *t*_*i*_. Around each detection *t*_*i*_ we defined a beat segment in the interval $\left[t_{i}-0.45\cdot \tilde{\text{BBI}};t_{i}+\tilde{\text{BBI}}\right]$ , where $\tilde{\text{BBI}}$ is the median length of beat-to-beat intervals (BBI) within the considered interval. All detected beat segments were correlated pairwise. We discarded beat segments with a mean pairwise correlation lower than 0.3. The remaining segments were ensemble averaged and potential linear trends were removed to form a beat template. Figure 5 shows exemplary template generations. The median number of usable beats for template generation per measurement site was 10.

**FIGURE 6:**
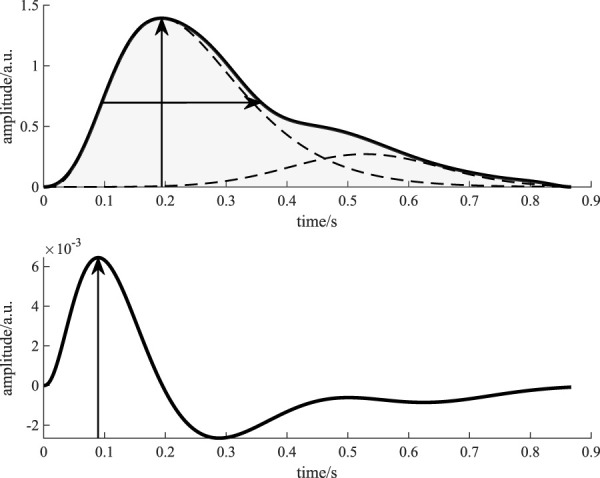
Visualization of the features derived from a beat template. The upper figure shows the beat template (solid black line) and the kernels (dashed black lines). The vertical arrow indicates the feature amplitude, while the horizontal arrow shows the feature PWHA. The light gray area marks the feature area. The lower figure shows the first derivative of the recomposed template beat (solid black line). The vertical arrow indicates the maximum of the first derivative, i.e., the feature slope.

**Feature extraction:** We applied pulse wave decomposition and recomposition to each template for denoising. We used the GammaGaussian2 decomposition algorithm (i.e., decomposition by a Gamma kernel and a Gaussian kernel, see Figure 6) that was described previously Fleischhauer et al. (2020). A reconstructed beat *y* for the Gamma-Gaussian algorithm with 2 kernels can be described as:

$$y_{\text{GammaGaussian}2}\left(t,\theta \right)=\frac{\beta_{1}^{\alpha_{1}}}{s_{1}\cdot \Gamma \left(\alpha_{1}\right)}t^{\alpha_{1}-1}e^{-\beta_{1}t}+a_{2}\cdot e^{\left(\frac{-{\left(t-\mu_{2}\right)}^{2}}{2\sigma_{2}^{2}}\right)}.$$ (1)

Each reconstructed beat is a function of time *t* and an optimization vector *θ* = [*a*, *μ*, *σ*]. The interior point optimization algorithm fits the kernels to the template beat using the constraints *a*_1_ > *a*_2_, *μ*_1_ < *μ*_2_, i.e., the Gamma kernel has to occur before the Gaussian kernel and its amplitude has to be higher. The initial values for the algorithm are explained in detail in Fleischhauer et al. (2020). Figure 6 displays the processing of the template beats. Considering the literature on PPG during CPT and common procedure in PPG processing, we selected four features for analysis: amplitude (maximum of the template), slope (maximum of the first derivative of the template), area (area under the template) and PWHA (pulse width at half amplitude). Figure 6 illustrates the definition of these features.

### 3.3 Reference parameters

In addition to the PPG features under test, we considered blood pressure and heart rate as reference to verify if CPT yielded the expected effect. We used systolic blood pressure (SBP), diastolic blood pressure (DBP) and heart rate by Finapres Nova. The device yields values for such features for each single beat. From SBP and DBP we additionally derived pulse pressure (PP). For each interval (BL, ST1, ST2) we calculated one single value for each reference parameter by taking the median value of single beats’ values in the respective interval.

### 3.4 Statistical assessment

To evaluate the effect of CPT, we firstly conducted repeated measures ANOVA for each recording setup and feature on a significance level of *α* = 0.05 with no grouping of the subjects. For significant ANOVA results, we conducted paired t-tests as *post hoc* tests for all combinations of intervals (BL vs ST1, BL vs. ST2 and ST1 vs ST2). We tested each of these intervals against each other, thereby creating non-orthogonal contrasts. Thus, we used the Holm-Bonferroni correction to adjust the *p* values of our *post hoc* tests with the respective correction factor (*k* − *i* + 1) (with *k* being the number of conducted tests and *i* the rank of the *p* values sorted in ascending order) Holm (1979). As a measure of effect size we calculated Hedges’ *g* Hedges (1981).

$$g=J\left(\text{df}\right)\cdot \frac{\bar{x}-\bar{y}}{s}$$ (2)

$$s=\sqrt{\frac{\left(n_{x}-1\right)s_{x}^{2}+\left(n_{y}-1\right)s_{y}^{2}}{n_{x}+n_{y}-2}}$$ (3)

$$J\left(\text{df}\right)=\frac{\Gamma \left(\text{df}/2\right)}{\sqrt{\text{df}/2}\Gamma \left(\left(\text{df}-1\right)/2\right)}$$ (4)

$$\text{df}=n_{x}+n_{y}-2$$ (5)

Hedges *g* is a modification of Cohens *d*. It is defined as the difference of the means ( $\bar{x}$ and $\bar{y}$ ) of two groups of sizes *n*_*x*_ and *n*_*y*_ divided by their pooled empirical standard deviation *s*. The biased estimator is corrected by the factor *J* (df) that depends on the degrees of freedom df. Effect sizes *g* < 0.5 are considered small, while *g* > 0.8 is considered large Cohen (1988). For visualization purposes, we normalized all response variables, i.e., the PPG features, to the mean of the three intervals for the respective response variable. The same statistical procedure applies to the reference parameters SBP, DPB, PP and heart rate but we omitted normalization as absolute numbers are relevant there.

## 4 Results

**FIGURE 9:**
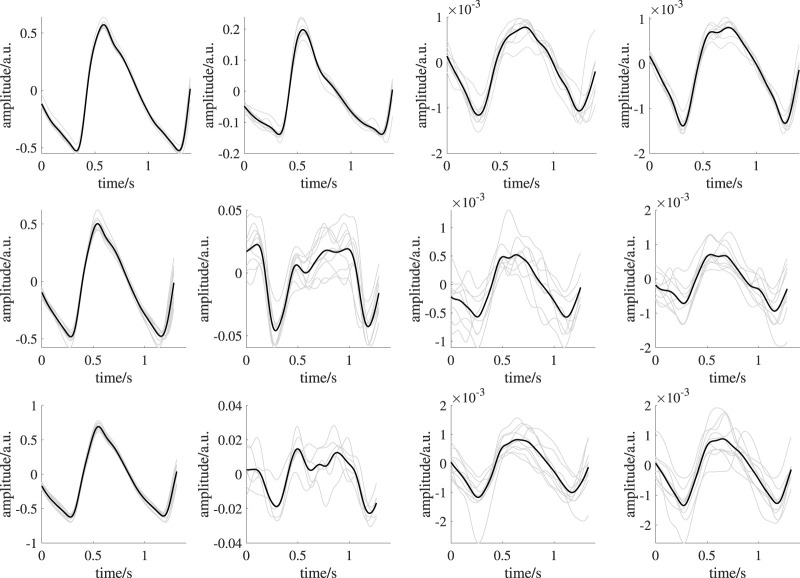
Exemplary template generation of one subject showing reduced quality templates. From left to right: earlobe PPG, finger PPG, forehead iPPG, super ROI iPPG. The upper row shows signals during BL. The middle row shows signals during ST1. The lower row shows signals during ST2. Black lines indicate mean beat templates; gray lines indicate the corresponding beat segments. The overall mean pairwise correlation of the beat segments forming the template for the finger PPG, forehead iPPG and super iPPG during ST1 and ST2 is reduced compared to those in Figure 5. Reduced quality templates lack distinct features (e.g., completely missing dicrotic notch in forehead iPPG during ST2, overall mean pairwise correlation: 0.71; corresponding overall mean pairwise correlation in Figure 5: 0.96) or exhibit unexpected morphologies (e.g., finger PPG during ST2, overall mean pairwise correlation: 0.64; corresponding overall mean pairwise correlation in Figure 5: 0.99) due to low correlation of the beat segments forming a template.

Figure 5 shows templates of one subject from all recording setups and time intervals as example. An overview on templates of all subjects is provided in the Supplementary Material. PPG quality should be mentioned here. While iPPG is known for limited signal quality, contact PPG was expected to be of high quality. However, in some cases, even contact PPG showed unstable beat shapes and distortions (e.g., see Figure 9). We excluded three subjects as no beat template could be generated in at least one of the analysis intervals. Another subject was excluded because of missing reference data. Those subjects were completely excluded from all analyses. Accordingly, the following results and statistical assessment base on data of 35 subjects.

**FIGURE 7:**
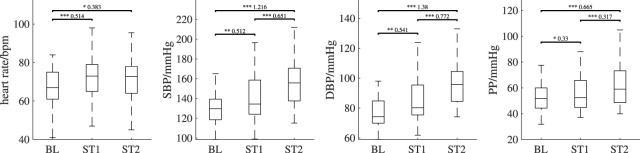
Behavior of the reference features (heart rate, systolic blood pressure (SBP), diastolic blood pressure (DBP) and pulse pressure (PP)) over all analysis intervals. If significant, *post hoc* tests’ outcome is denoted by * (*p* <0.05), ** (*p* <0.01) or *** (*p* <0.001). The numbers above the lines indicate effect sizes and are provided for significant *post hoc* tests only. Outliers are not shown; the numbers of outliers are provided in Table 2.

Figure 7 depicts the behavior of reference parameters (SBP, DBP, PP, heart rate) for all analysis intervals. For better visibility, we omitted outliers in those plots (values are defined as outliers if they are greater than *q*_3_ + 1.5 ⋅IQR or less than *q*_1_ − 1.5 ⋅IQR, where *q*_1_ is the first quartile, *q*_3_ is the third quartile and IQR is the interquartile range). Table 2 shows the corresponding number of outliers. Repeated measures ANOVA yielded outcomes of *p* < 0.001 for all reference features. SBP and DBP, both exhibit highly significant (*p* < 0.001) differences in pairwise comparisons of analysis intervals. PP shows a highly significantly increase in ST2 compared to BL and ST1 (*p* < 0.001). There is also a statistically significant increase from BL to ST1 (*p* < 0.05). Heart rate increases significantly between BL and ST1 (*p* < 0.001) and BL and ST2 (*p* < 0.01).

**TABLE 2:** Number of outliers not shown in boxplots for reference parameters and PPG features.

Feature	Measurement	BL	ST1	ST2
heart rate	*References*	1	2	3
SBP	*taken from*	1	1	1
DBP	*Finapres*	1	1	1
PP	*Nova*	1	1	1
amplitude	PPG finger	1	1	1
amplitude	PPG earlobe	2	2	1
amplitude	iPPG forehead	2	1	2
amplitude	iPPG super	2	1	1
slope	PPG finger	1	1	1
slope	PPG earlobe	2	2	1
slope	iPPG forehead	2	1	3
slope	iPPG super	3	1	2
area	PPG finger	1	1	1
area	PPG earlobe	1	1	1
area	iPPG forehead	1	1	1
area	iPPG super	2	1	1
PWHA	PPG finger	1	1	1
PWHA	PPG earlobe	2	5	1
PWHA	iPPG forehead	2	1	2
PWHA	iPPG super	1	1	1

**FIGURE 8:**
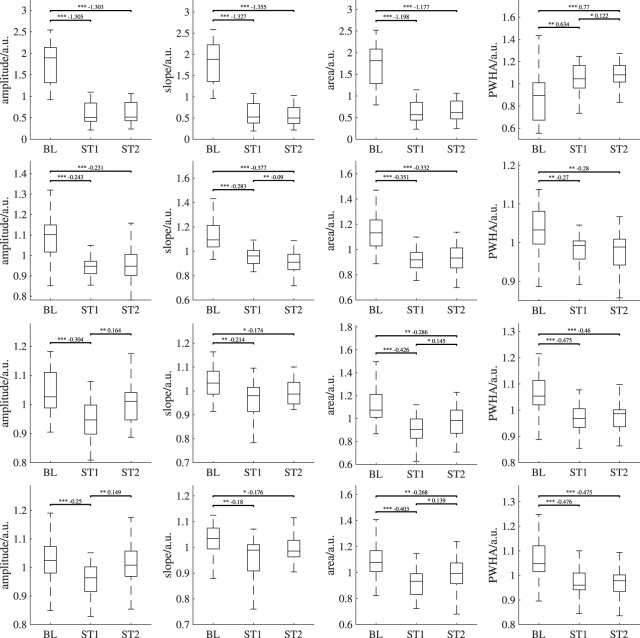
Behavior of the features derived from PPG and iPPG (amplitude, slope, area, PWHA) over all analysis intervals. First row: PPG (finger), second row: PPG (earlobe), third row: iPPG (forehead), fourth row: iPPG (super). If significant, *post hoc* tests’ outcome is denoted by * (*p* <0.05), ** (*p* <0.01) or *** (*p* <0.001). The numbers above the lines indicate effect sizes and are provided for significant *post hoc* tests only. Outliers are not shown; the numbers of outliers are provided in Table 2.

Figure 8 depicts the behavior of PPG features over all analysis intervals for all recording setups. Again, for better visibility, we omitted outliers in those plots. Table 2 shows the corresponding number of outliers. The repeated measures ANOVA yielded highly significant differences (*p* < 0.001) for all features of all measurement sites and recording setups except the slope of both iPPG measurements. There, the difference was significant with *p* < 0.01.

In all measurement sites, the amplitude shows a statistically significant decrease from BL to ST1. For both contact PPG, the significant decrease can also be observed between BL and ST2. However, this behavior is not found for the iPPG measurements. There, the amplitude exhibits a significant increase between ST1 and ST2 and no significant difference between BL and ST2. The slope also decreases significantly during BL and ST1 on all measurement sites and recording setups. Only in the earlobe PPG a significant decrease between ST1 and ST2 can be observed. The area behaves similarly to the amplitude, though ST2 is significantly lower than BL for both iPPG measurements. Both iPPG and the earlobe PPG show a significant decrease in PWHA from BL to both ST1 and ST2. The finger PPG significantly increases over time.

Tables 3, 4 show the effect sizes of the reference and PPG features over the analysis intervals. We found small to medium increases for the heart rate and PP and medium to large increases for SBP and DBP. Notably, our analysis indicates large effect sizes in finger PPG for all features except PWHA, which exhibits small to medium increases between all intervals. For all other PPG measurements, the effect sizes are small.

**TABLE 3:** Effect sizes for reference features. Reported are mean effect sizes and their respective confidence interval borders (confidence level of 0.95) in brackets. Effect sizes for non-significant differences are colored grey.

Feature	BL to ST1	BL to ST2	ST1 to ST2
heart rate	0.52 [0.03, 1.00]	0.39 [−0.09, 0.87]	−0.11 [−0.59, 0.37]
SBP	0.53 [0.04, 1.02]	1.23 [0.71, 1.75]	0.65 [0.16, 1.14]
DBP	0.54 [0.05, 1.02]	1.38 [0.84, 1.90]	0.77 [0.27, 1.26]
PP	0.36 [−0.12, 0.84]	0.69 [0.20, 1.18]	0.32 [−0.17, 0.79]

**TABLE 4:** Effect sizes for PPG features. Reported are mean effect sizes and their respective confidence interval borders (confidence level of 0.95) in brackets. Effect sizes for non-significant differences are colored grey.

Feature	Measurement	BL to ST1	BL to ST2	ST1 to ST2
amplitude	PPG finger	−1.30 [−1.81, −0.79]	−1.30 [−1.81, −0.79]	0.00 [−0.46, 0.47]
amplitude	PPG earlobe	−0.24 [−0.71, 0.22]	−0.23 [−0.69, 0.24]	0.01 [−0.45, 0.47]
amplitude	iPPG forehead	−0.30 [−0.77, 0.16]	−0.14 [−0.61, 0.32]	0.16 [−0.30, 0.63]
amplitude	iPPG super	−0.25 [−0.71, 0.22]	−0.11 [−0.57, 0.36]	0.15 [−0.32, 0.61]
slope	PPG finger	−1.33 [−1.84, −0.81]	−1.36 [−1.87, −0.84]	−0.10 [−0.57, 0.36]
slope	PPG earlobe	−0.28 [−0.75, 0.18]	−0.38 [−0.84, 0.09]	−0.09 [−0.55, 0.37]
slope	iPPG forehead	−0.21 [−0.68, 0.25]	−0.17 [−0.64, 0.29]	0.05 [−0.41, 0.51]
slope	iPPG super	−0.18 [−0.64, 0.28]	−0.18 [−0.64, 0.29]	0.02 [−0.45, 0.48]
area	PPG finger	−1.20 [−1.70, −0.69]	−1.18 [−1.68, −0.67]	0.05 [−0.41, 0.52]
area	PPG earlobe	−0.35 [−0.82, 0.12]	−0.33 [−0.80, 0.14]	0.01 [−0.45, 0.48]
area	iPPG forehead	−0.43 [−0.89, 0.04]	−0.29 [−0.75, 0.18]	0.14 [−0.32, 0.61]
area	iPPG super	−0.40 [−0.87, 0.07]	−0.27 [−0.73, 0.20]	0.14 [−0.33, 0.60]
PWHA	PPG finger	0.63 [0.16, 1.11]	0.77 [0.29, 1.25]	0.12 [−0.34, 0.59]
PWHA	PPG earlobe	−0.27 [−0.73, 0.20]	−0.28 [−0.74, 0.19]	−0.00 [−0.47, 0.46]
PWHA	iPPG forehead	−0.47 [−0.94, −0.00]	−0.46 [−0.93, 0.01]	0.02 [−0.45, 0.48]
PWHA	iPPG super	−0.48 [−0.94, −0.00]	−0.48 [−0.94, −0.00]	0.00 [−0.46, 0.47]

## 5 Discussion

### 5.1 Main findings

**General response to CPT:** In general, the found behavior upon cold stress in our data matches the physiological expectation very well. Immediately after immersion, i.e., at ST1, there is a pain related increase in blood pressure and heart rate. In ST2, both, blood pressure and heart rate, remain increased but heart rate behaves less deterministic (which manifests in an increased standard deviation at ST2, see Figure 7). Our observations qualitatively and quantitatively comply to earlier studies. With respect to blood pressure, e.g., Mourot et al. report SBP increases of 14.5 and 18.1 mmHg Mourot et al. (2009), Lin et al. report 14 mmHg Lin et al. (2020), Saab et al. report 14.5 mmHg and Jauregui-Renaud et al. report 11.4 mmHg Jauregui-Renaud et al. (2001). With respect to heart rate, a more indifferent behavior or minor effects were previously reported, e.g., by Mourot et al. (2009) and Lin et al. (2020).

**Contact PPG analysis:** Our results regarding different PPG features from finger and earlobe PPG are mostly consistent with previous results as derived from our literature review. This concerns, first of all, the amplitude, which undergoes a fundamental decrease upon CPT. Slope and area also decay in finger PPG and earlobe PPG. These features show much more pronounced effects at the finger than at the earlobe. Such effects of CPT to PPG signals in general and to the considered features in particular was expected. Even local differences were expected. Though only a few studies investigated local differences upon CPT Awad et al. (2001b), Awad et al. (2006), a couple of works describe the variability of PPG features to be dependent on the measurement site indicating local differences Allen and Murray (2000); Bentham et al. (2018); Hernando et al. (2019). The found local behavior, i.e., stronger effects at the finger, reflects the high innervation of the finger vascular bed by *α*-adrenoceptors and a related responsiveness to sympathetic activation. Blood vessels at the earlobe should be affected by sympathetic activation as well, but to a lesser extend (a decreasing amplitude despite an increasing PP hints at a vascular response at the earlobe as well). The fourth feature, PWHA, turned out to be special. We therefore discuss PWHA in a separate paragraph below.

**iPPG analysis:** Our results reveal significant differences of morphological features as a response to cold stress. The behavior of the considered ROIs is thereby highly correlated. This is due to the fact that both ROIs include the forehead and forehead as well as cheeks are known to be suitable for signal extraction Lempe et al. (2013). A close look to the quantitative results suggests slightly stronger effects for the forehead ROI. This is reasonable as slightly deviating behavior of forehead and cheeks would introduce some blurring of effects. However, as the results are very similar, the following discussion does not differentiate between such ROIs.

The close relation of iPPG features to earlobe PPG underline the possibility to use iPPG for monitoring purposes beyond heart rate. Though the vast majority of available works on the iPPG focuses on heart rate, a growing number of works invokes morphological analyses. Djeldjli et al. recently presented a comparison of finger and earlobe PPG as well as iPPG Djeldjli et al. (2021). The work analyses several features including amplitude, area and PWHA during normal breathing intervals and during breath hold intervals. As in our study, their results show high correlations between earlobe PPG and iPPG during breath hold intervals for amplitude, area and PWHA^1^. Other related works do not compare iPPG directly to contact PPG but investigate the suitability of morphological features with respect to blood pressure estimation. E.g., Ding et al. showed PWHA to decay with blood pressure Ding et al. (2021). Rong and Li (2021) and Jain et al. (2016) used multiple morphological features to estimate blood pressure. Though not all such works allow statements on the specific behavior of single features, they emphasize the feasibility of morphological iPPG analysis. With respect to the competing theories on the origin of iPPG - the volumetric model as in contact PPG Moço et al. (2018) *versus* an elastic deformation model Kamshilin et al. (2015) - iPPG’s close relation to earlobe PPG in our study as well as the results of related works can be understood as strong hints that signal formation in iPPG corresponds to the volumetric model rather than to tissue compression.

Even if the global behavior of iPPG highly resembles earlobe PPG, it is worth taking a closer look because one can observe differences, which have been rarely discussed before. While features of contact PPG remain reduced at ST2, iPPG shows already an increase. The finding is not that striking as inversion in PWHA (see below), but we observed a similar effect in a different group before Fleischhauer et al. (2019). As in Fleischhauer et al. (2019), the early return to higher amplitudes after CPT in iPPG suggests that iPPG signals are driven by systemic hemodynamics and only to a lesser extent dependent on local vascular effects. Such behavior is reasonable as iPPG signals should be strongly affected by very superficial vessels. Local vasoactive vessels, in turn, contribute less to the signal formation in facial iPPG, at least as it concerns the green channel. Systematic differences between PPG and iPPG as we describe have rarely been addressed before. E.g., Djeldjli et al. (2021) rely on the premise that iPPG should resemble contact PPG. In deep learning approaches, the contact PPG can serve as target function to train deep networks to extract signals from videos Ni et al. (2021). Said premise and the usage for training are certainly valid under a “global view” but they may discard specific information available *via* iPPG. The specific behavior of iPPG renders deepened investigations and combined analyses with contact PPG to exploit their interactions for diagnostic purposes very interesting.

**PWHA:** PWHA deserves particular attention as it shows an inverse behavior between recording setups in our experiment. While PPG signals from earlobe and face show a significant decrease, finger PPG shows a significant increase in our data. PWHA is commonly related to systemic vascular resistance (SVR) Awad et al. (2007); Park et al. (2022). Marked differences in dependency to the measurement site are thus not intuitive and need detailed consideration.

Table 5 overviews previous works that invoked PWHA. Our results regarding face and earlobe PPG are in line with Ding et al., who recently reported a negative correlation of PWHA from facial iPPG and blood pressure Ding et al. (2021). Awad et al. report differences in PWHA from finger and earlobe in patients undergoing coronary artery bypass grafting Awad et al. (2001a). In contrast to our analysis, however, Awad et al. find PWHA of the earlobe PPG to be positively correlated with SBP while PWHA of the finger PPG exhibits a negative correlation. Even different from our results, Teng and Zhang (2003) describe a negative correlation between PWHA from finger PPG and SBP. Awad et al. also examined morphologic changes of finger and earlobe PPG during CPT for a healthy cohort Awad et al. (2006). They found PWHA to increase significantly during CPT for earlobe PPG and to decrease for finger PPG. Notably, none of the other features (amplitude, area, slope, downslope) of the earlobe PPG changed significantly during immersion. In contrast, the CPT significantly impacted all finger PPG features. Awad et al. explain this behavior with the blood relocating from the finger to other less vasoconstricted sites. The increased blood volume and the assumption of already maximally dilated vessels of the earlobe lead to the increase of PWHA. Again, such observations contradict our findings. An interesting detail that might explain differences in PWHA compared to our work relates to the collective’s physiological response to CPT. While blood pressure increased as in our data, pulse rate decreased in Awad et al. (2006). This might be an expression of the indifferent heart rate behavior Mourot et al. (2009) and will obviously impact PWHA. Revision of further literature shows PWHA generally to be controversial. E.g.,; Lin et al. (2020) do not find a significant change in PWHA from finger PPG on cold stress at all; Hickey et al. (2016); Abdullah et al. (2022), both conducted similar studies that invoked arm lowering and elevation. While Hickey et al. describe a significant decrease with lowering and a mild (non-significant) increase with elevation, Abdullah et al. (2022) show a significant decrease with elevation and no effect upon lowering (see Figure 2 in Abdullah et al. (2022)). Lastly, even in Djeldjli’s work Djeldjli et al. (2021) PWHA stands out. Compared to all other temporal features, PWHA yields a clearly reduced correlation between finger and earlobe PPG during breath hold intervals.

To conclude, the literature on PWHA is not consistent. We thus cannot regard our own results as either plausible or implausible in face of the literature. We tried to rule out problems related to the processing by going through all templates and did not find abnormalities. As stated before, signal quality can cause problems (e.g., see Figure 9 for an example of low-quality template generation in finger PPG) but is not likely to behave systematically and cause the found inverse effect. Even the existence of a diastolic wave might cause misleading results. Depending on whether it is more or less pronounced, half amplitude could be reached before or after it. But again, a systematic effect as it would be needed to cause our results is not likely. We thus believe that the found behavior reflects a physiological effect in our cohort. Our hypothesis to explain such behavior is as follows. Assuming that the recorded finger PPG integrates contributions of larger vessels, which are less affected by vasoconstriction, a strong increase in arterial vessels downstream to the measurement site could explain an increase in PWHA. Local vasoconstriction in the facial region is less pronounced. The systemically increased blood pressure could lead to higher arterial volumes and vessel tension during systole, which favor the outflow and cause inverse effects on PWHA, i.e., a reduction. The common opinion that PWHA relates to SVR thus might not be generally valid but local factors might have a strong influence. Notably, this finding might have relevance beyond PWHA. If our hypothesis is true, other PPG based features are likely to be similarly affected by local factors but this assumption needs deepened investigations. One approach to deepen the understanding could make use of simulations. Recently, models and simulations of light-tissue interactions in the context of PPG have gained immense interest. Most of these works use Monte Carlo simulations to analyze the impact of contributing factors to the PPG morphology (e.g., sensor geometry Chatterjee and Kyriacou (2019), obesity Ajmal et al. (2021); Boonya-ananta et al. (2021) and skin tone Ajmal et al. (2021)). The used models allow for the simulation of pulsating blood to varying degrees, but they do not incorporate details on interlinked changes of systemic and local hemodynamics (i.e., blood pressure and local vessel properties). While the existing models thus serve to prove some of our experimental findings (e.g., as the lowered quality of red channel Ajmal et al. (2021)), further extensions are required to simulate the behavior of complex parameters like PWHA under varying systemic and local conditions.

**TABLE 5:** Details on selected studies that used PWHA. ^†^More subjects were invoked in another part of the study. ^‡^The correlation holds if wavelet processing was included. CO - cardiac output.

Reference	Population/experiment	Site	Main finding
Awad et al. (2007)	14, coronary artery bypass grafting	finger	correlation to SVR 0.56
		earlobe	correlation to SVR 0.62
Awad et al. (2006)	12, healthy, reaction upon CPT	finger	significant decrease upon CPT
		earlobe	significant increase upon CPT
	12, coronary artery bypass grafting	earlobe	inverse correlation to CO (−0.761)
Awad et al. (2001a)	10^†^, coronary artery bypass grafting	finger	negative correlation to SBP (−0.1)
		earlobe	correlation to SBP (0.8)
Teng and Zhang (2003)	15, healthy, reaction upon activity	finger	negative correlation to SBP (−0.732^†^)
Ding et al. (2021)	12, healthy, deep breathing/exercise	face (iPPG)	negative correlation to SBP ( <−0.7 for 80% of subjects)
Lin et al. (2020)	12, reaction upon CPT	finger	no significant change
Hickey et al. (2016)	20, healthy, arm lowering/elevation	finger	significant decrease with lowering
Abdullah et al. (2022)	15, healthy, arm lowering/elevation	finger	significant decrease with elevation

### 5.2 Limitations

Our investigation has some limitations, which we discuss in this section.

**FIGURE 10:**
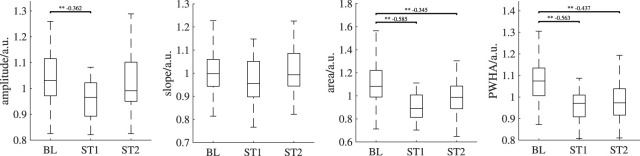
Behavior of iPPG features of the forehead using the red channel over all analysis intervals. If significant, *post hoc* tests’ outcome is denoted by * (*p* <0.05), ** (*p* <0.01) or *** (*p* <0.001). The numbers above the lines indicate effect sizes and are provided for significant *post hoc* tests only. Outliers are not shown.

First, we have decided to restrict our PPG analyses to finger PPG and earlobe PPG. iPPG analyses were restricted to the green channel and to two ROIs (the forehead and a combination of forehead and cheeks). Our selection was kind of obvious, as finger and earlobe are common choices in PPG. For iPPG, the green channel as a single channel Verkruysse et al. (2008) and the forehead as well as the cheeks Lempe et al. (2013); Kim et al. (2021) are known to yield good results. However, the experimental setup features much more possibilities. Particularly with respect to iPPG, alternating ROI definitions including additional constraints, e.g., regarding homogeneity as in Woyczyk et al. (2021), other color spaces Ernst et al. (2021) and combinations of color channels like POS Wang et al. (2017) and CHROM de Haan and Jeanne (2013) are possible and might add valuable insights. We experimentally did some first tests on the red channel. The higher wavelength allows for a deeper penetration offering the potential for further considerations on prevailing physiological mechanisms. Figure 10 shows the result. According to the expectation from previous works and simulations Ajmal et al. (2021), the red channel generally has reduced signal quality but the found behavior resembles the green channel’s behavior. However, in-depth analyses and more sophisticated processing strategies are required to exploit the added value of the red channel. Such tasks should definitely be considered in the future.

Second, we restricted our analysis to a reduced number of features. In fact, multiple other features would be of interest and possible. Owing to their pathophysiological relevance, particularly features from the second derivative seem to be interesting. Deriving such features is possible Fleischhauer et al. (2020) but they are easily affected by distortions as also seen in Djeldjli et al. (2021). In order to avoid distorted features to interfere with the physiological interpretation, we selected the features that are common and that we believe to be robust at the same time. Again, other choices should be considered in the future.

Third, the presented research includes a comparatively high number of subject but healthy volunteers only. In elderly or under pathological conditions, the observed behavior might differ as seen in previous works Cooke et al. (1993); Jaryal et al. (2009); Kurki et al. (1990). Particularly with respect to the idea of combining measurement sites or acquisition modes in order to refine diagnostics, this is of importance and obviously deserves special attention in the future.

### 5.3 Conclusion and outlook

To summarize, we showed significant changes of PPG signals in all recording setups upon cold stress. Remarkably, even in iPPG, the considered features show an effect. This finding underlines the opportunity to use iPPG beyond heart rate. Despite the existence of a response in all recording setups, there are differences. Such differences generally relate to the effect sizes. Finger PPG shows the strongest effects, which we attribute to a marked vasoconstriction in the finger. For PWHA, differences do not only concern the effect size but the general behavior. For PWHA, we found an inverse behavior between finger and earlobe. Further, iPPG features tend to an earlier return after a first response than PPG features, which could be an indicator of iPPG’s formation to which very small superficial vessels contribute more than in contact PPG.

Taken together, the found differences carry at least three concrete implications for the usage of PPG and future works. First, in multisite PPG, care has to be taken because indifferent shape changes will also affect fiducial points and can thus hamper the analysis as well as interpretation of time delays between PPG signals. Second, care should be taken when earlier findings or even methods, e.g., pretrained machine learning methods, are transferred between recording setups. Third, the existence/absence of differences in recording setups might carry diagnostic information. A future diagnostic usage is of high interest but requires methodological developments and basic research invoking different subject groups.

## Data Availability

The original contributions presented in the study are included in the article/Supplementary Materials, further inquiries can be directed to the corresponding author.

## Footnotes

1 Note that normal breathing intervals in Djeldjli et al. (2021) partially suffered from lacking feature variability; as correlation in such cases is misleading, our statements here only relate to results for breath hold intervals.
