# Supplementary material for: Photoplethysmography upon cold stress—impact of measurement site and acquisition mode
Source: Front Physiol. 2023 Jun 1;14:1127624. doi: 10.3389/fphys.2023.1127624 (PMC10267461; doi:10.3389/fphys.2023.1127624)
Supplement: Supplementary file 1 [file DataSheet1.PDF]

Supplementary Material

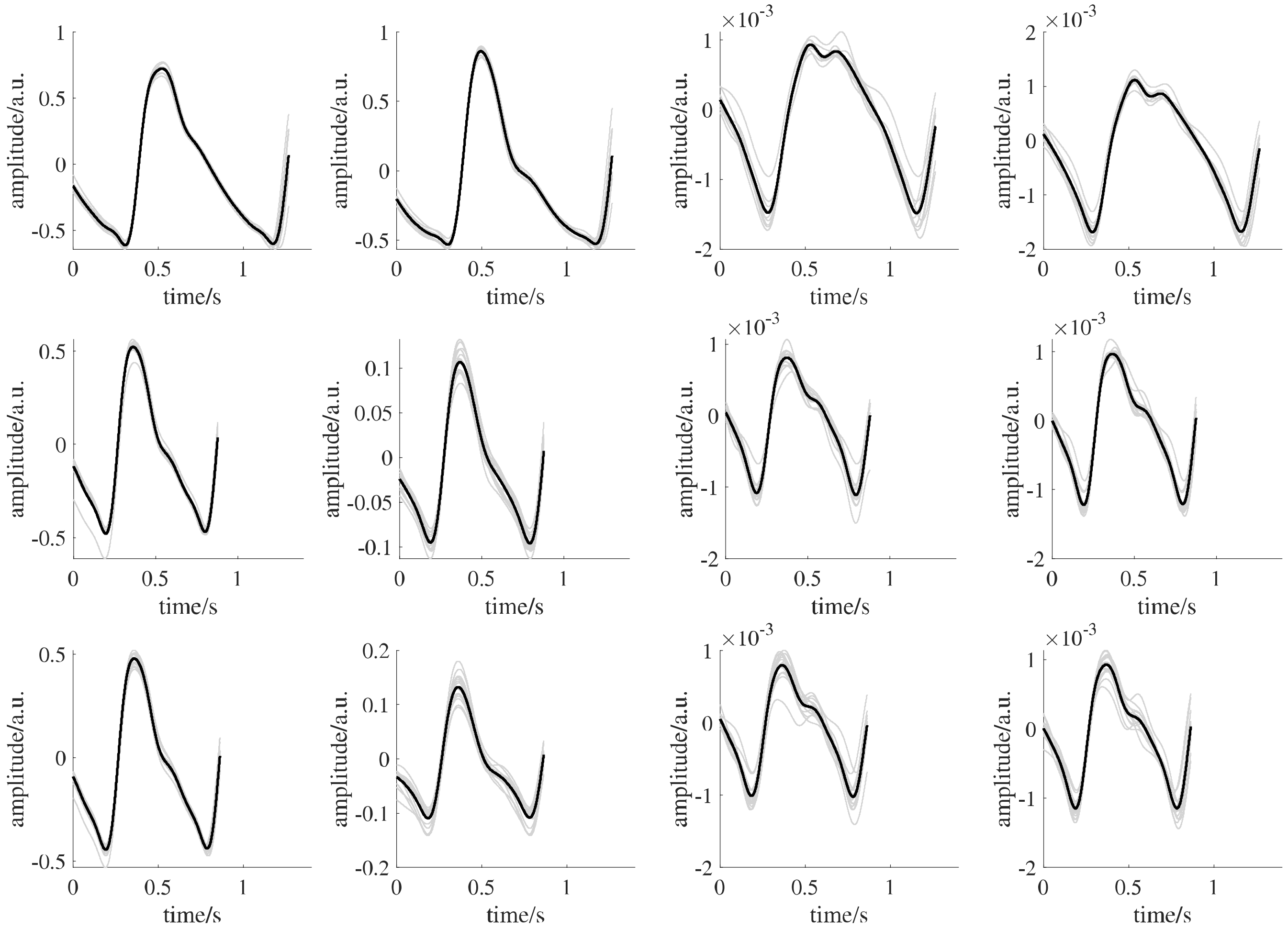

Figure S1. Template generation of one subject. From left to right: earlobe PPG (BL: 10 beats, ST1: 15 beats, ST2: 16 beats), finger PPG (BL: 10 beats, ST1: 15 beats, ST2: 15 beats), forehead iPPG (BL: 10 beats, ST1: 16 beats, ST2: 15 beats), super ROI iPPG (BL: 10 beats, ST1: 15 beats, ST2: 15 beats). The upper row shows signals during BL. The middle row shows signals during ST1. The lower row shows signals during ST2. Black lines indicate mean beat templates; gray lines indicate the corresponding beat segments.

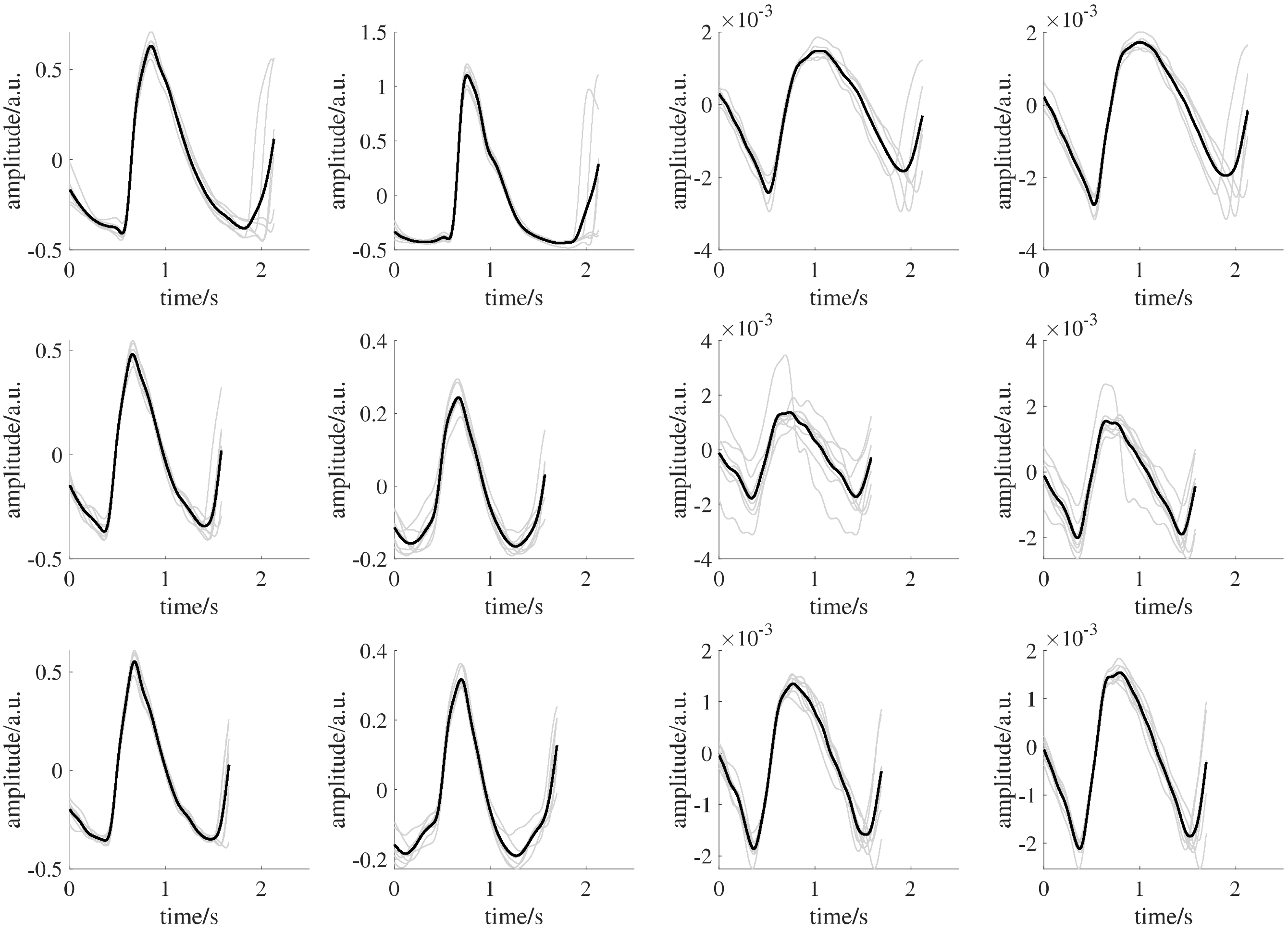

Figure S2. Template generation of one subject. From left to right: earlobe PPG (BL: 6 beats, ST1: 8 beats, ST2: 7 beats), finger PPG (BL: 6 beats, ST1: 8 beats, ST2: 7 beats), forehead iPPG (BL: 5 beats, ST1: 8 beats, ST2: 8 beats), super ROI iPPG (BL: 5 beats, ST1: 8 beats, ST2: 8 beats). The upper row shows signals during BL. The middle row shows signals during ST1. The lower row shows signals during ST2. Black lines indicate mean beat templates; gray lines indicate the corresponding beat segments.

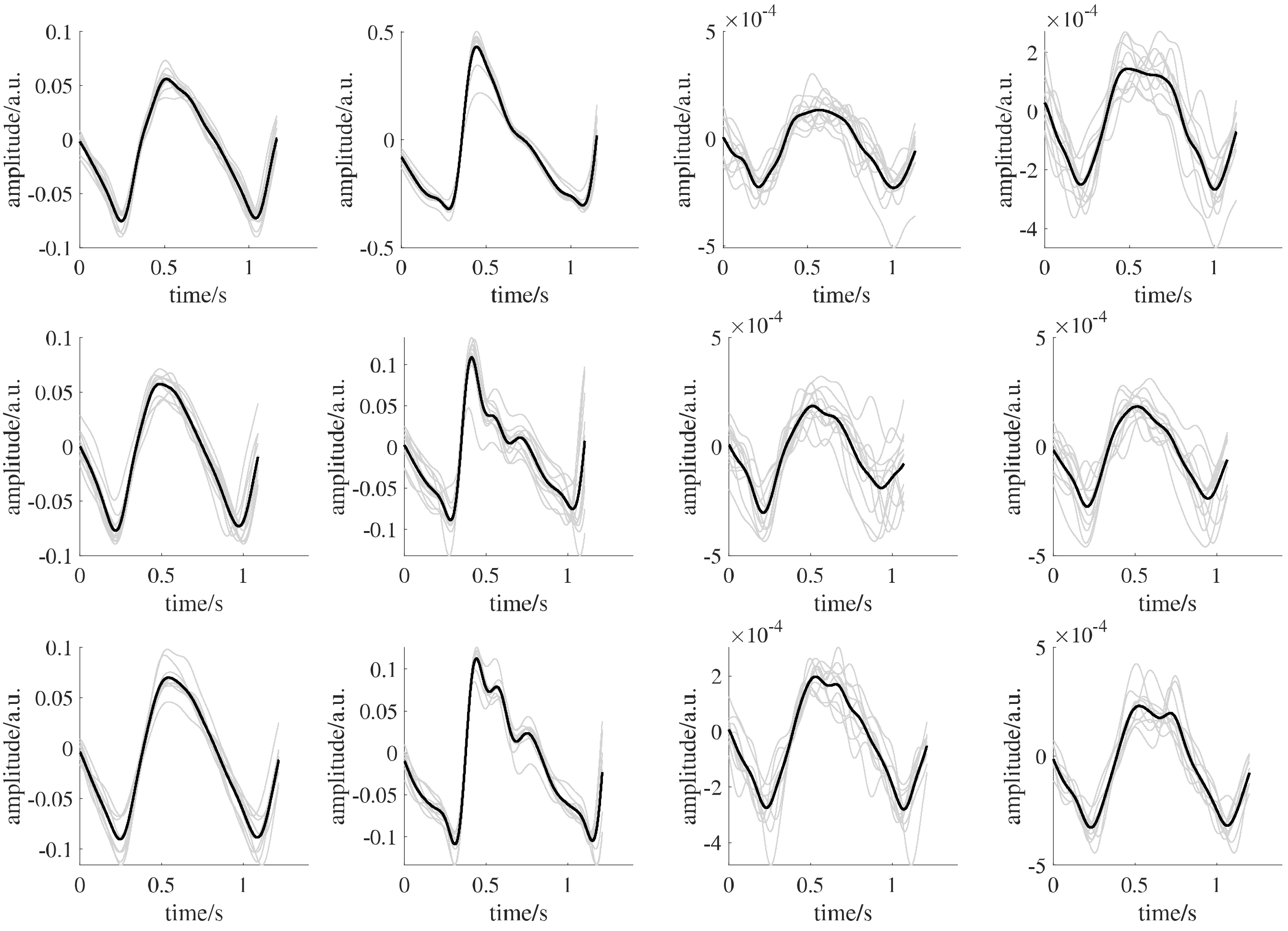

Figure S3. Template generation of one subject. From left to right: earlobe PPG (BL: 11 beats, ST1: 12 beats, ST2: 10 beats), finger PPG (BL: 11 beats, ST1: 12 beats, ST2: 10 beats), forehead iPPG (BL: 12 beats, ST1: 12 beats, ST2: 10 beats), super ROI iPPG (BL: 12 beats, ST1: 12 beats, ST2: 10 beats). The upper row shows signals during BL. The middle row shows signals during ST1. The lower row shows signals during ST2. Black lines indicate mean beat templates; gray lines indicate the corresponding beat segments.

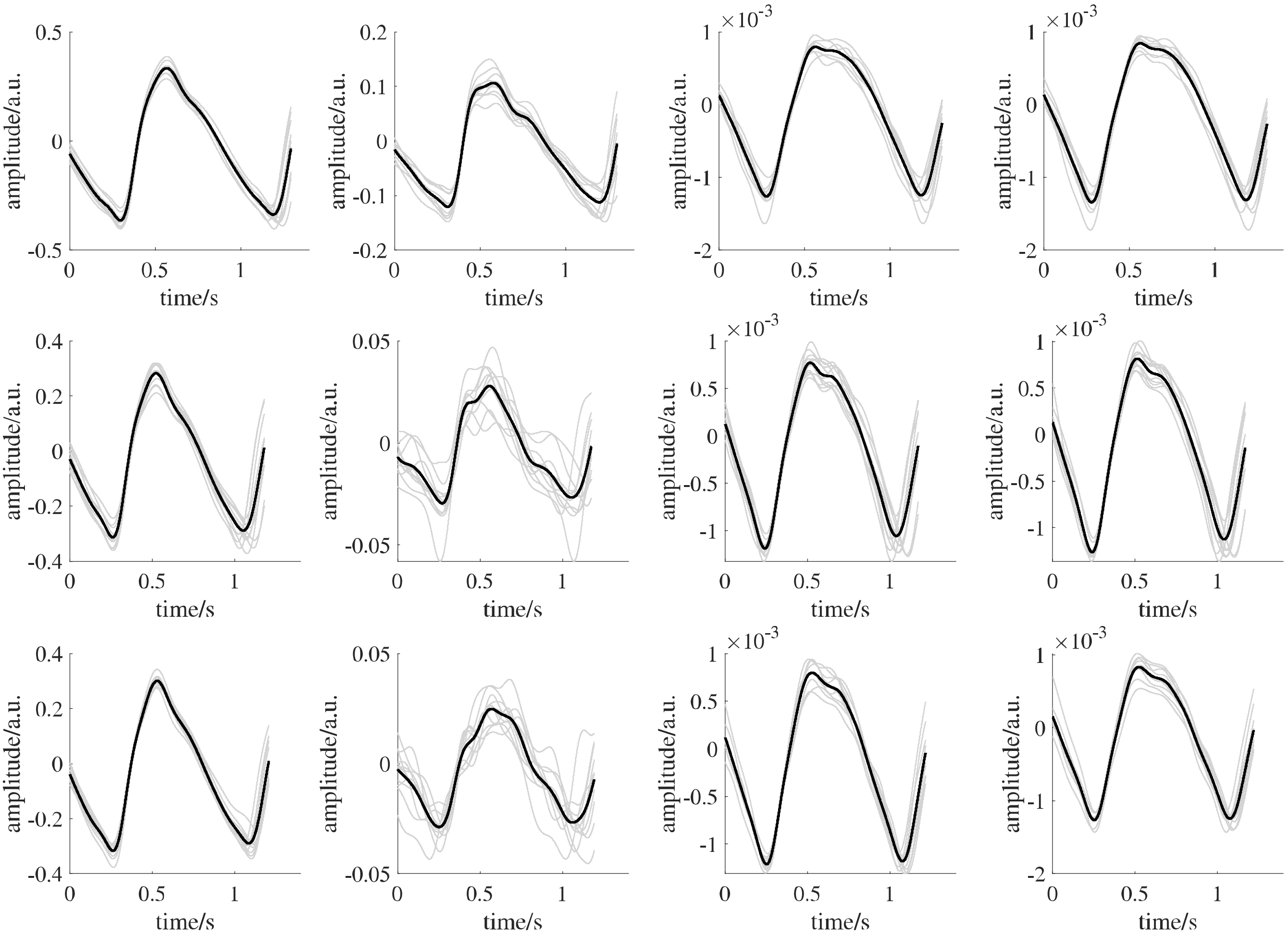

Figure S4. Template generation of one subject. From left to right: earlobe PPG (BL: 10 beats, ST1: 11 beats, ST2: 11 beats), finger PPG (BL: 10 beats, ST1: 11 beats, ST2: 11 beats), forehead iPPG (BL: 9 beats, ST1: 11 beats, ST2: 10 beats), super ROI iPPG (BL: 9 beats, ST1: 11 beats, ST2: 10 beats). The upper row shows signals during BL. The middle row shows signals during ST1. The lower row shows signals during ST2. Black lines indicate mean beat templates; gray lines indicate the corresponding beat segments.

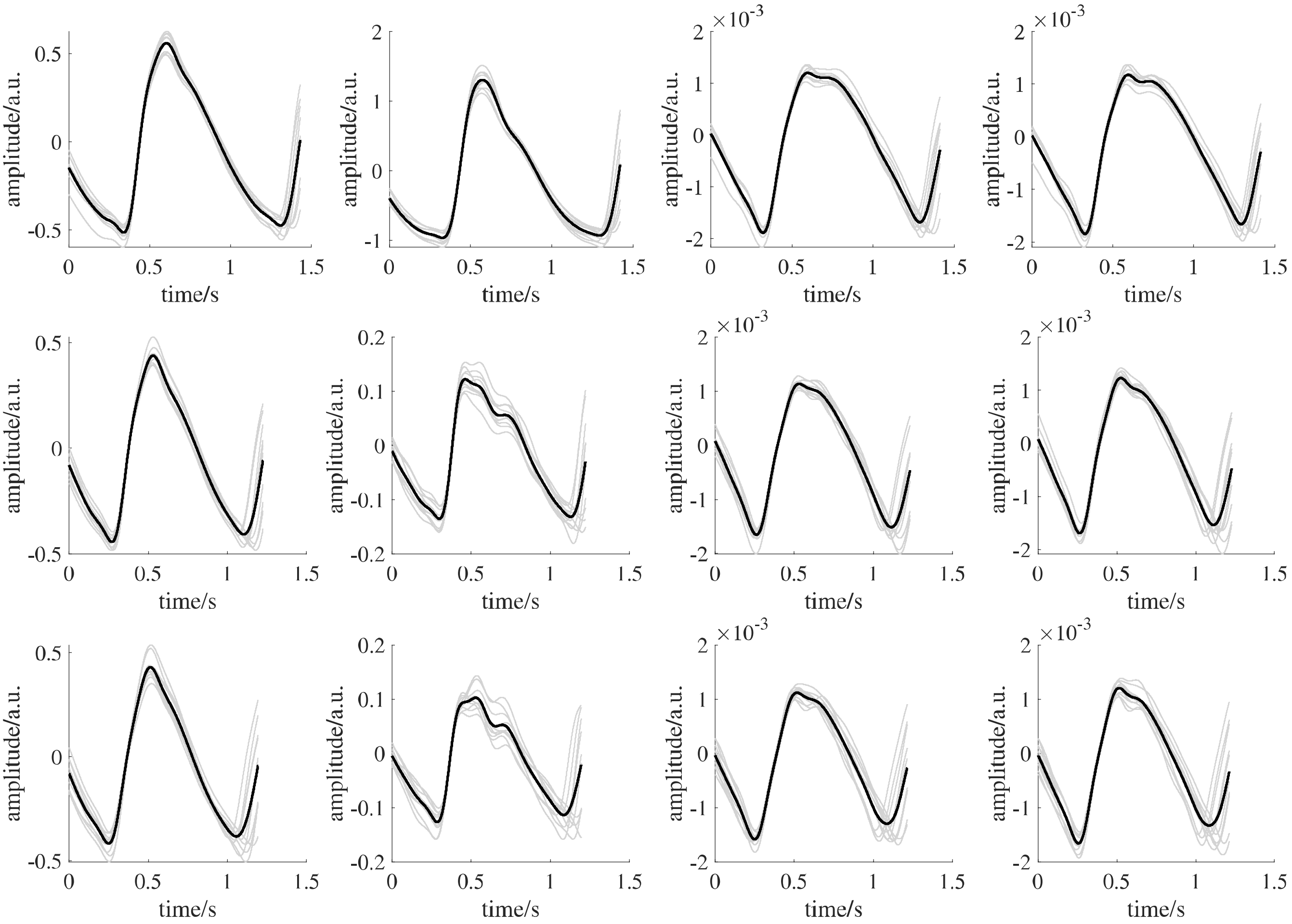

Figure S5. Template generation of one subject. From left to right: earlobe PPG (BL: 9 beats, ST1: 10 beats, ST2: 10 beats), finger PPG (BL: 9 beats, ST1: 10 beats, ST2: 10 beats), forehead iPPG (BL: 9 beats, ST1: 10 beats, ST2: 11 beats), super ROI iPPG (BL: 9 beats, ST1: 10 beats, ST2: 11 beats). The upper row shows signals during BL. The middle row shows signals during ST1. The lower row shows signals during ST2. Black lines indicate mean beat templates; gray lines indicate the corresponding beat segments.

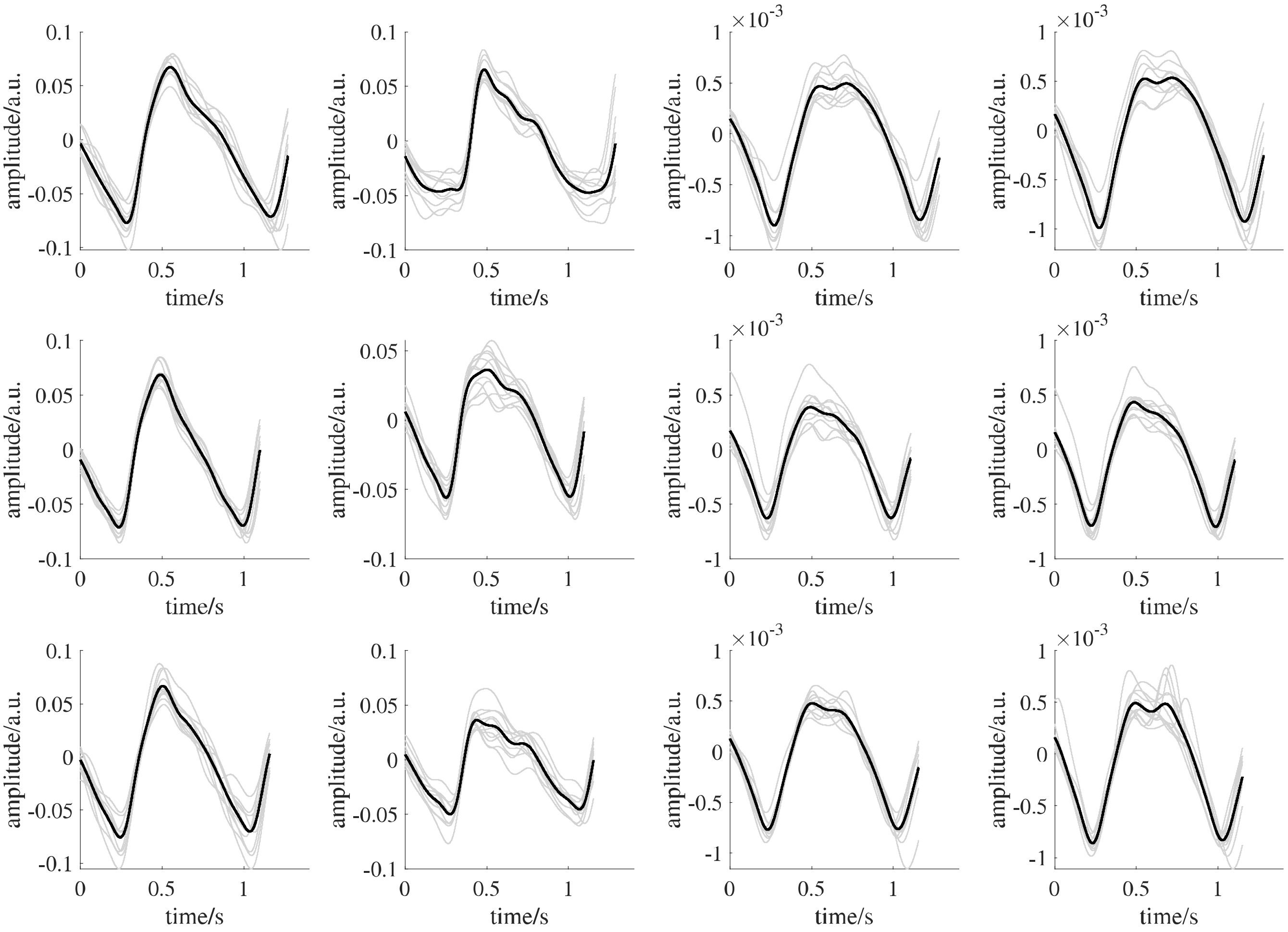

Figure S6. Template generation of one subject. From left to right: earlobe PPG (BL: 10 beats, ST1: 12 beats, ST2: 11 beats), finger PPG (BL: 10 beats, ST1: 12 beats, ST2: 11 beats), forehead iPPG (BL: 10 beats, ST1: 11 beats, ST2: 11 beats), super ROI iPPG (BL: 10 beats, ST1: 11 beats, ST2: 11 beats). The upper row shows signals during BL. The middle row shows signals during ST1. The lower row shows signals during ST2. Black lines indicate mean beat templates; gray lines indicate the corresponding beat segments.

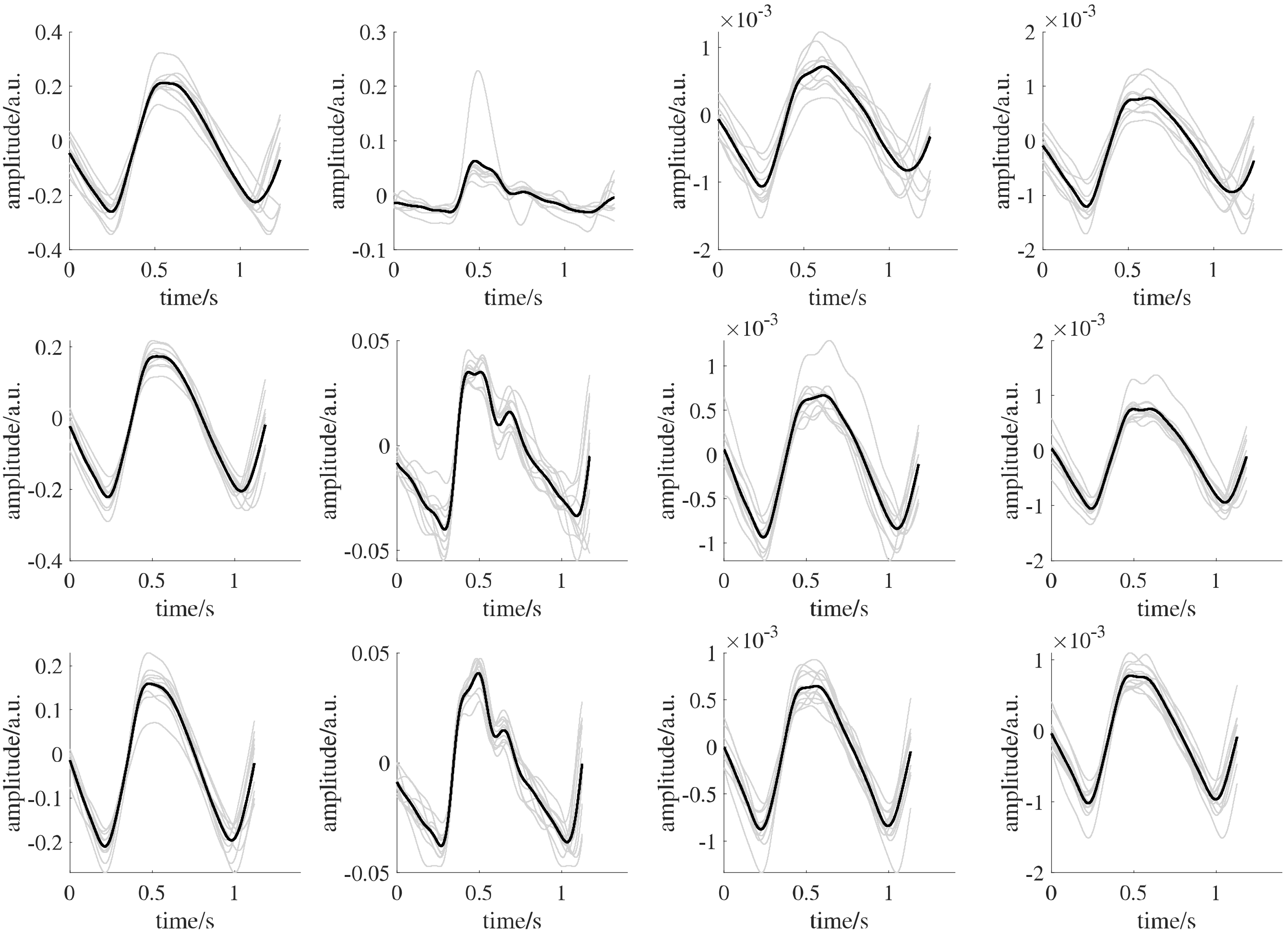

Figure S7. Template generation of one subject. From left to right: earlobe PPG (BL: 10 beats, ST1: 11 beats, ST2: 11 beats), finger PPG (BL: 9 beats, ST1: 11 beats, ST2: 11 beats), forehead iPPG (BL: 10 beats, ST1: 11 beats, ST2: 12 beats), super ROI iPPG (BL: 10 beats, ST1: 11 beats, ST2: 12 beats). The upper row shows signals during BL. The middle row shows signals during ST1. The lower row shows signals during ST2. Black lines indicate mean beat templates; gray lines indicate the corresponding beat segments.

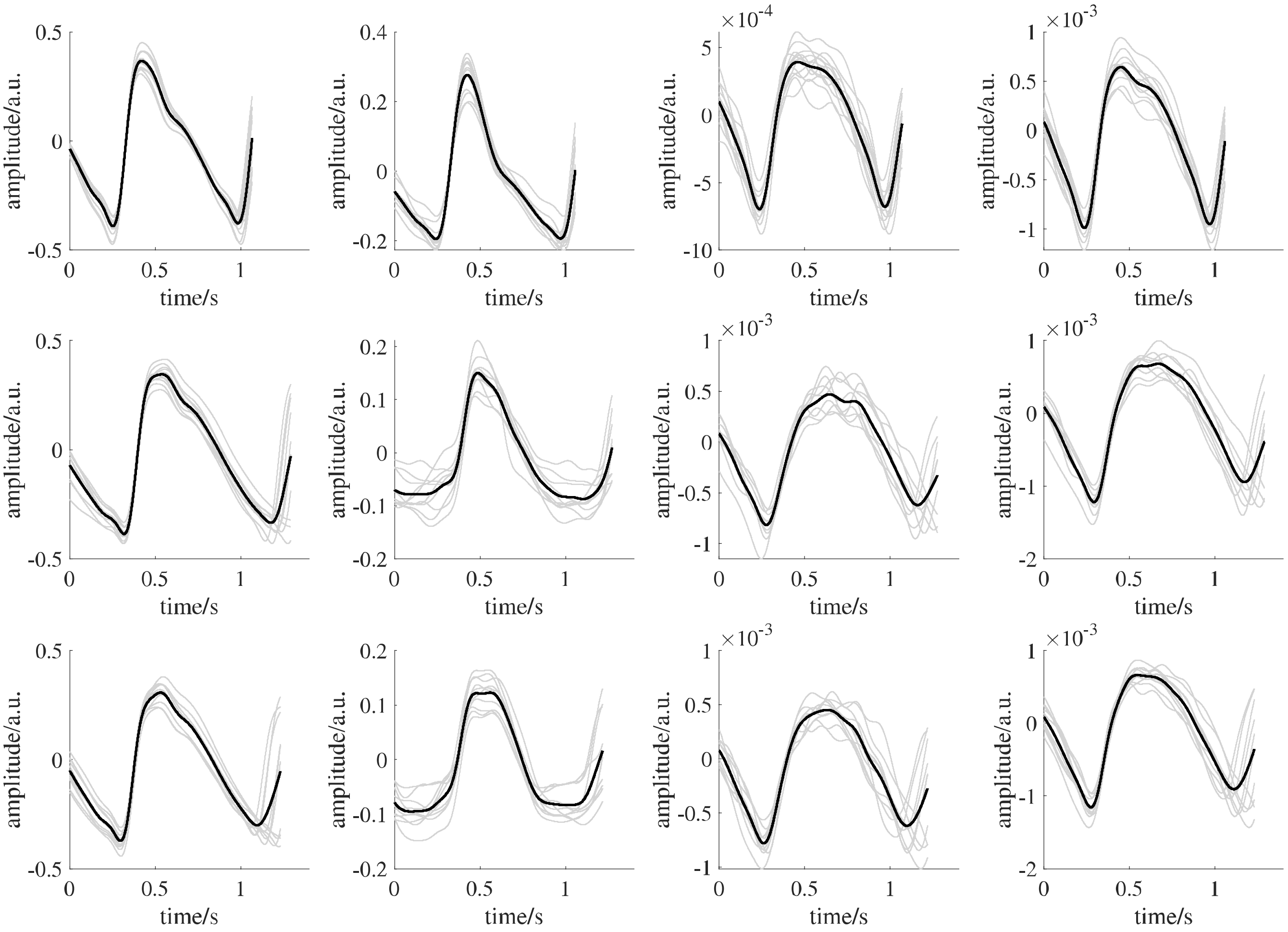

Figure S8. Template generation of one subject. From left to right: earlobe PPG (BL: 12 beats, ST1: 10 beats, ST2: 10 beats), finger PPG (BL: 12 beats, ST1: 10 beats, ST2: 10 beats), forehead iPPG (BL: 13 beats, ST1: 9 beats, ST2: 10 beats), super ROI iPPG (BL: 13 beats, ST1: 9 beats, ST2: 10 beats). The upper row shows signals during BL. The middle row shows signals during ST1. The lower row shows signals during ST2. Black lines indicate mean beat templates; gray lines indicate the corresponding beat segments.

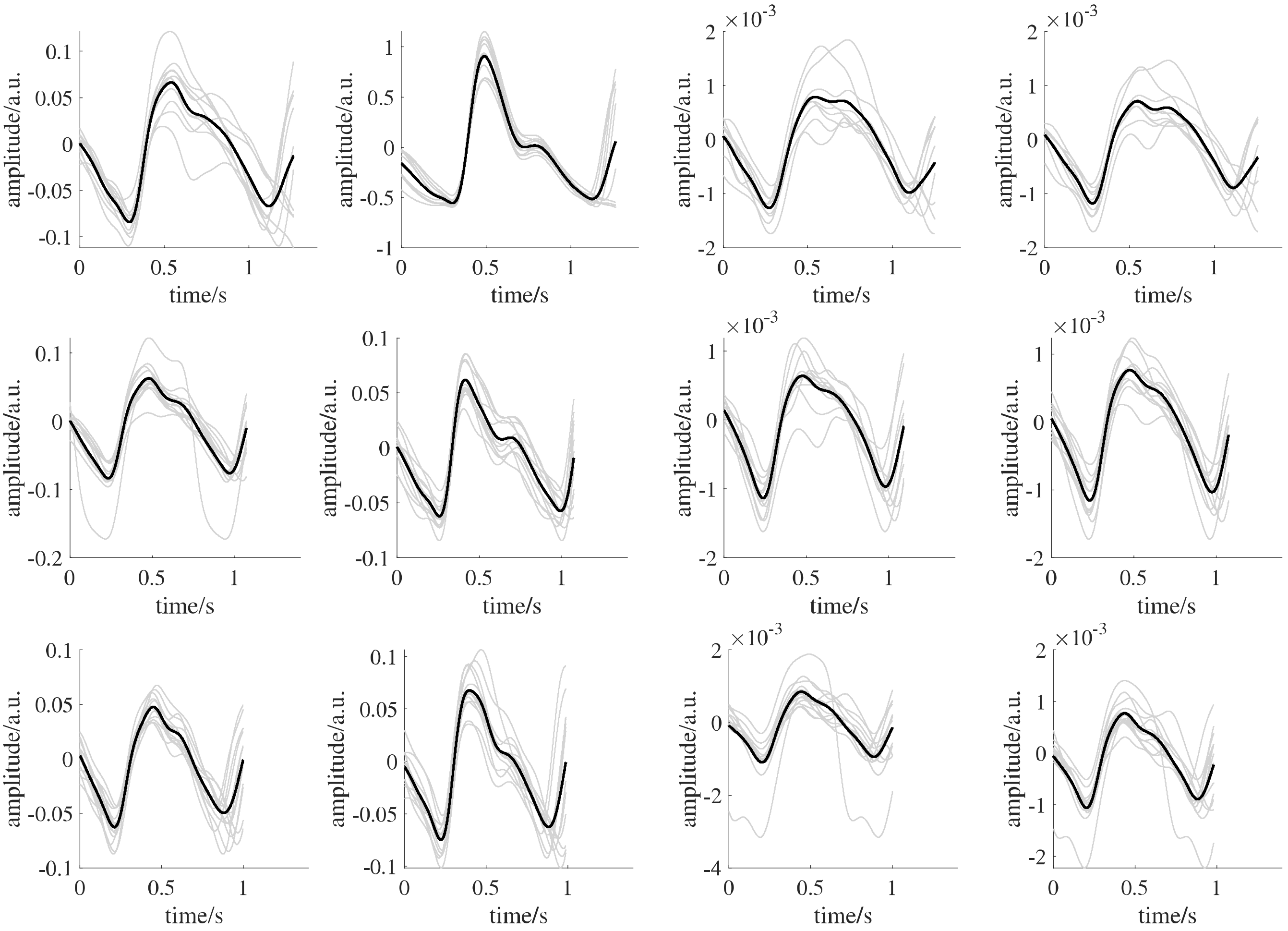

Figure S9. Template generation of one subject. From left to right: earlobe PPG (BL: 10 beats, ST1: 12 beats, ST2: 13 beats), finger PPG (BL: 10 beats, ST1: 12 beats, ST2: 13 beats), forehead iPPG (BL: 9 beats, ST1: 12 beats, ST2: 13 beats), super ROI iPPG (BL: 9 beats, ST1: 12 beats, ST2: 13 beats). The upper row shows signals during BL. The middle row shows signals during ST1. The lower row shows signals during ST2. Black lines indicate mean beat templates; gray lines indicate the corresponding beat segments.

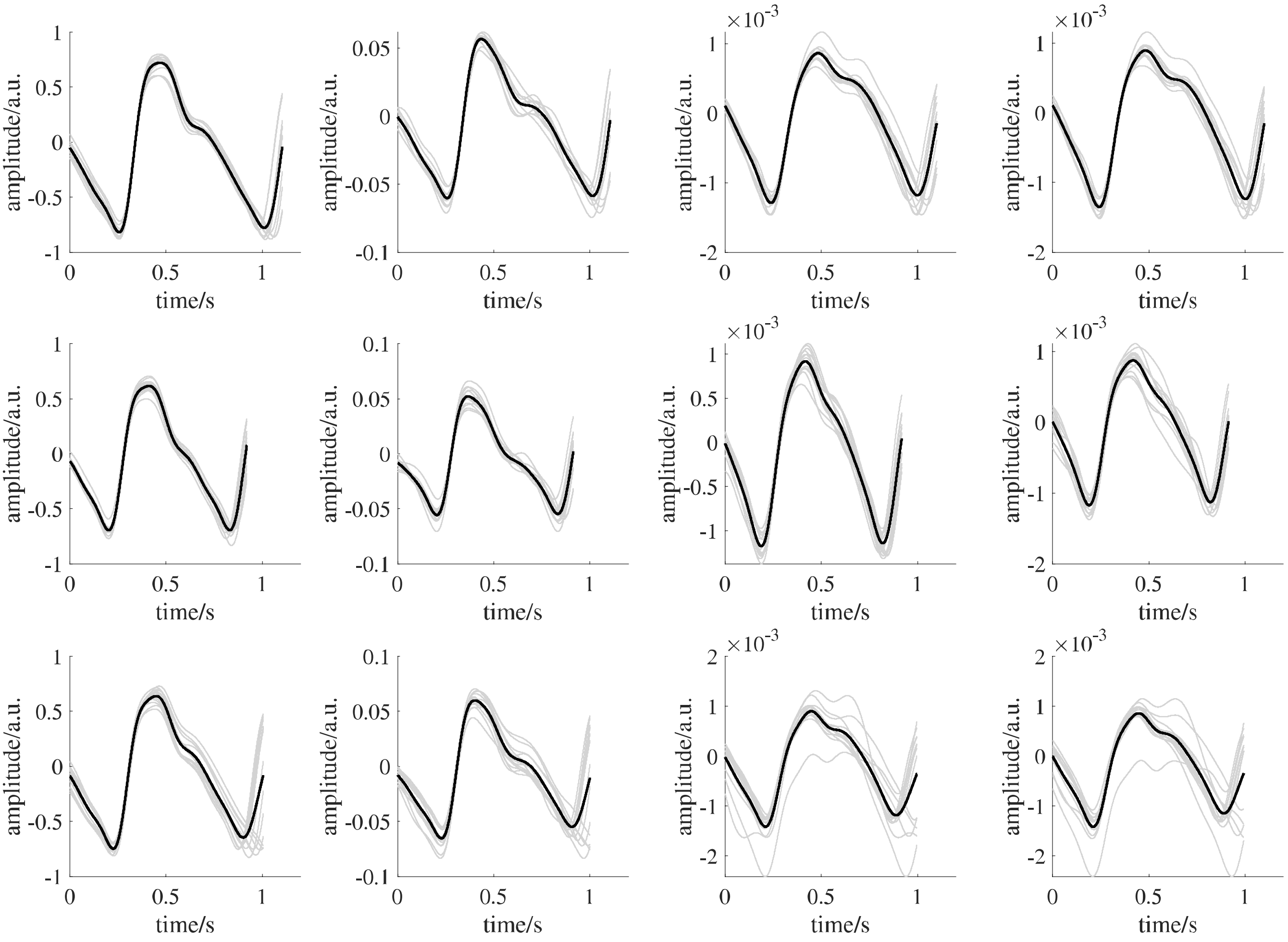

Figure S10. Template generation of one subject. From left to right: earlobe PPG (BL: 11 beats, ST1: 14 beats, ST2: 13 beats), finger PPG (BL: 11 beats, ST1: 14 beats, ST2: 13 beats), forehead iPPG (BL: 12 beats, ST1: 14 beats, ST2: 13 beats), super ROI iPPG (BL: 12 beats, ST1: 14 beats, ST2: 13 beats). The upper row shows signals during BL. The middle row shows signals during ST1. The lower row shows signals during ST2. Black lines indicate mean beat templates; gray lines indicate the corresponding beat segments.

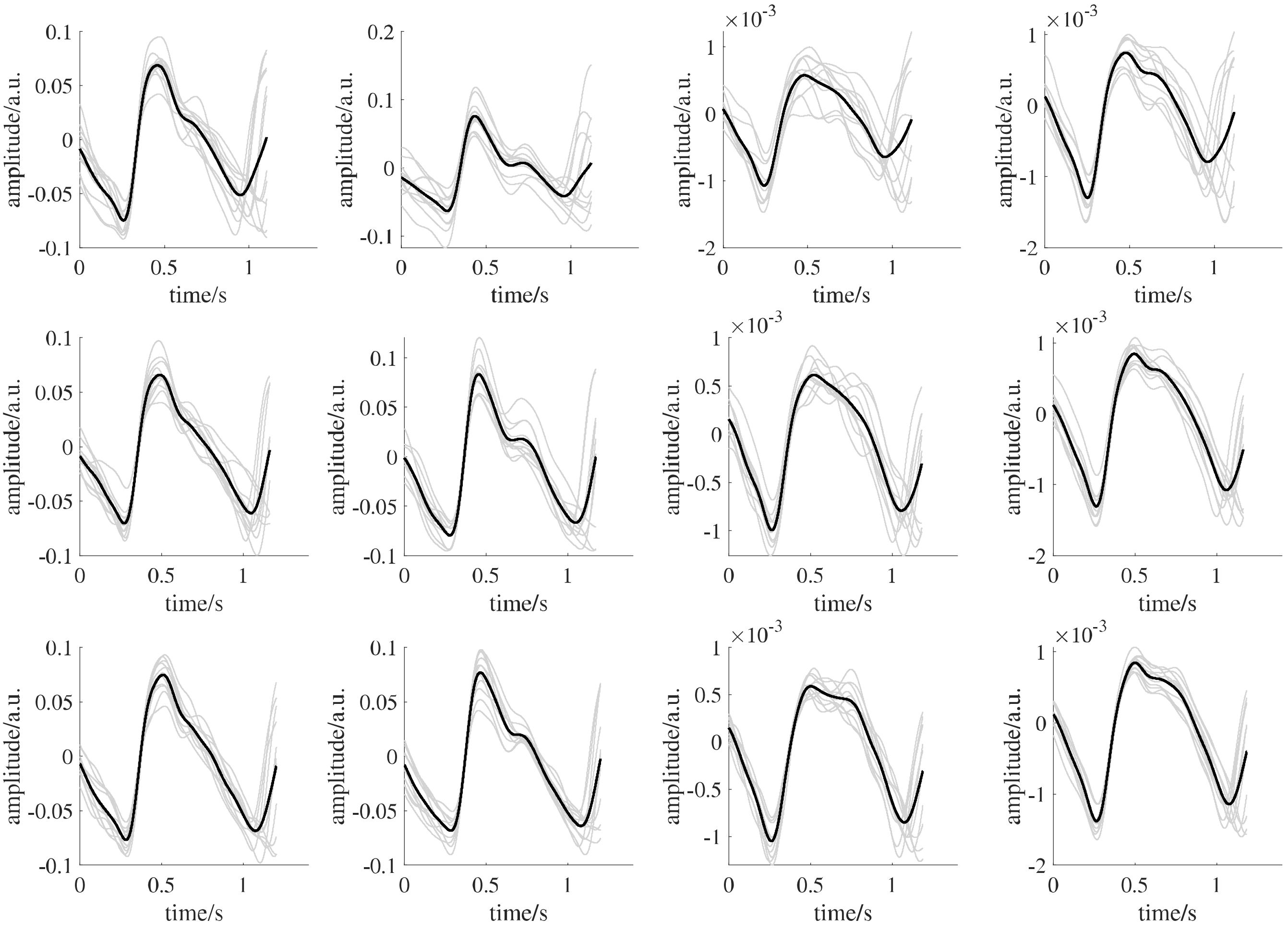

Figure S11. Template generation of one subject. From left to right: earlobe PPG (BL: 12 beats, ST1: 11 beats, ST2: 11 beats), finger PPG (BL: 12 beats, ST1: 11 beats, ST2: 11 beats), forehead iPPG (BL: 11 beats, ST1: 11 beats, ST2: 11 beats), super ROI iPPG (BL: 11 beats, ST1: 11 beats, ST2: 11 beats). The upper row shows signals during BL. The middle row shows signals during ST1. The lower row shows signals during ST2. Black lines indicate mean beat templates; gray lines indicate the corresponding beat segments.

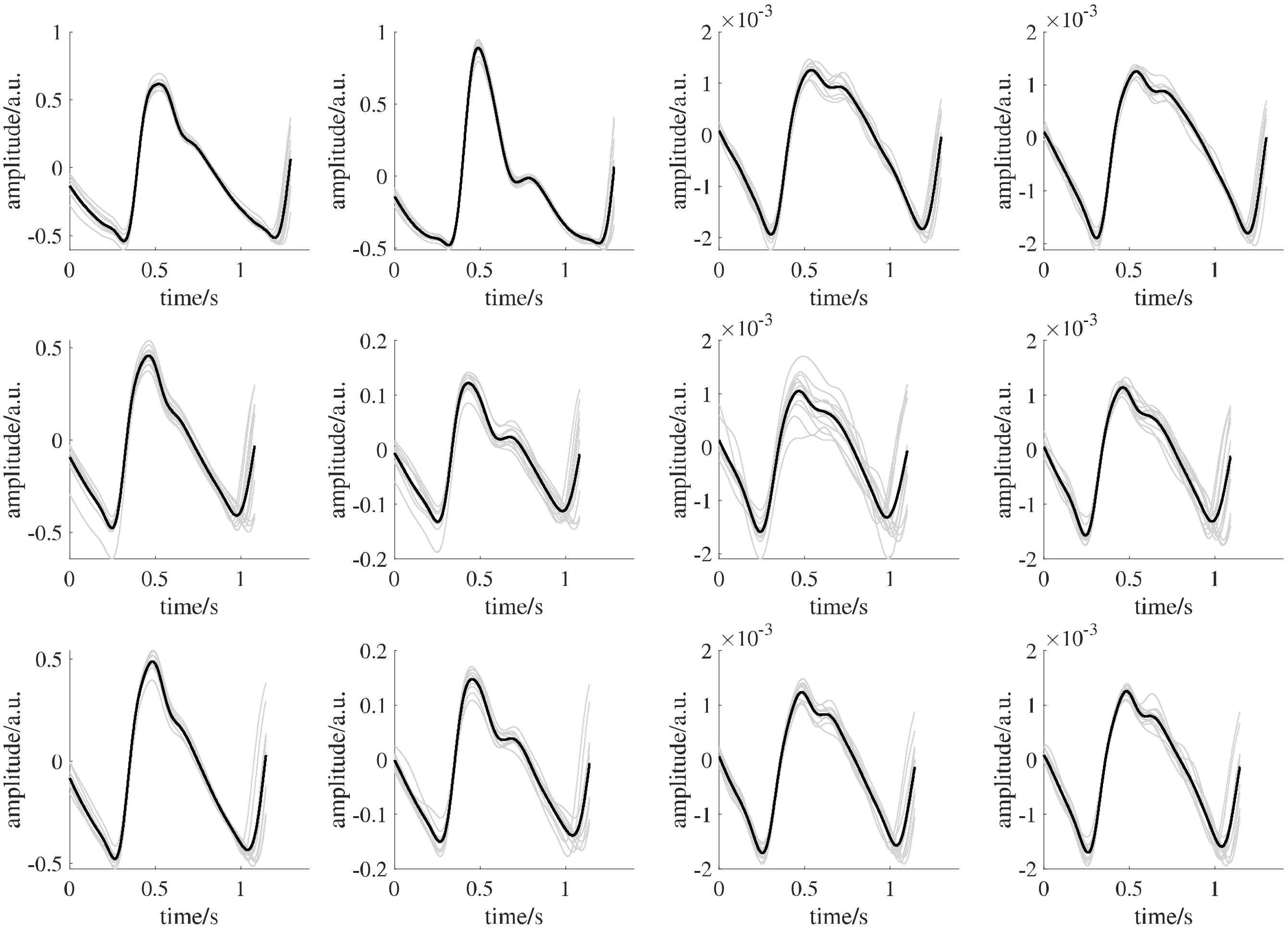

Figure S12. Template generation of one subject. From left to right: earlobe PPG (BL: 10 beats, ST1: 12 beats, ST2: 11 beats), finger PPG (BL: 10 beats, ST1: 12 beats, ST2: 11 beats), forehead iPPG (BL: 10 beats, ST1: 12 beats, ST2: 12 beats), super ROI iPPG (BL: 10 beats, ST1: 12 beats, ST2: 12 beats). The upper row shows signals during BL. The middle row shows signals during ST1. The lower row shows signals during ST2. Black lines indicate mean beat templates; gray lines indicate the corresponding beat segments.

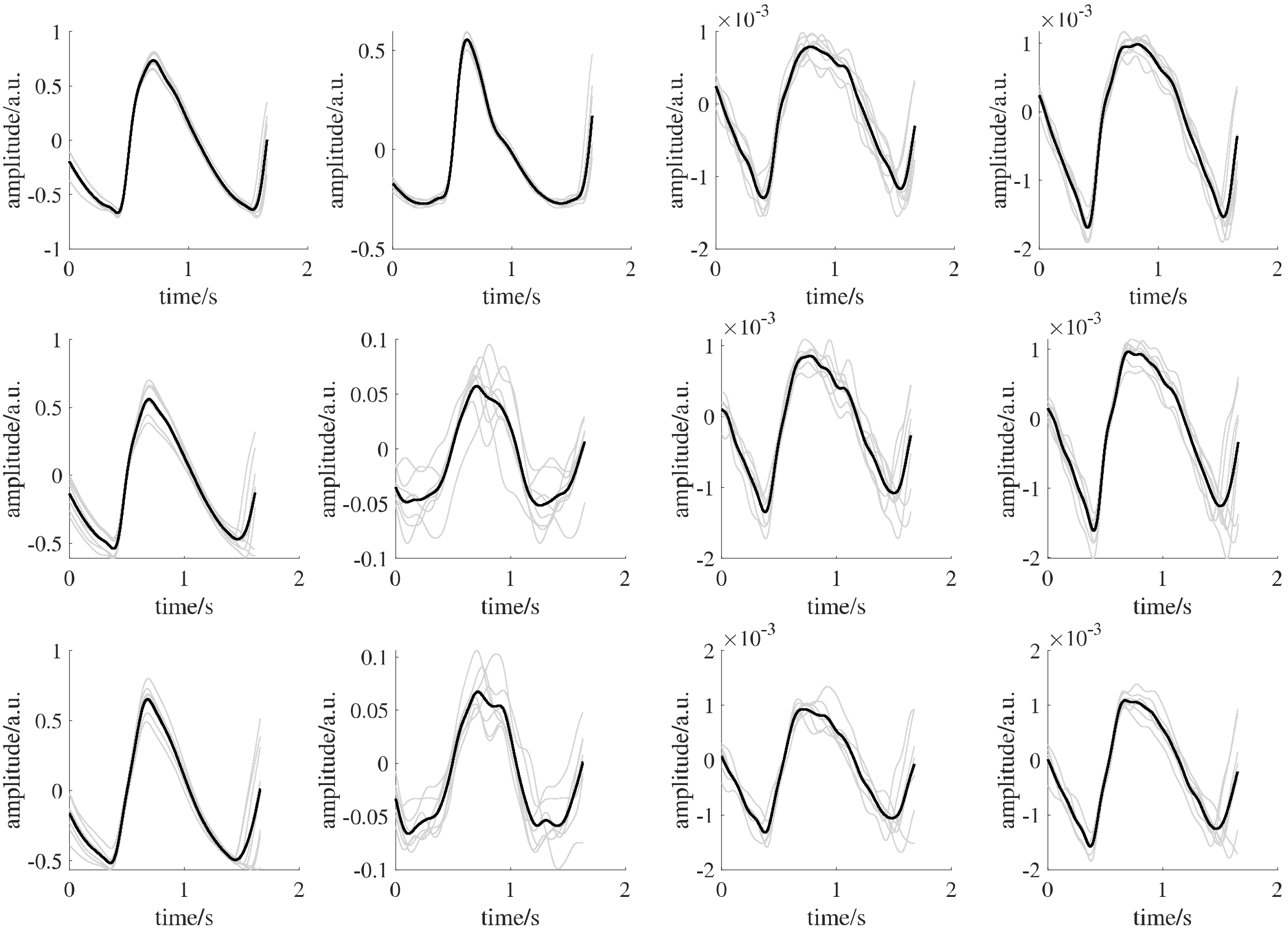

Figure S13. Template generation of one subject. From left to right: earlobe PPG (BL: 7 beats, ST1: 8 beats, ST2: 8 beats), finger PPG (BL: 7 beats, ST1: 8 beats, ST2: 7 beats), forehead iPPG (BL: 8 beats, ST1: 8 beats, ST2: 7 beats), super ROI iPPG (BL: 8 beats, ST1: 8 beats, ST2: 7 beats). The upper row shows signals during BL. The middle row shows signals during ST1. The lower row shows signals during ST2. Black lines indicate mean beat templates; gray lines indicate the corresponding beat segments.

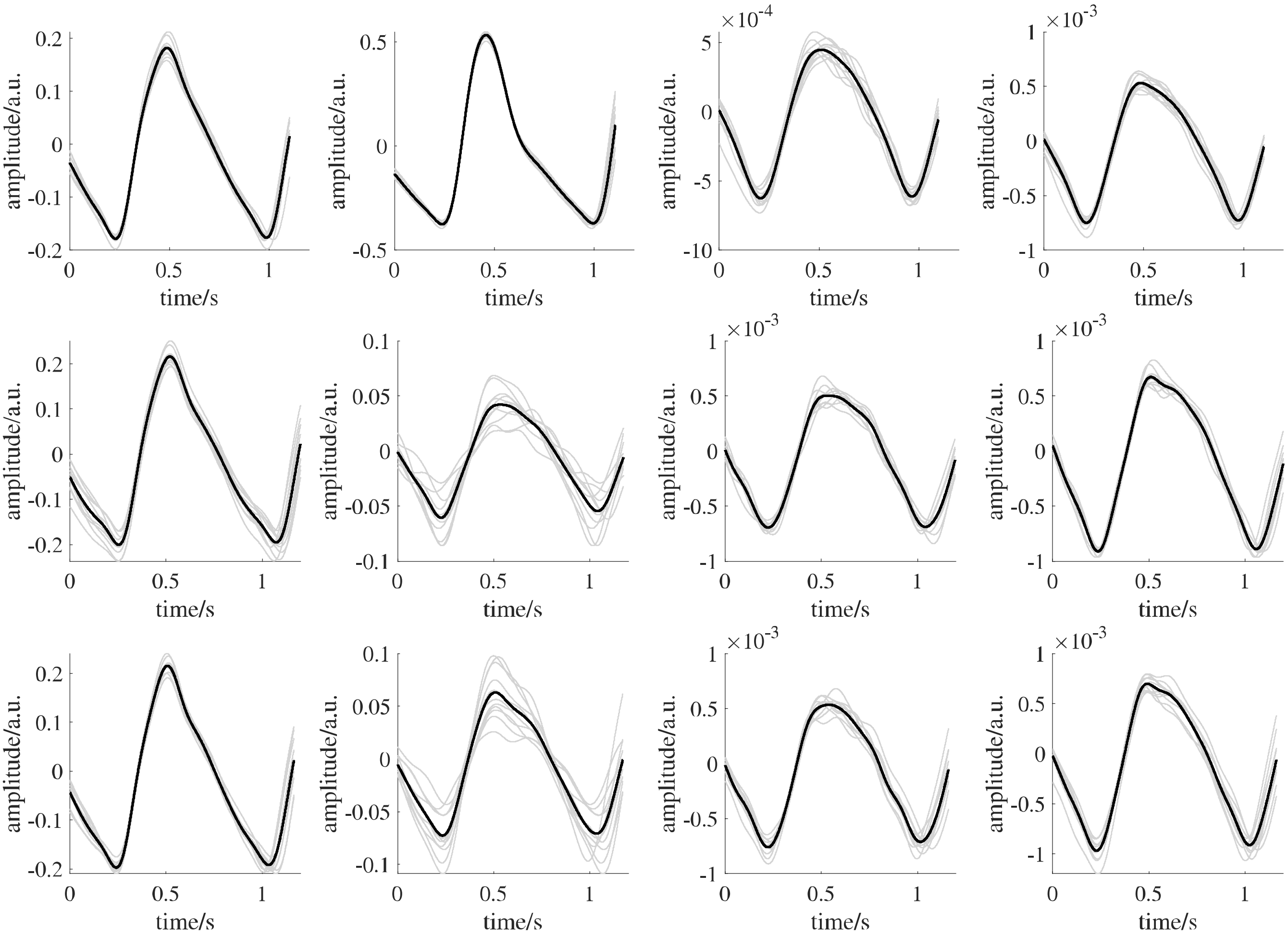

Figure S14. Template generation of one subject. From left to right: earlobe PPG (BL: 11 beats, ST1: 11 beats, ST2: 11 beats), finger PPG (BL: 11 beats, ST1: 11 beats, ST2: 11 beats), forehead iPPG (BL: 12 beats, ST1: 11 beats, ST2: 11 beats), super ROI iPPG (BL: 12 beats, ST1: 10 beats, ST2: 11 beats). The upper row shows signals during BL. The middle row shows signals during ST1. The lower row shows signals during ST2. Black lines indicate mean beat templates; gray lines indicate the corresponding beat segments.

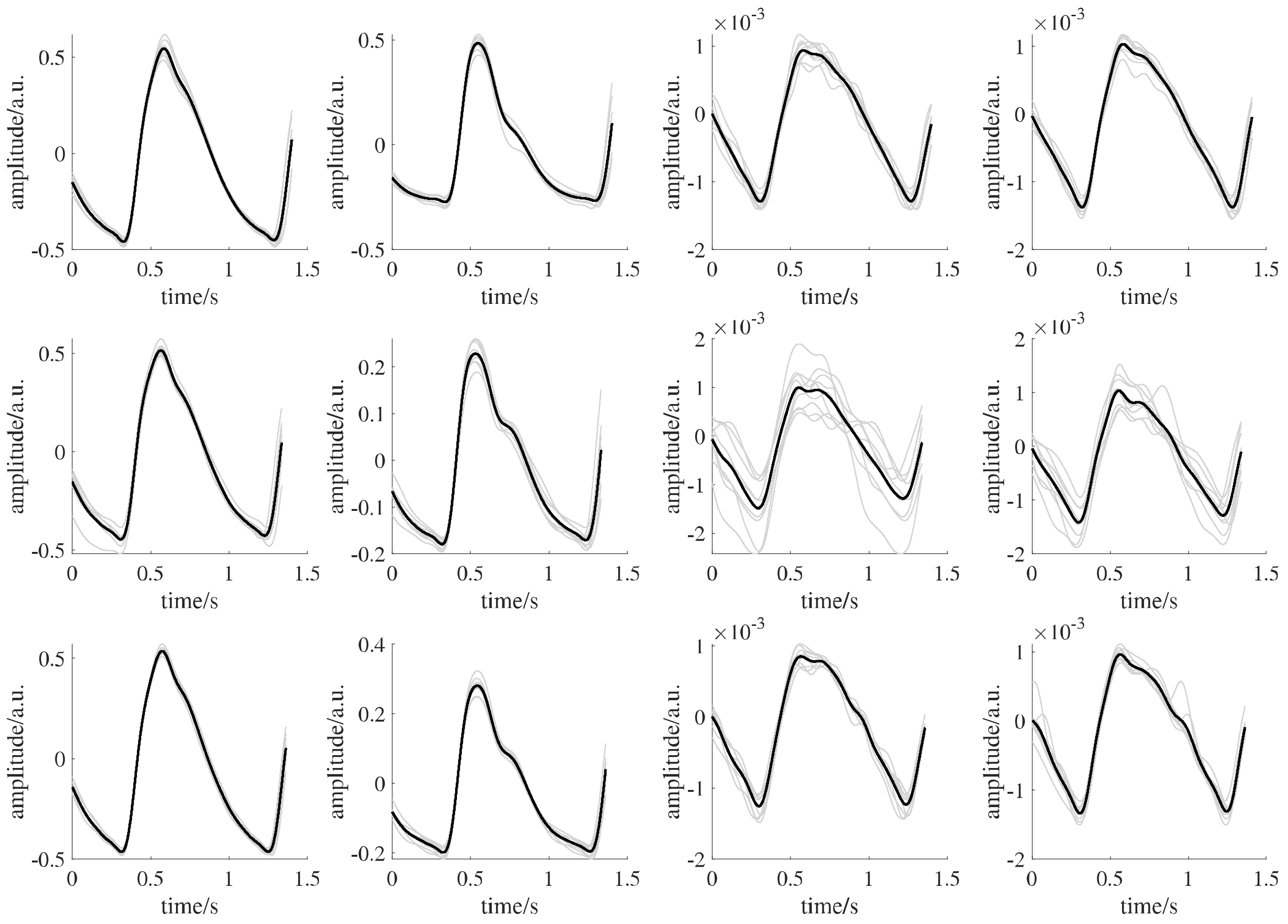

Figure S15. Template generation of one subject. From left to right: earlobe PPG (BL: 9 beats, ST1: 9 beats, ST2: 9 beats), finger PPG (BL: 9 beats, ST1: 9 beats, ST2: 9 beats), forehead iPPG (BL: 9 beats, ST1: 9 beats, ST2: 9 beats), super ROI iPPG (BL: 9 beats, ST1: 9 beats, ST2: 9 beats). The upper row shows signals during BL. The middle row shows signals during ST1. The lower row shows signals during ST2. Black lines indicate mean beat templates; gray lines indicate the corresponding beat segments.

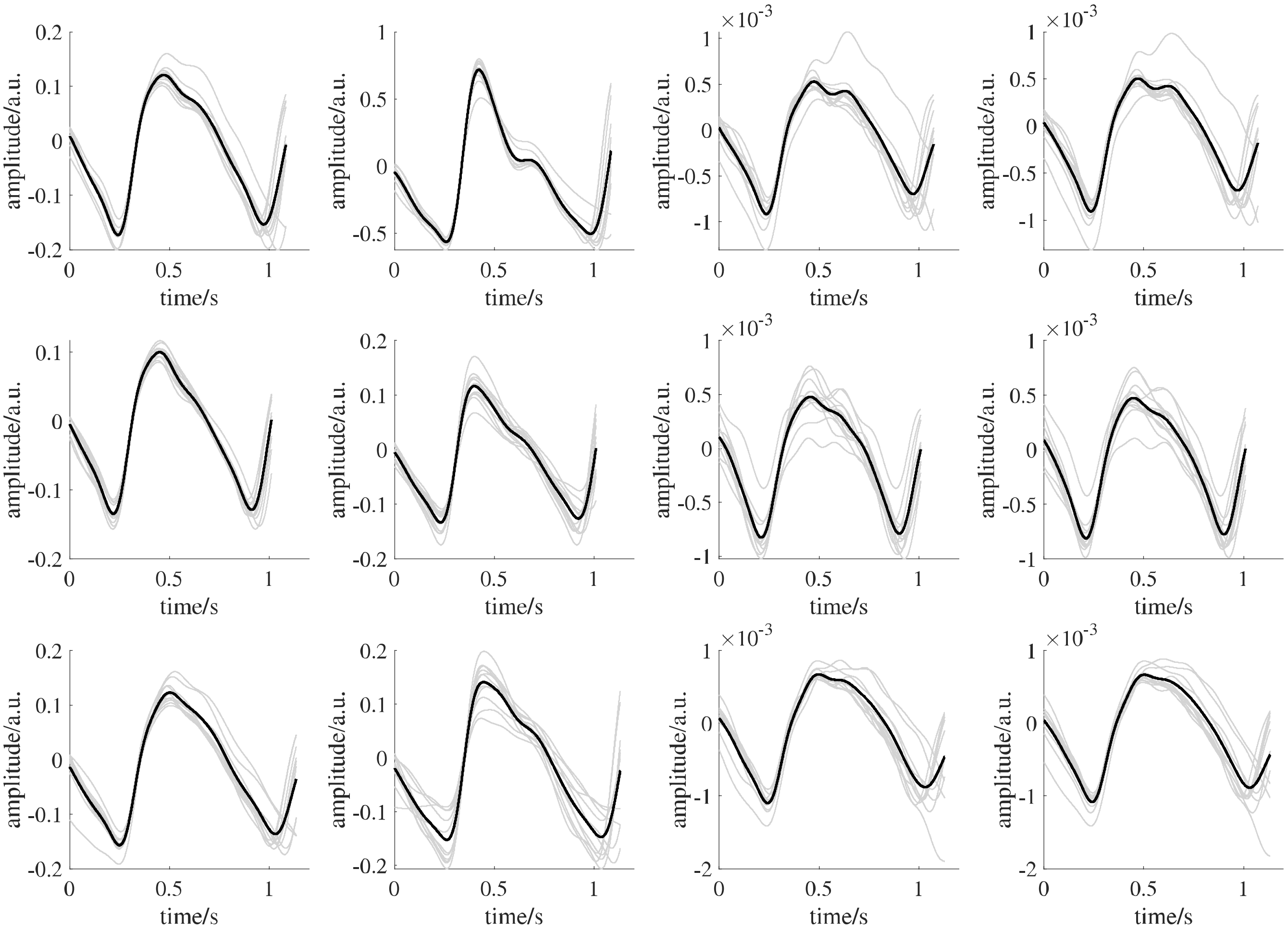

Figure S16. Template generation of one subject. From left to right: earlobe PPG (BL: 11 beats, ST1: 13 beats, ST2: 11 beats), finger PPG (BL: 11 beats, ST1: 13 beats, ST2: 11 beats), forehead iPPG (BL: 12 beats, ST1: 13 beats, ST2: 11 beats), super ROI iPPG (BL: 12 beats, ST1: 13 beats, ST2: 11 beats). The upper row shows signals during BL. The middle row shows signals during ST1. The lower row shows signals during ST2. Black lines indicate mean beat templates; gray lines indicate the corresponding beat segments.

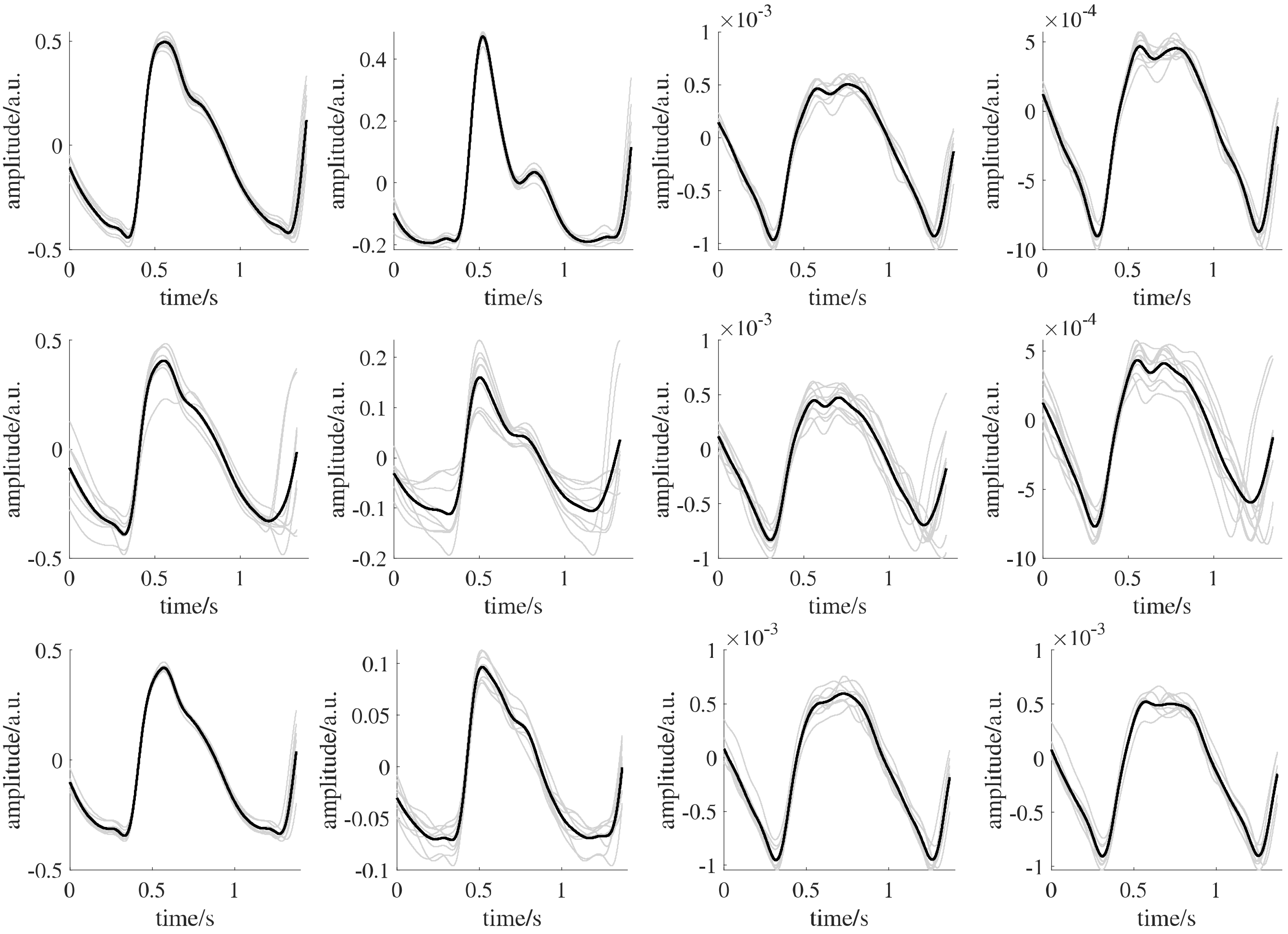

Figure S17. Template generation of one subject. From left to right: earlobe PPG (BL: 9 beats, ST1: 9 beats, ST2: 9 beats), finger PPG (BL: 9 beats, ST1: 9 beats, ST2: 9 beats), forehead iPPG (BL: 9 beats, ST1: 10 beats, ST2: 9 beats), super ROI iPPG (BL: 9 beats, ST1: 10 beats, ST2: 9 beats). The upper row shows signals during BL. The middle row shows signals during ST1. The lower row shows signals during ST2. Black lines indicate mean beat templates; gray lines indicate the corresponding beat segments.

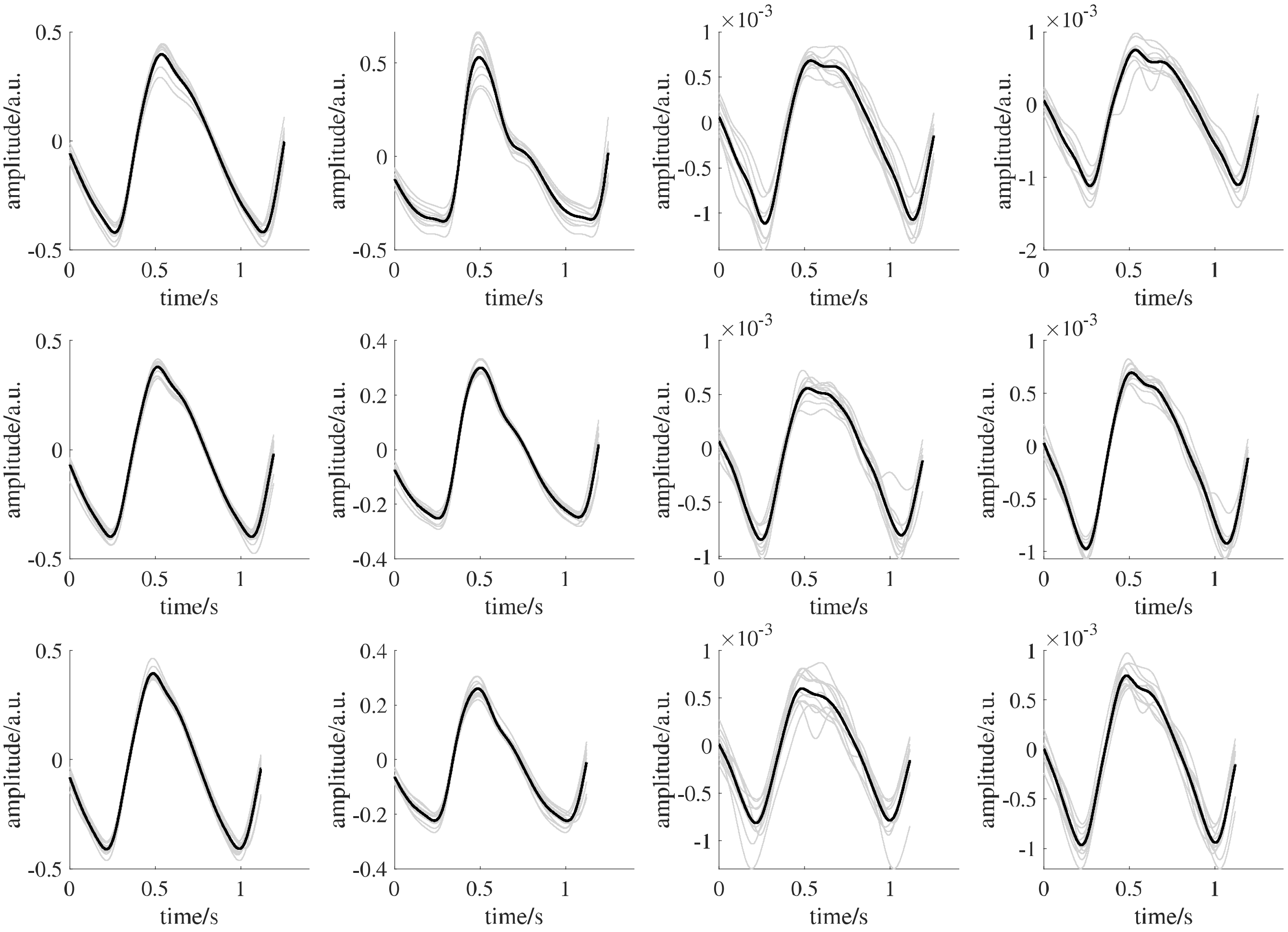

Figure S18. Template generation of one subject. From left to right: earlobe PPG (BL: 10 beats, ST1: 11 beats, ST2: 11 beats), finger PPG (BL: 10 beats, ST1: 11 beats, ST2: 12 beats), forehead iPPG (BL: 10 beats, ST1: 11 beats, ST2: 11 beats), super ROI iPPG (BL: 10 beats, ST1: 10 beats, ST2: 11 beats). The upper row shows signals during BL. The middle row shows signals during ST1. The lower row shows signals during ST2. Black lines indicate mean beat templates; gray lines indicate the corresponding beat segments.

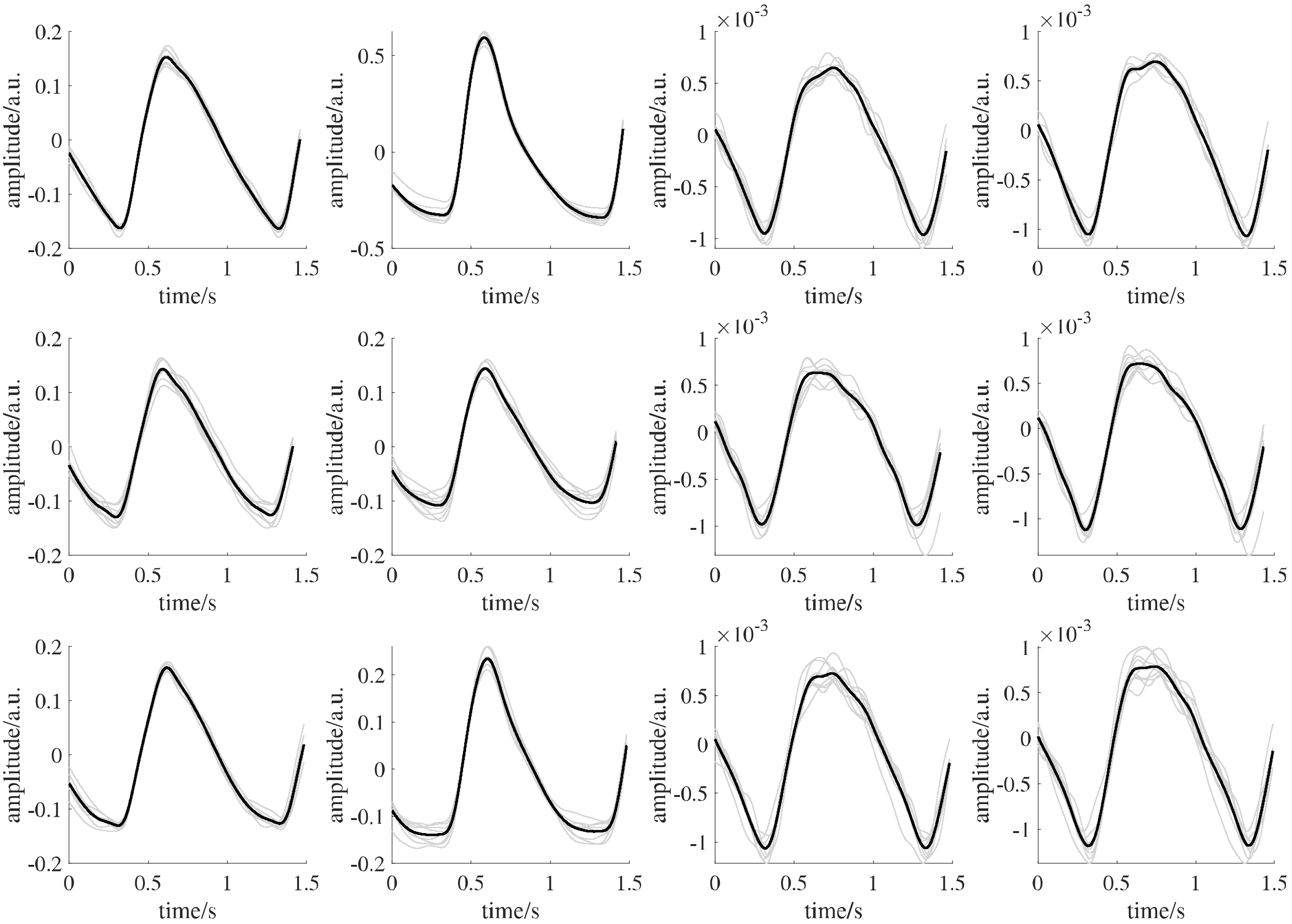

Figure S19. Template generation of one subject. From left to right: earlobe PPG (BL: 9 beats, ST1: 9 beats, ST2: 8 beats), finger PPG (BL: 9 beats, ST1: 9 beats, ST2: 8 beats), forehead iPPG (BL: 8 beats, ST1: 9 beats, ST2: 9 beats), super ROI iPPG (BL: 8 beats, ST1: 9 beats, ST2: 9 beats). The upper row shows signals during BL. The middle row shows signals during ST1. The lower row shows signals during ST2. Black lines indicate mean beat templates; gray lines indicate the corresponding beat segments.

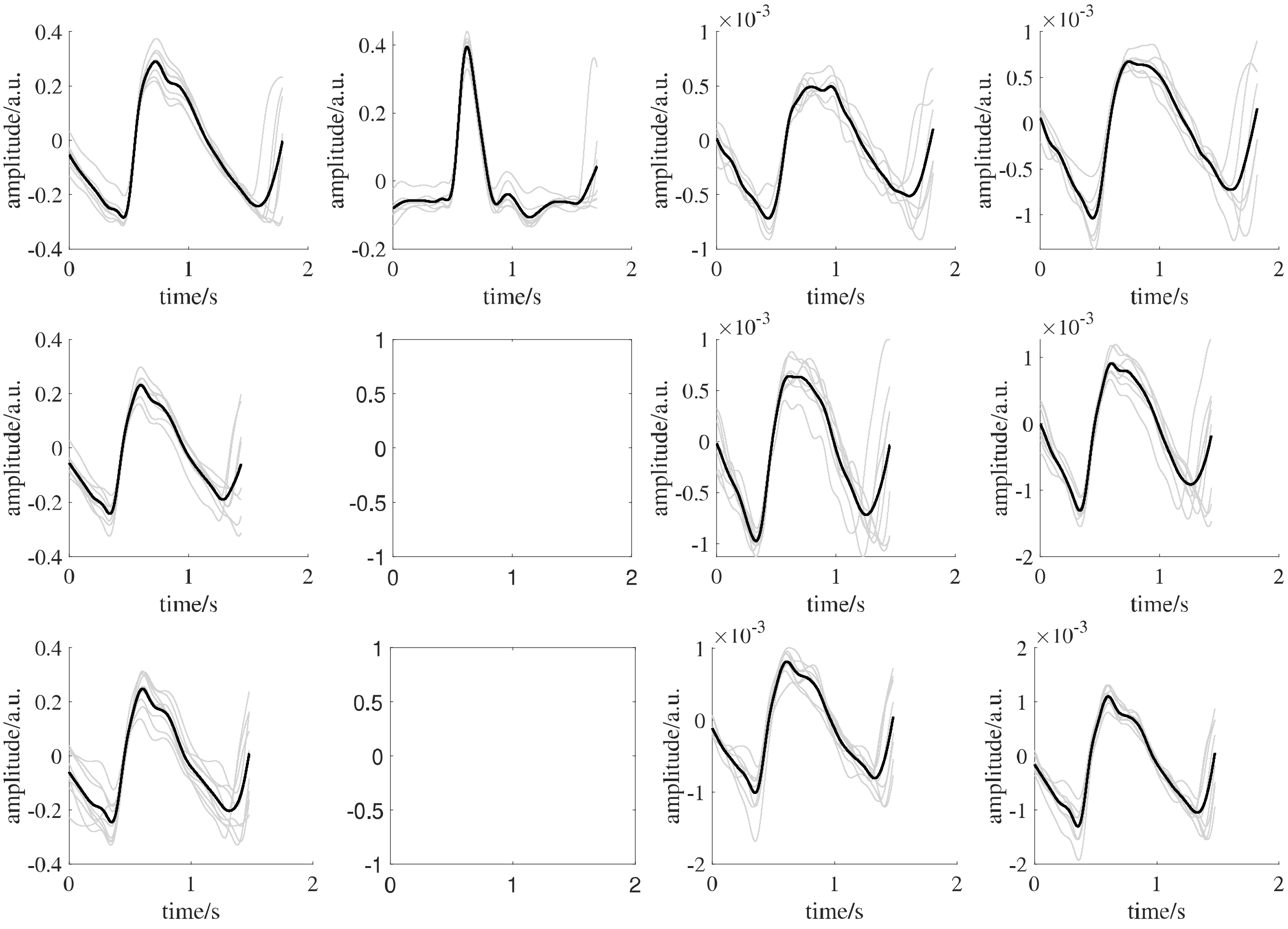

Figure S20. Template generation of one subject. From left to right: earlobe PPG (BL: 7 beats, ST1: 8 beats, ST2: 9 beats), finger PPG (BL: 7 beats, ST1: 4 beats, ST2: 0 beats), forehead iPPG (BL: 6 beats, ST1: 8 beats, ST2: 8 beats), super ROI iPPG (BL: 6 beats, ST1: 8 beats, ST2: 8 beats). The upper row shows signals during BL. The middle row shows signals during ST1. The lower row shows signals during ST2. Black lines indicate mean beat templates; gray lines indicate the corresponding beat segments.

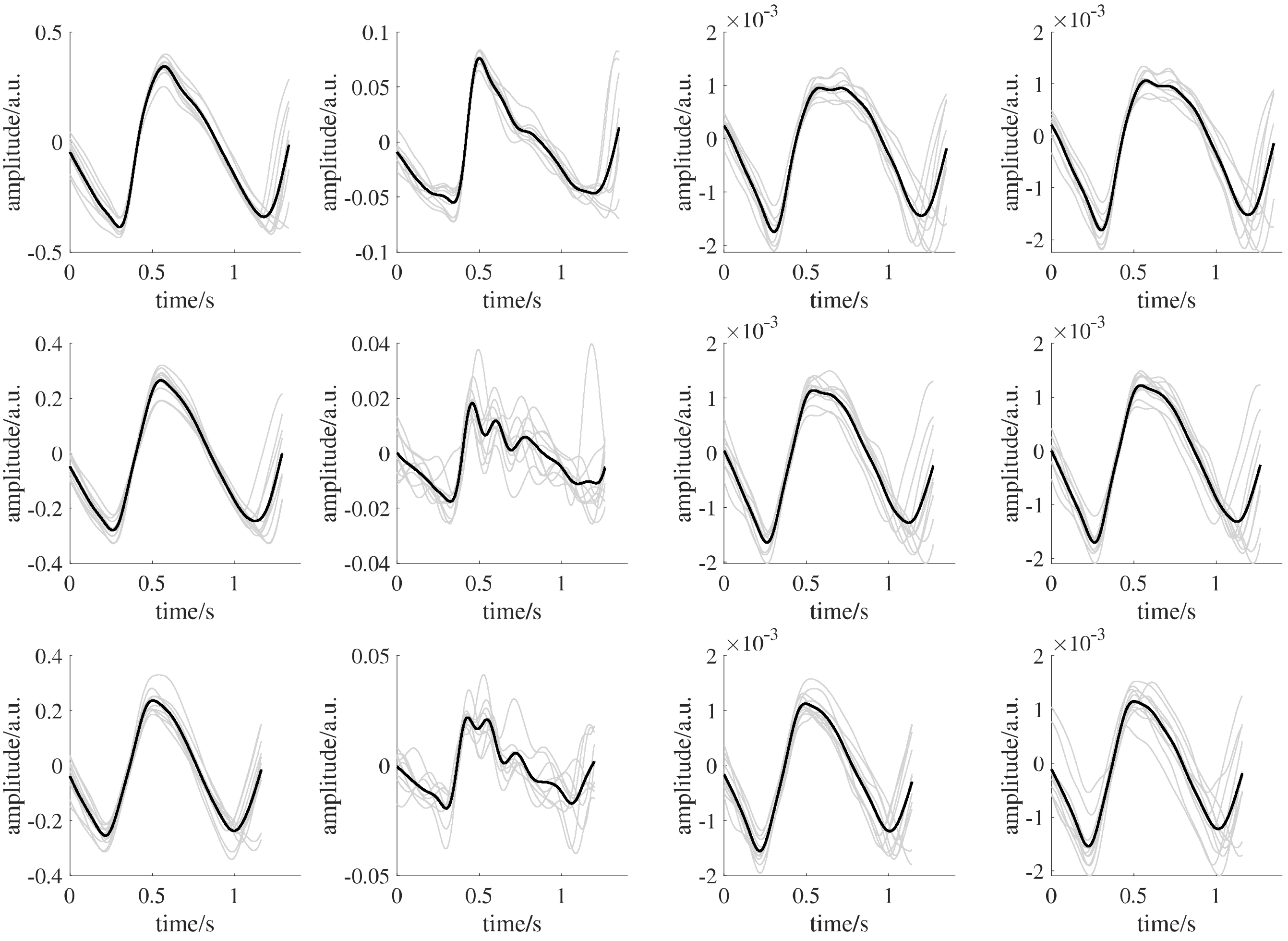

Figure S21. Template generation of one subject. From left to right: earlobe PPG (BL: 9 beats, ST1: 10 beats, ST2: 11 beats), finger PPG (BL: 9 beats, ST1: 10 beats, ST2: 9 beats), forehead iPPG (BL: 9 beats, ST1: 10 beats, ST2: 11 beats), super ROI iPPG (BL: 9 beats, ST1: 10 beats, ST2: 11 beats). The upper row shows signals during BL. The middle row shows signals during ST1. The lower row shows signals during ST2. Black lines indicate mean beat templates; gray lines indicate the corresponding beat segments.

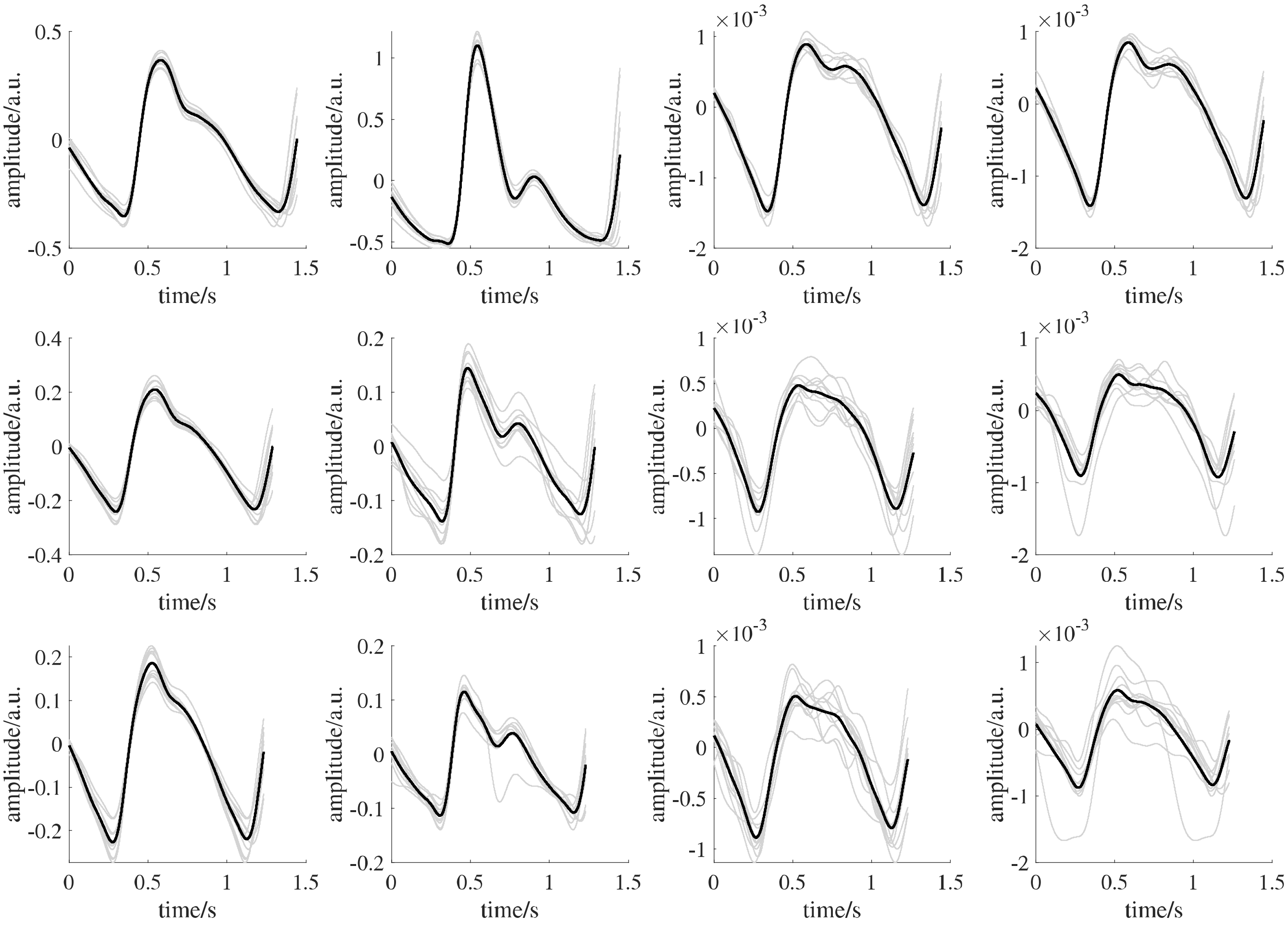

Figure S22. Template generation of one subject. From left to right: earlobe PPG (BL: 9 beats, ST1: 10 beats, ST2: 10 beats), finger PPG (BL: 9 beats, ST1: 10 beats, ST2: 10 beats), forehead iPPG (BL: 9 beats, ST1: 10 beats, ST2: 11 beats), super ROI iPPG (BL: 9 beats, ST1: 10 beats, ST2: 11 beats). The upper row shows signals during BL. The middle row shows signals during ST1. The lower row shows signals during ST2. Black lines indicate mean beat templates; gray lines indicate the corresponding beat segments.

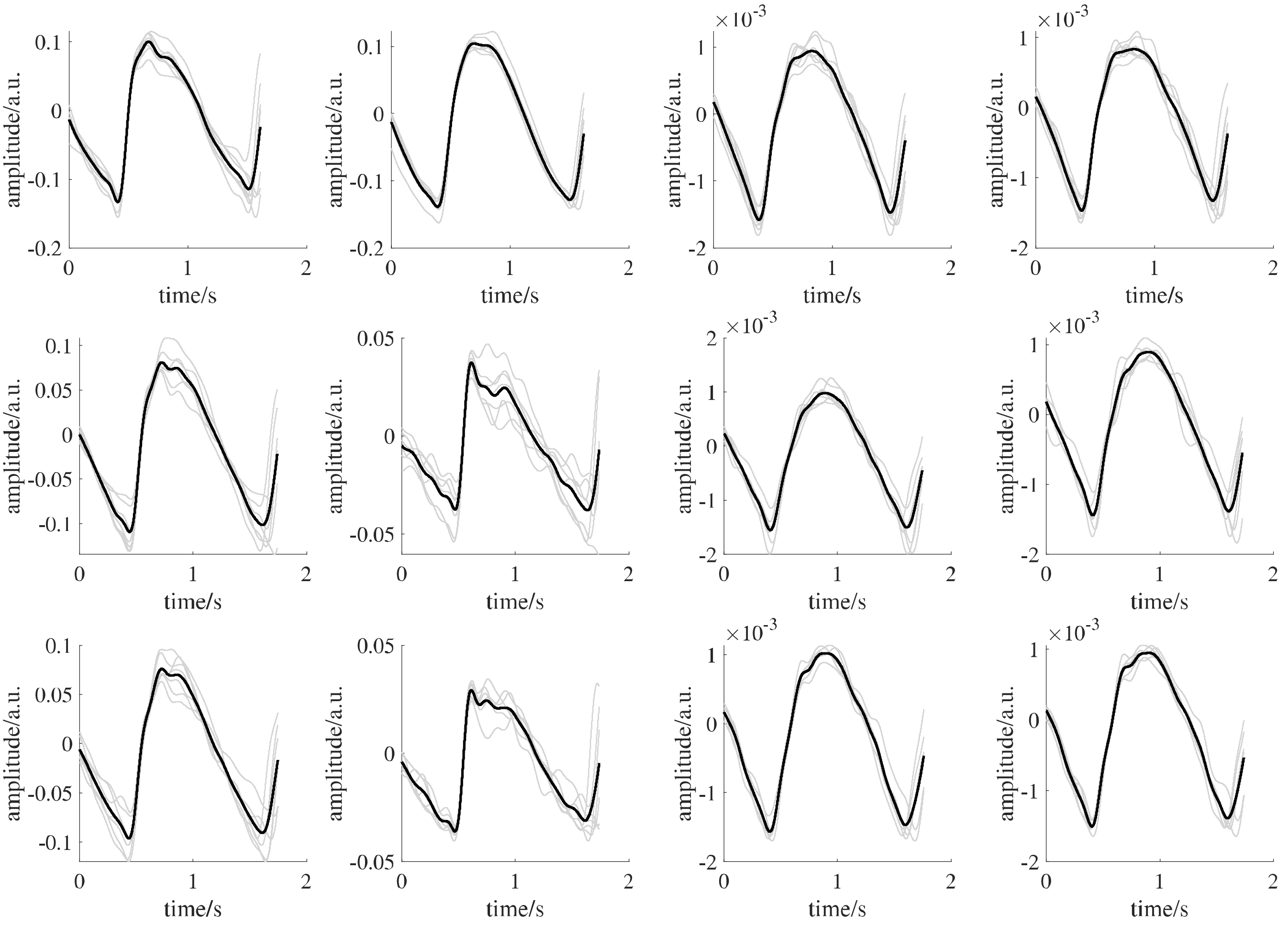

Figure S23. Template generation of one subject. From left to right: earlobe PPG (BL: 8 beats, ST1: 7 beats, ST2: 7 beats), finger PPG (BL: 7 beats, ST1: 7 beats, ST2: 7 beats), forehead iPPG (BL: 8 beats, ST1: 7 beats, ST2: 6 beats), super ROI iPPG (BL: 8 beats, ST1: 7 beats, ST2: 6 beats). The upper row shows signals during BL. The middle row shows signals during ST1. The lower row shows signals during ST2. Black lines indicate mean beat templates; gray lines indicate the corresponding beat segments.

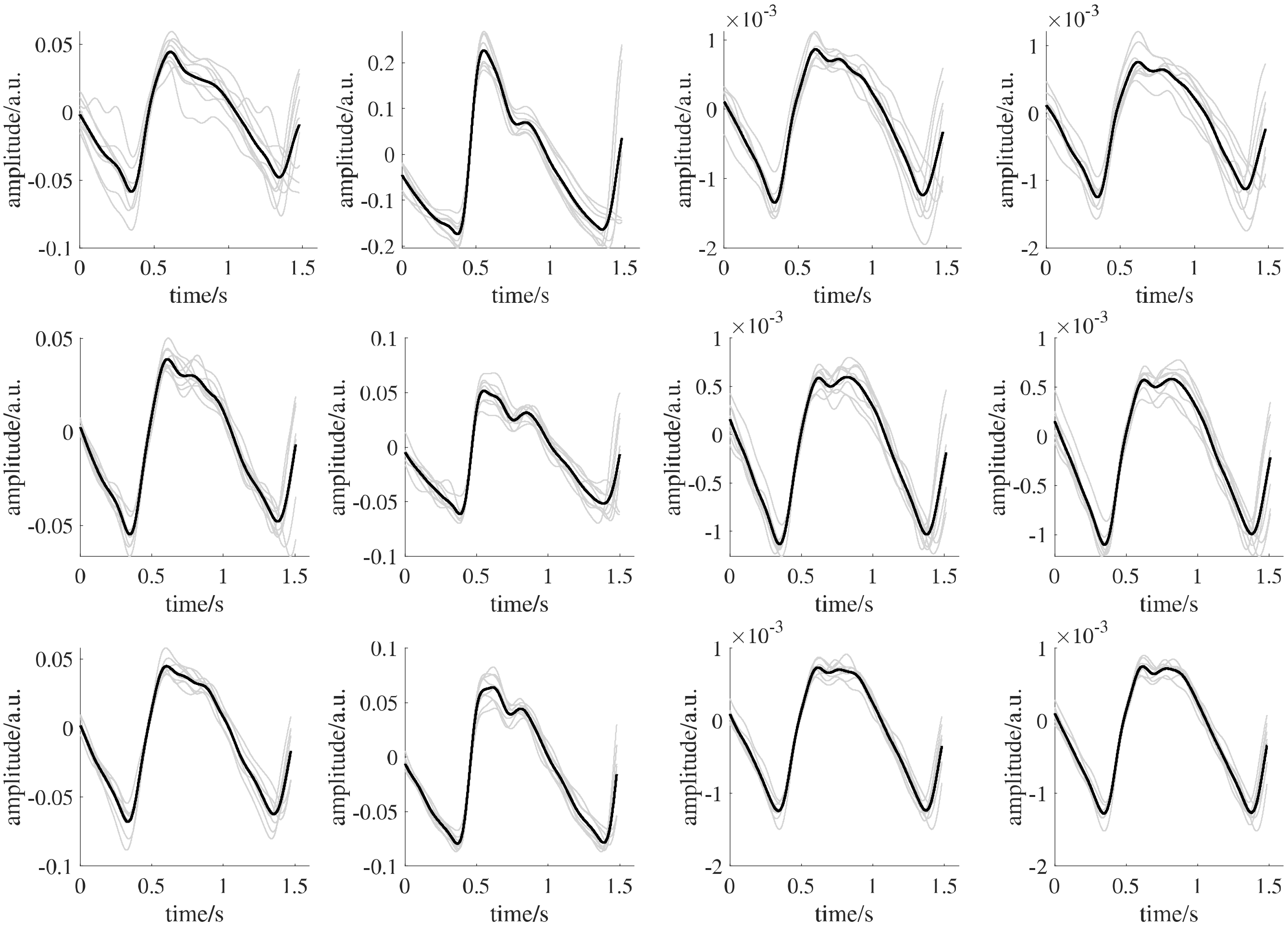

Figure S24. Template generation of one subject. From left to right: earlobe PPG (BL: 9 beats, ST1: 9 beats, ST2: 8 beats), finger PPG (BL: 8 beats, ST1: 9 beats, ST2: 8 beats), forehead iPPG (BL: 8 beats, ST1: 8 beats, ST2: 8 beats), super ROI iPPG (BL: 8 beats, ST1: 8 beats, ST2: 8 beats). The upper row shows signals during BL. The middle row shows signals during ST1. The lower row shows signals during ST2. Black lines indicate mean beat templates; gray lines indicate the corresponding beat segments.

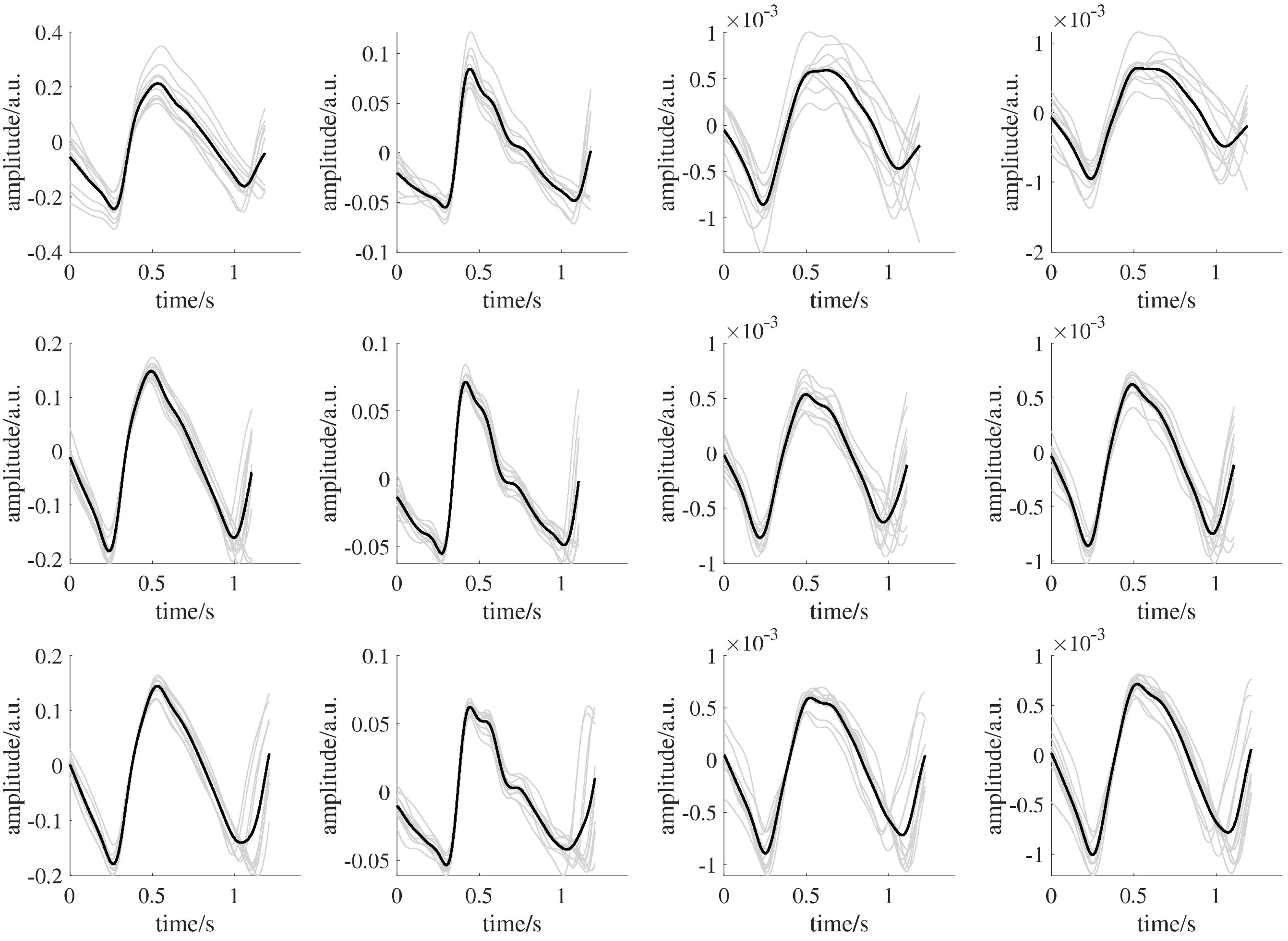

Figure S25. Template generation of one subject. From left to right: earlobe PPG (BL: 9 beats, ST1: 11 beats, ST2: 10 beats), finger PPG (BL: 9 beats, ST1: 11 beats, ST2: 11 beats), forehead iPPG (BL: 10 beats, ST1: 12 beats, ST2: 11 beats), super ROI iPPG (BL: 10 beats, ST1: 12 beats, ST2: 11 beats). The upper row shows signals during BL. The middle row shows signals during ST1. The lower row shows signals during ST2. Black lines indicate mean beat templates; gray lines indicate the corresponding beat segments.

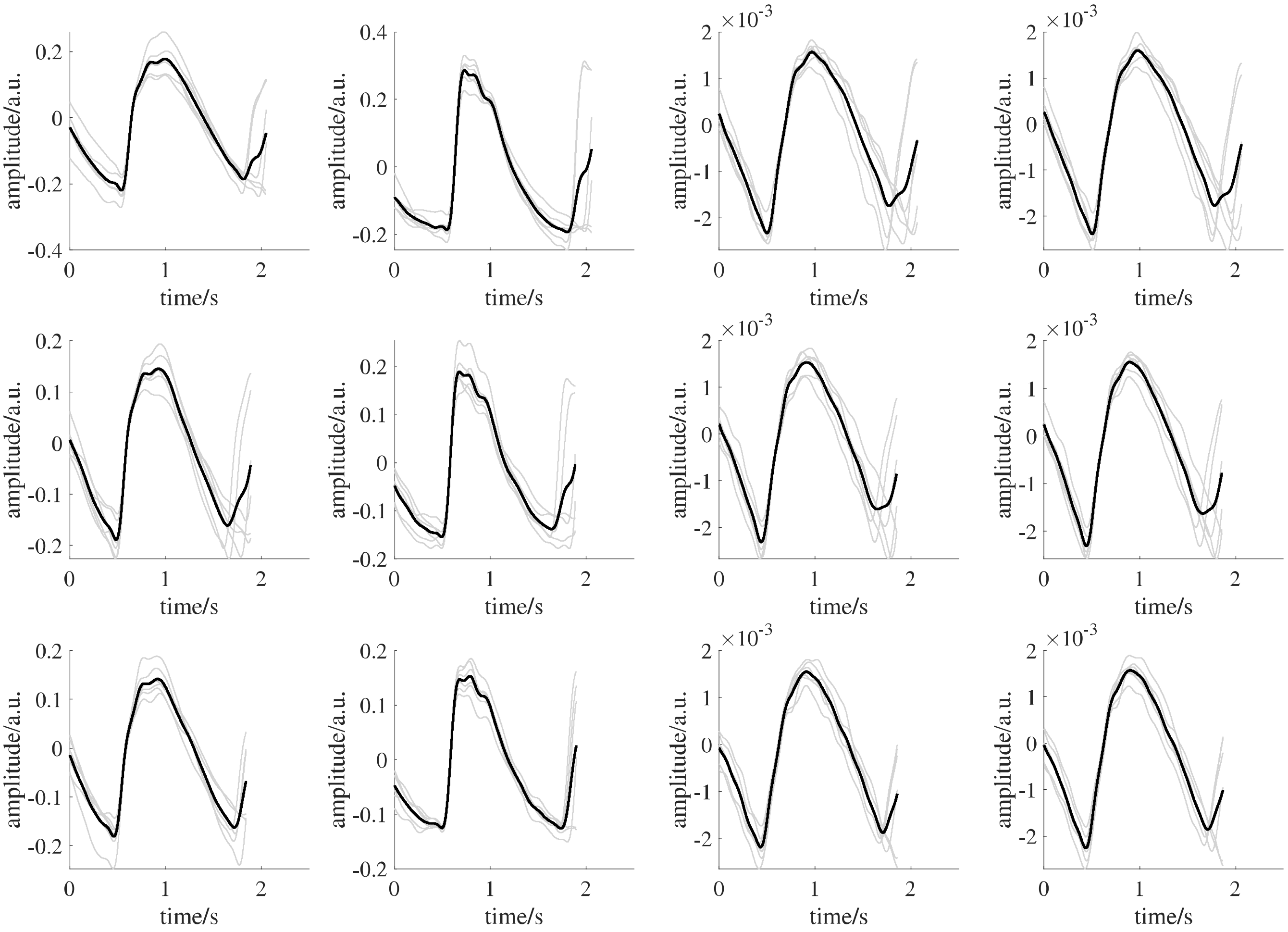

Figure S26. Template generation of one subject. From left to right: earlobe PPG (BL: 6 beats, ST1: 6 beats, ST2: 6 beats), finger PPG (BL: 6 beats, ST1: 6 beats, ST2: 6 beats), forehead iPPG (BL: 6 beats, ST1: 6 beats, ST2: 6 beats), super ROI iPPG (BL: 6 beats, ST1: 6 beats, ST2: 6 beats). The upper row shows signals during BL. The middle row shows signals during ST1. The lower row shows signals during ST2. Black lines indicate mean beat templates; gray lines indicate the corresponding beat segments.

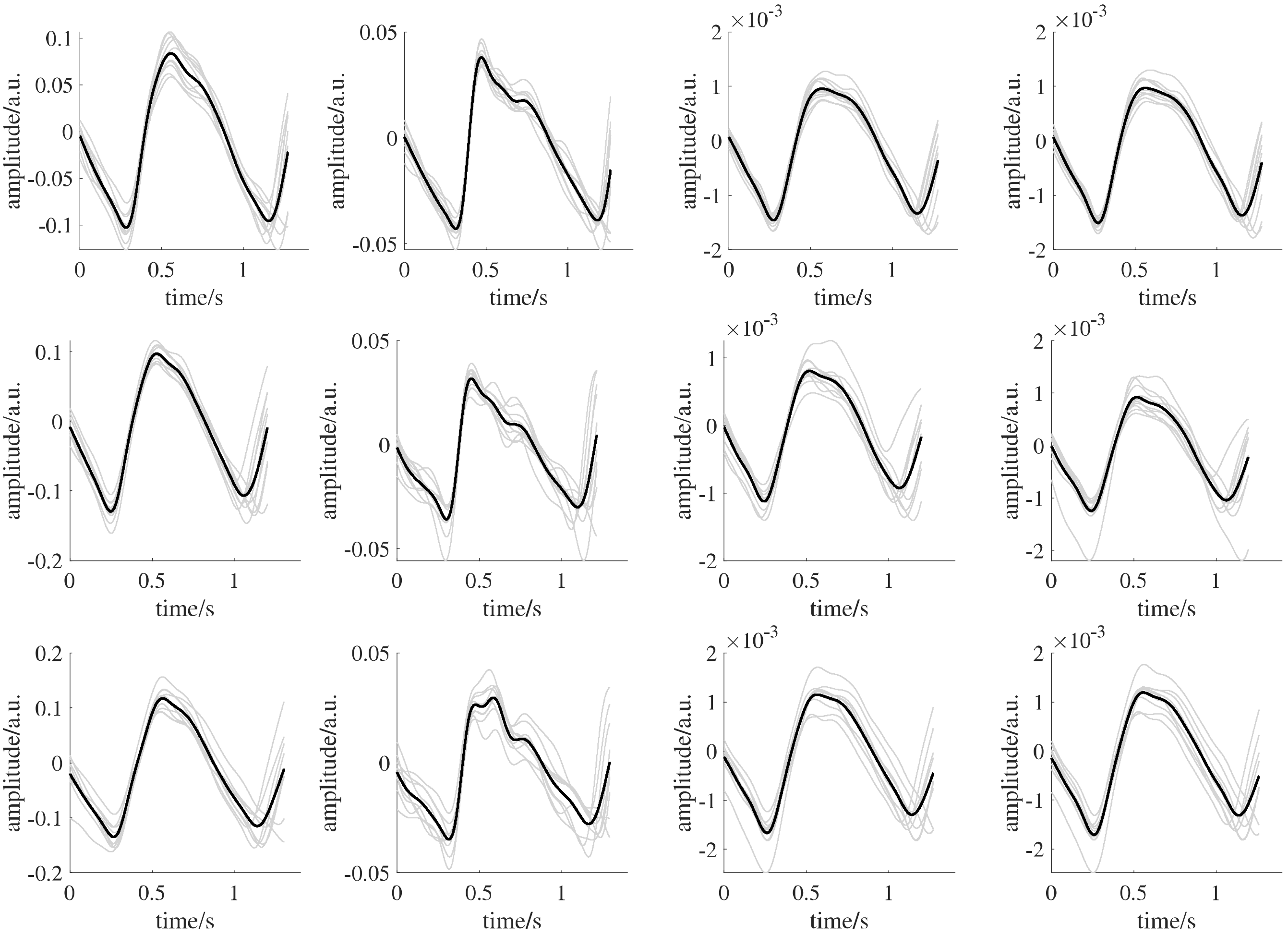

Figure S27. Template generation of one subject. From left to right: earlobe PPG (BL: 10 beats, ST1: 10 beats, ST2: 9 beats), finger PPG (BL: 10 beats, ST1: 10 beats, ST2: 9 beats), forehead iPPG (BL: 10 beats, ST1: 10 beats, ST2: 9 beats), super ROI iPPG (BL: 10 beats, ST1: 10 beats, ST2: 9 beats). The upper row shows signals during BL. The middle row shows signals during ST1. The lower row shows signals during ST2. Black lines indicate mean beat templates; gray lines indicate the corresponding beat segments.

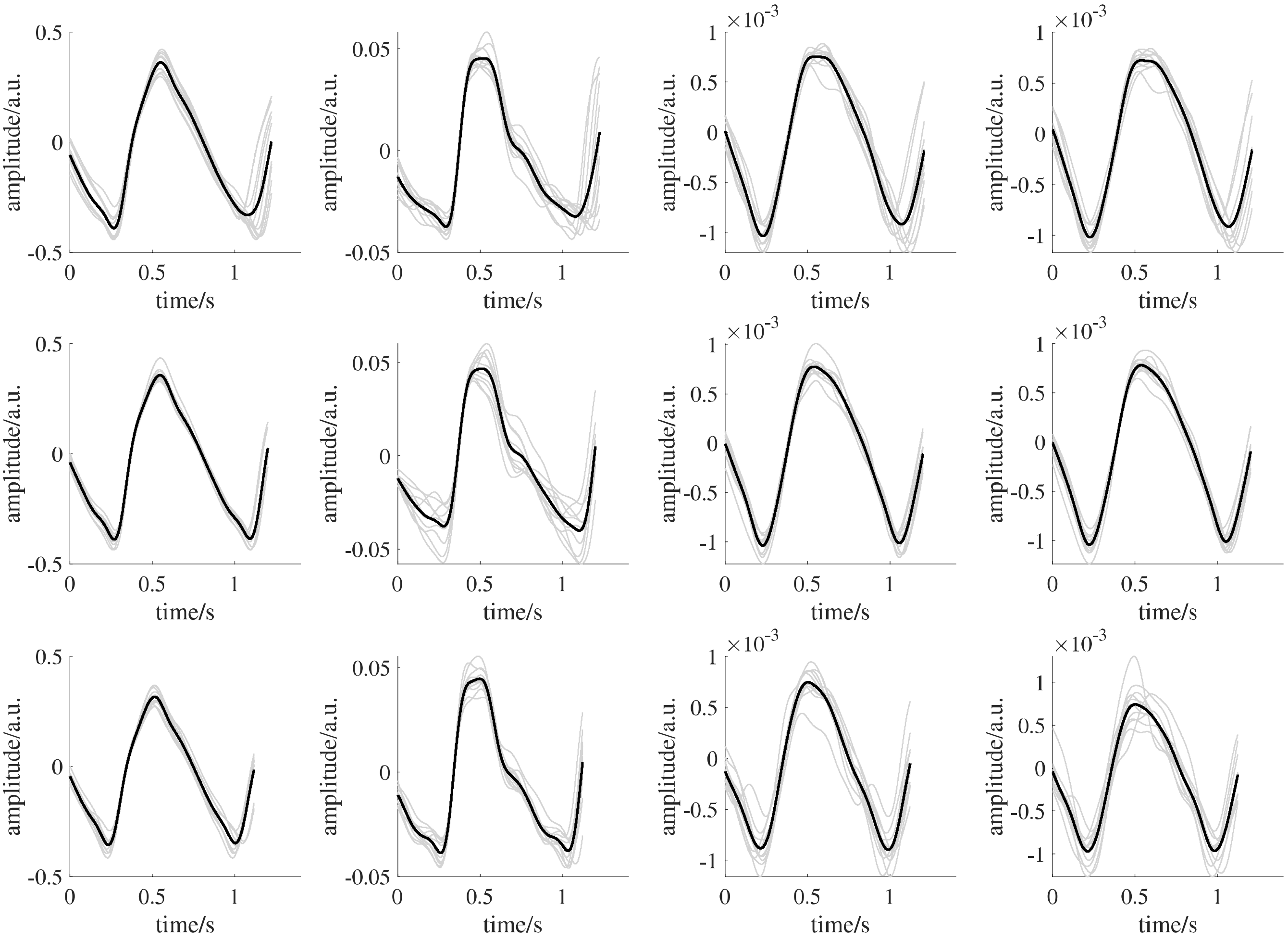

Figure S28. Template generation of one subject. From left to right: earlobe PPG (BL: 11 beats, ST1: 10 beats, ST2: 11 beats), finger PPG (BL: 11 beats, ST1: 10 beats, ST2: 11 beats), forehead iPPG (BL: 10 beats, ST1: 11 beats, ST2: 11 beats), super ROI iPPG (BL: 10 beats, ST1: 11 beats, ST2: 11 beats). The upper row shows signals during BL. The middle row shows signals during ST1. The lower row shows signals during ST2. Black lines indicate mean beat templates; gray lines indicate the corresponding beat segments.

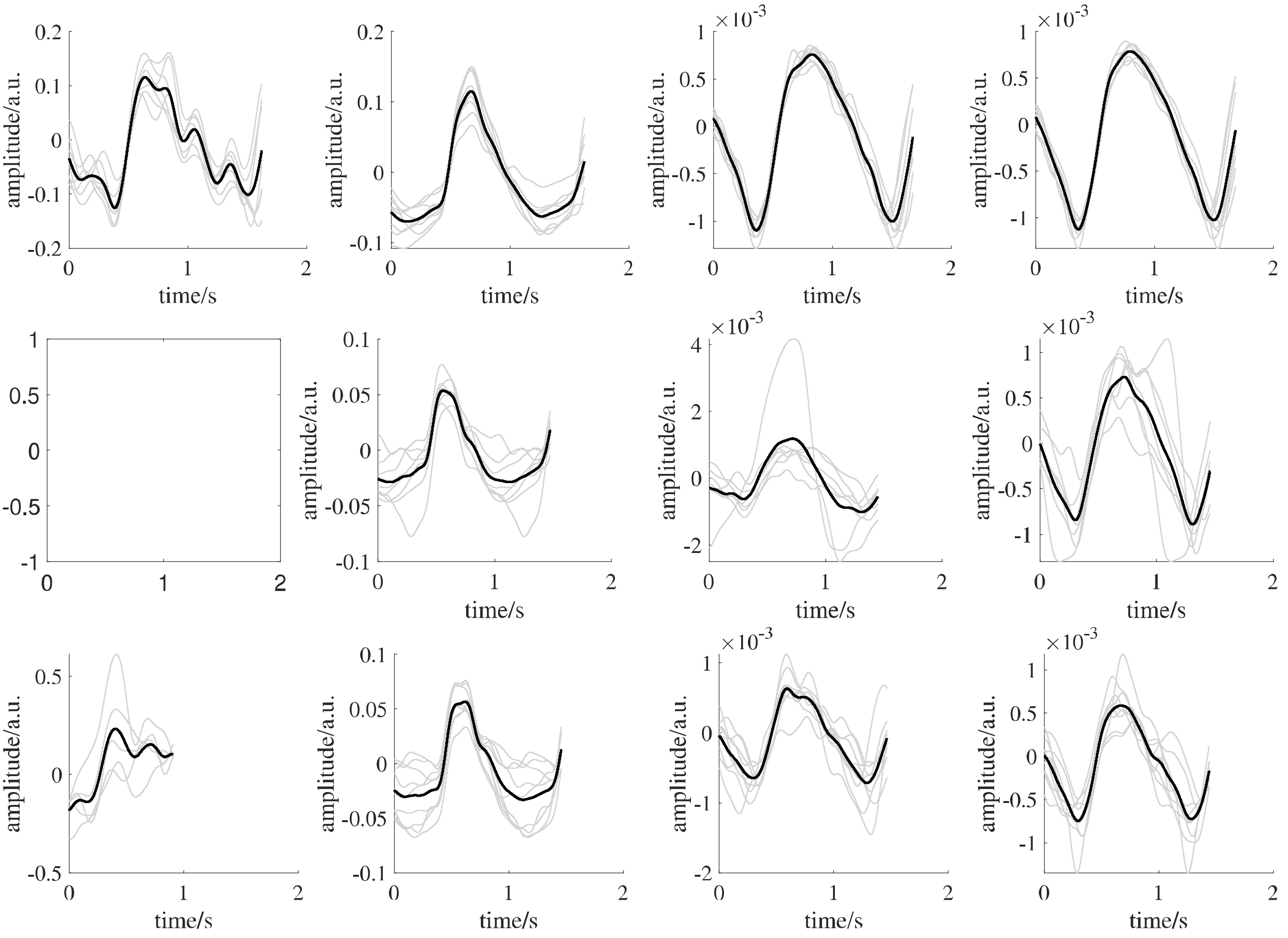

Figure S29. Template generation of one subject. From left to right: earlobe PPG (BL: 7 beats, ST1: 3 beats, ST2: 5 beats), finger PPG (BL: 8 beats, ST1: 8 beats, ST2: 9 beats), forehead iPPG (BL: 8 beats, ST1: 7 beats, ST2: 9 beats), super ROI iPPG (BL: 8 beats, ST1: 7 beats, ST2: 9 beats). The upper row shows signals during BL. The middle row shows signals during ST1. The lower row shows signals during ST2. Black lines indicate mean beat templates; gray lines indicate the corresponding beat segments.

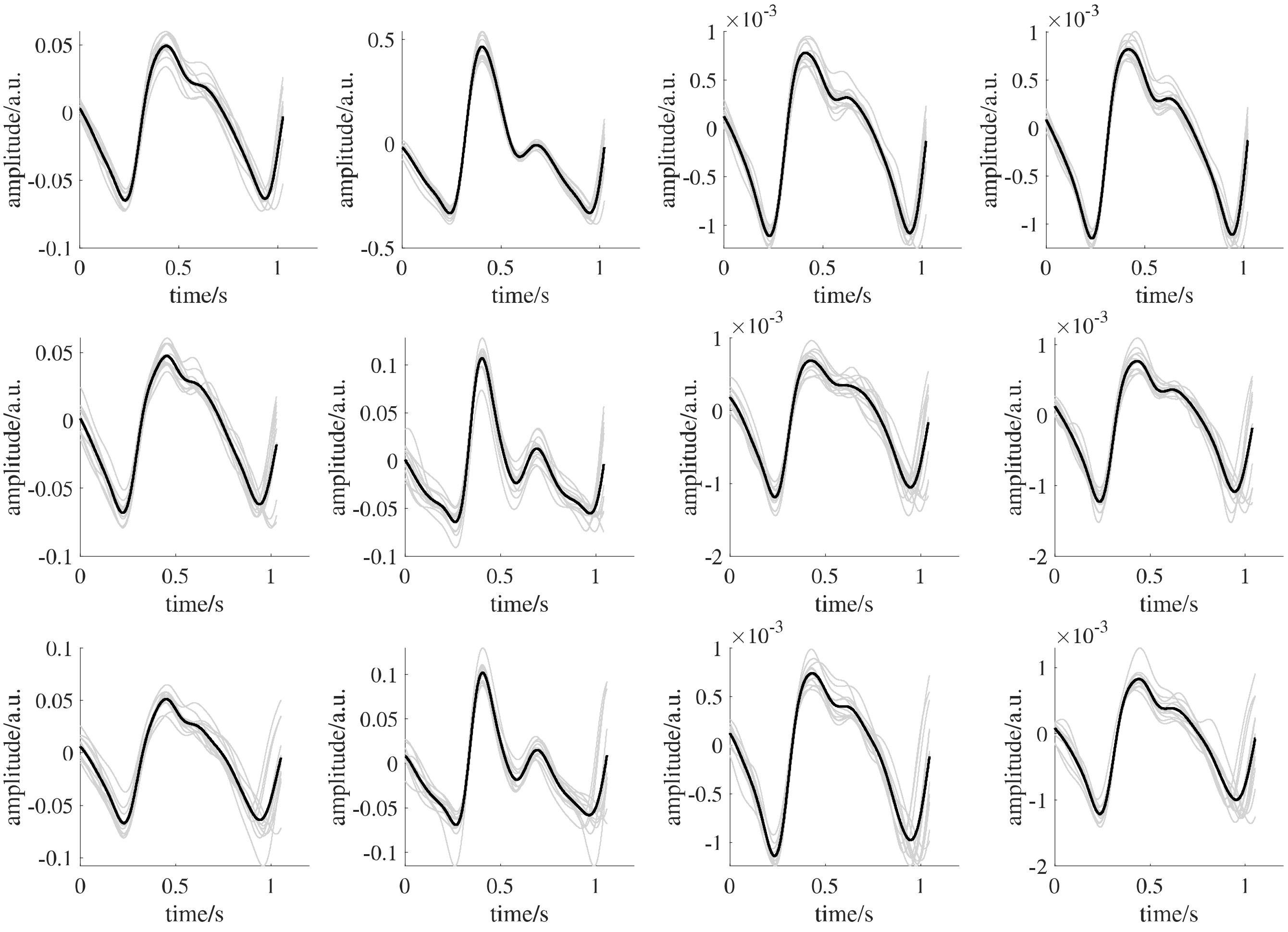

Figure S30. Template generation of one subject. From left to right: earlobe PPG (BL: 12 beats, ST1: 12 beats, ST2: 12 beats), finger PPG (BL: 12 beats, ST1: 12 beats, ST2: 12 beats), forehead iPPG (BL: 13 beats, ST1: 13 beats, ST2: 12 beats), super ROI iPPG (BL: 13 beats, ST1: 13 beats, ST2: 12 beats). The upper row shows signals during BL. The middle row shows signals during ST1. The lower row shows signals during ST2. Black lines indicate mean beat templates; gray lines indicate the corresponding beat segments.

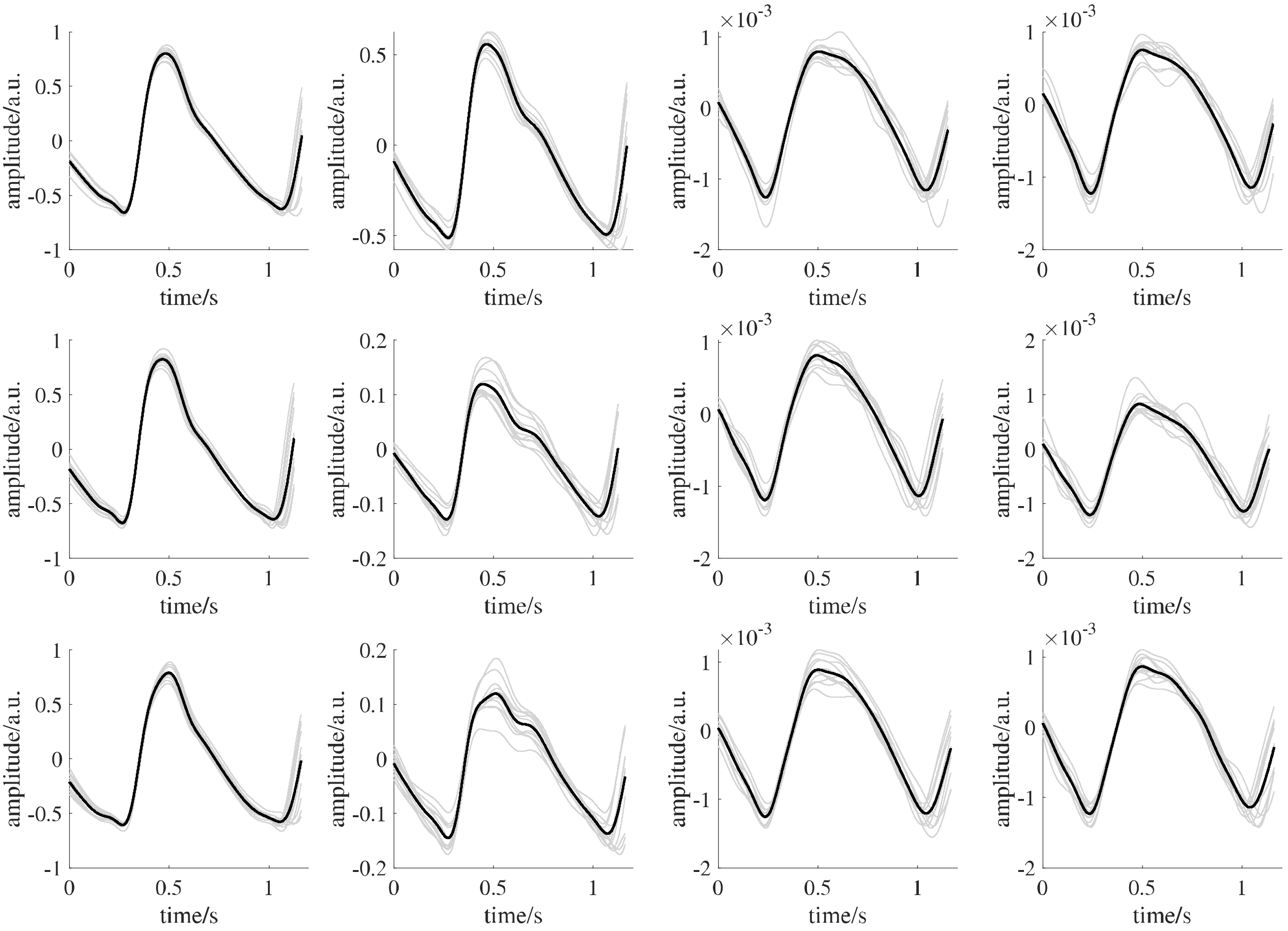

Figure S31. Template generation of one subject. From left to right: earlobe PPG (BL: 10 beats, ST1: 11 beats, ST2: 11 beats), finger PPG (BL: 11 beats, ST1: 11 beats, ST2: 11 beats), forehead iPPG (BL: 11 beats, ST1: 11 beats, ST2: 11 beats), super ROI iPPG (BL: 11 beats, ST1: 11 beats, ST2: 11 beats). The upper row shows signals during BL. The middle row shows signals during ST1. The lower row shows signals during ST2. Black lines indicate mean beat templates; gray lines indicate the corresponding beat segments.

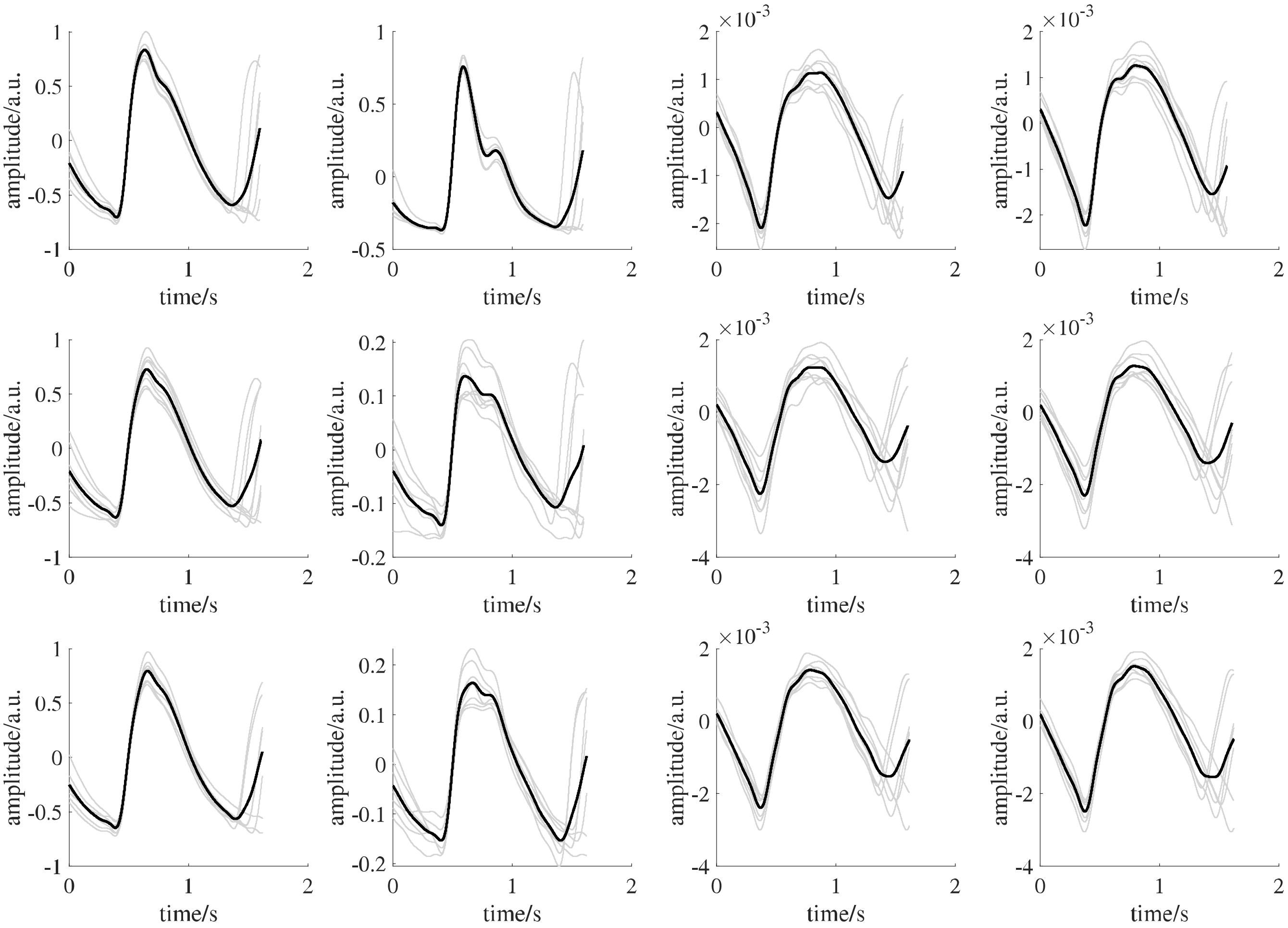

Figure S32. Template generation of one subject. From left to right: earlobe PPG (BL: 7 beats, ST1: 8 beats, ST2: 7 beats), finger PPG (BL: 7 beats, ST1: 8 beats, ST2: 7 beats), forehead iPPG (BL: 8 beats, ST1: 8 beats, ST2: 7 beats), super ROI iPPG (BL: 8 beats, ST1: 8 beats, ST2: 7 beats). The upper row shows signals during BL. The middle row shows signals during ST1. The lower row shows signals during ST2. Black lines indicate mean beat templates; gray lines indicate the corresponding beat segments.

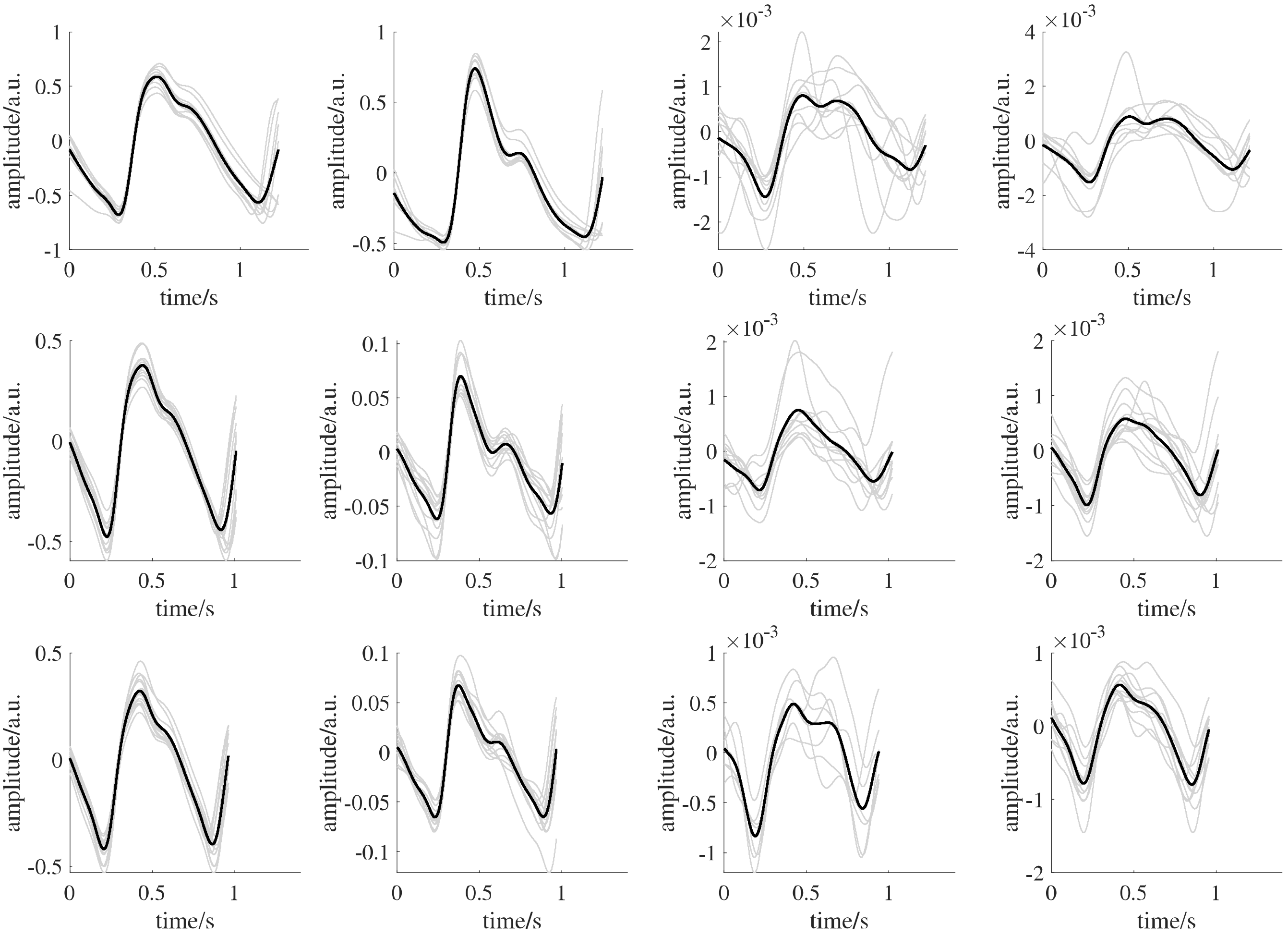

Figure S33. Template generation of one subject. From left to right: earlobe PPG (BL: 10 beats, ST1: 12 beats, ST2: 14 beats), finger PPG (BL: 9 beats, ST1: 12 beats, ST2: 14 beats), forehead iPPG (BL: 10 beats, ST1: 11 beats, ST2: 7 beats), super ROI iPPG (BL: 9 beats, ST1: 13 beats, ST2: 12 beats). The upper row shows signals during BL. The middle row shows signals during ST1. The lower row shows signals during ST2. Black lines indicate mean beat templates; gray lines indicate the corresponding beat segments.

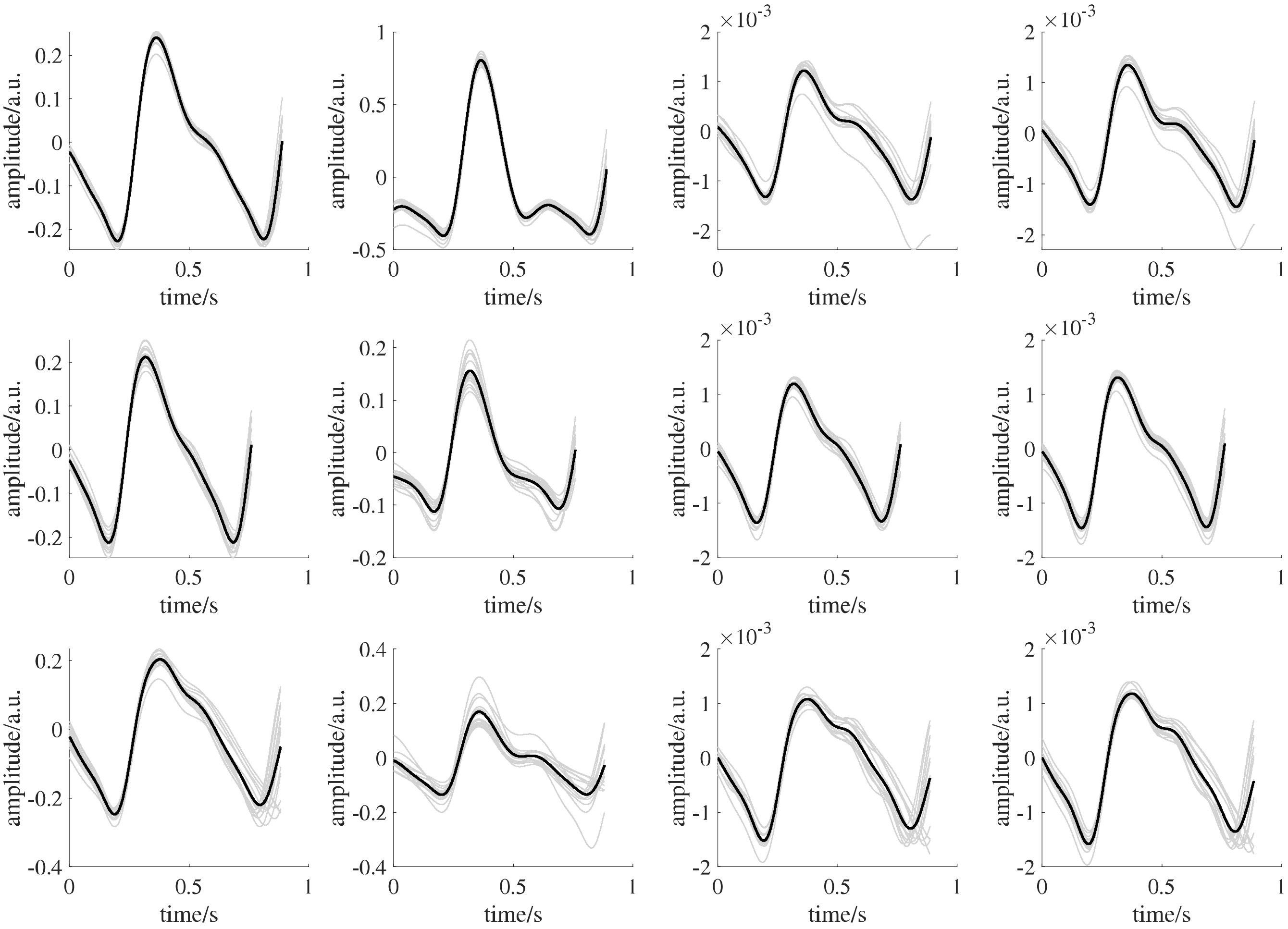

Figure S34. Template generation of one subject. From left to right: earlobe PPG (BL: 15 beats, ST1: 17 beats, ST2: 15 beats), finger PPG (BL: 15 beats, ST1: 17 beats, ST2: 15 beats), forehead iPPG (BL: 15 beats, ST1: 18 beats, ST2: 14 beats), super ROI iPPG (BL: 15 beats, ST1: 18 beats, ST2: 14 beats). The upper row shows signals during BL. The middle row shows signals during ST1. The lower row shows signals during ST2. Black lines indicate mean beat templates; gray lines indicate the corresponding beat segments.

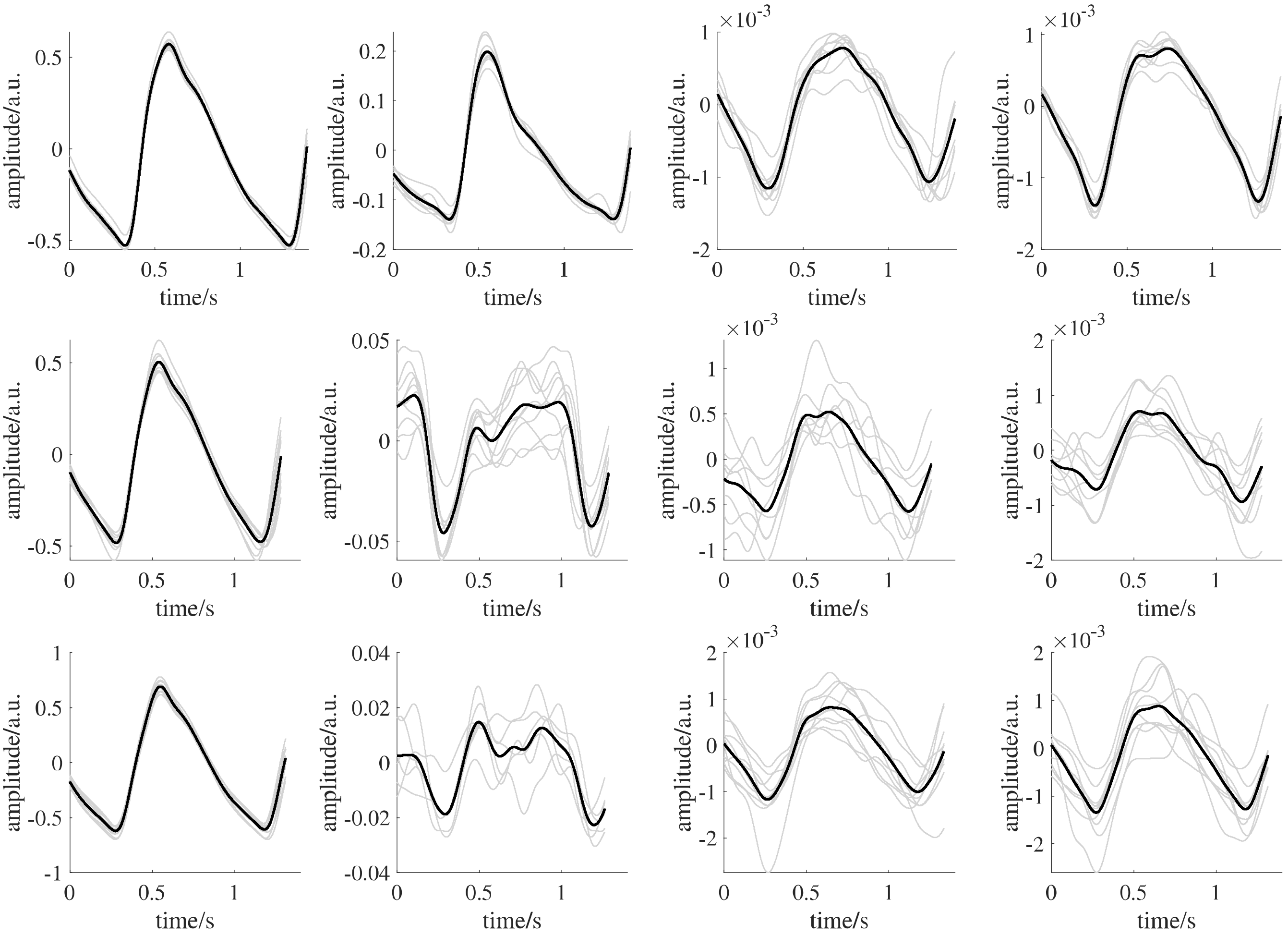

Figure S35. Template generation of one subject. From left to right: earlobe PPG (BL: 9 beats, ST1: 10 beats, ST2: 10 beats), finger PPG (BL: 9 beats, ST1: 10 beats, ST2: 6 beats), forehead iPPG (BL: 9 beats, ST1: 8 beats, ST2: 10 beats), super ROI iPPG (BL: 9 beats, ST1: 9 beats, ST2: 10 beats). The upper row shows signals during BL. The middle row shows signals during ST1. The lower row shows signals during ST2. Black lines indicate mean beat templates; gray lines indicate the corresponding beat segments.

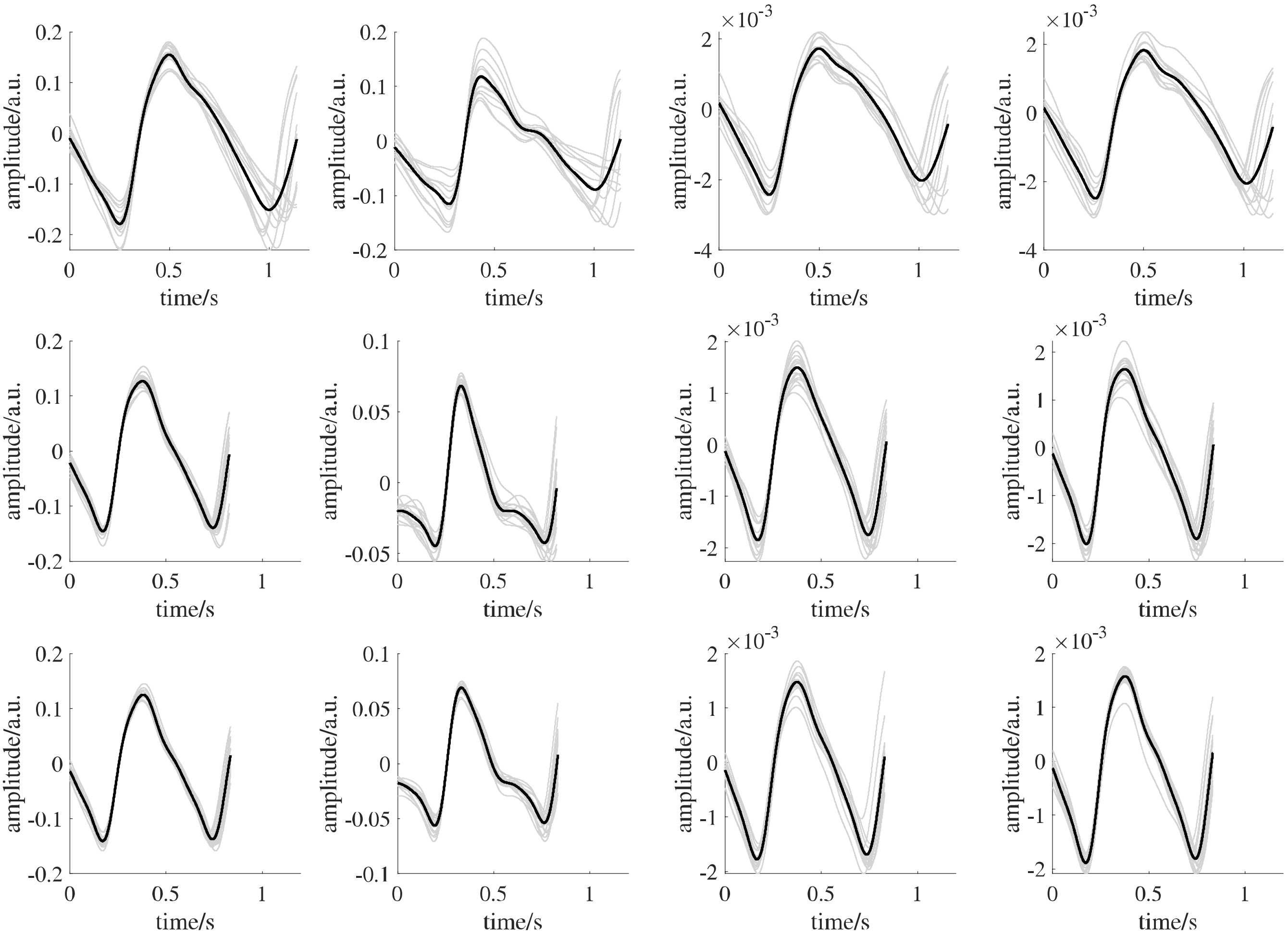

Figure S36. Template generation of one subject. From left to right: earlobe PPG (BL: 12 beats, ST1: 16 beats, ST2: 16 beats), finger PPG (BL: 12 beats, ST1: 16 beats, ST2: 16 beats), forehead iPPG (BL: 11 beats, ST1: 16 beats, ST2: 16 beats), super ROI iPPG (BL: 11 beats, ST1: 16 beats, ST2: 16 beats). The upper row shows signals during BL. The middle row shows signals during ST1. The lower row shows signals during ST2. Black lines indicate mean beat templates; gray lines indicate the corresponding beat segments.

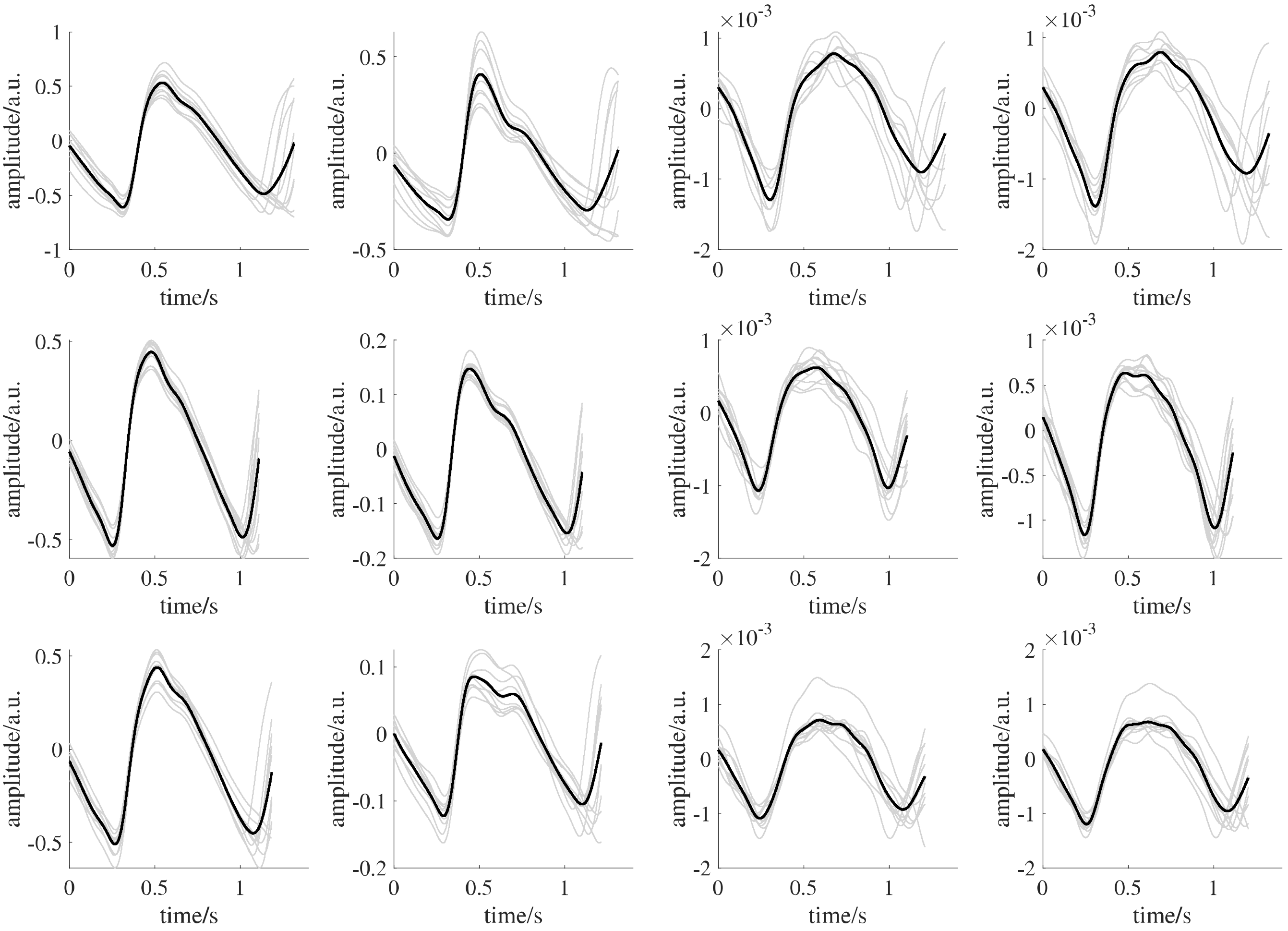

Figure S37. Template generation of one subject. From left to right: earlobe PPG (BL: 10 beats, ST1: 11 beats, ST2: 10 beats), finger PPG (BL: 10 beats, ST1: 11 beats, ST2: 10 beats), forehead iPPG (BL: 9 beats, ST1: 12 beats, ST2: 11 beats), super ROI iPPG (BL: 9 beats, ST1: 12 beats, ST2: 11 beats). The upper row shows signals during BL. The middle row shows signals during ST1. The lower row shows signals during ST2. Black lines indicate mean beat templates; gray lines indicate the corresponding beat segments.

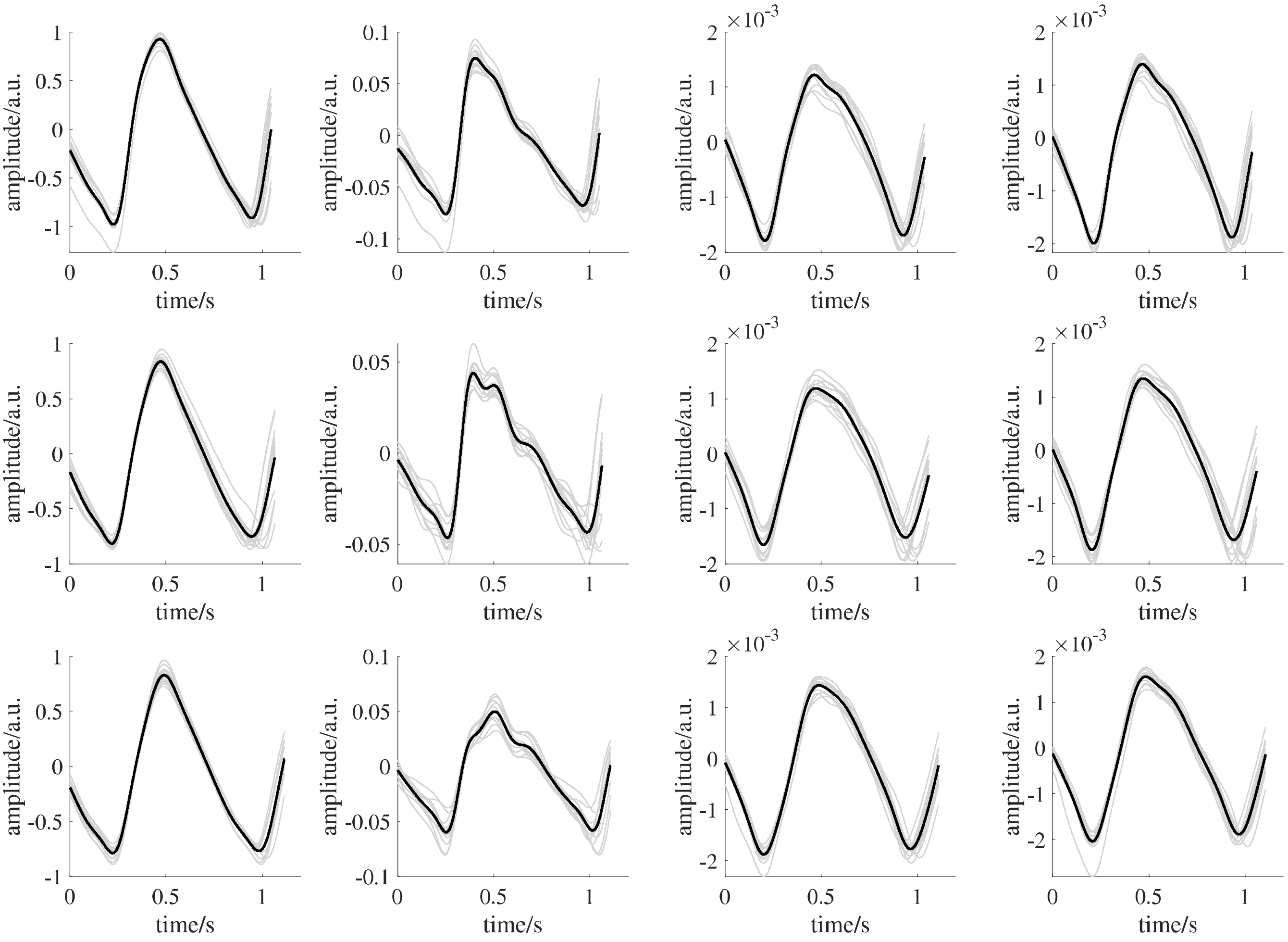

Figure S38. Template generation of one subject. From left to right: earlobe PPG (BL: 12 beats, ST1: 13 beats, ST2: 11 beats), finger PPG (BL: 12 beats, ST1: 13 beats, ST2: 11 beats), forehead iPPG (BL: 12 beats, ST1: 12 beats, ST2: 12 beats), super ROI iPPG (BL: 12 beats, ST1: 12 beats, ST2: 12 beats). The upper row shows signals during BL. The middle row shows signals during ST1. The lower row shows signals during ST2. Black lines indicate mean beat templates; gray lines indicate the corresponding beat segments.

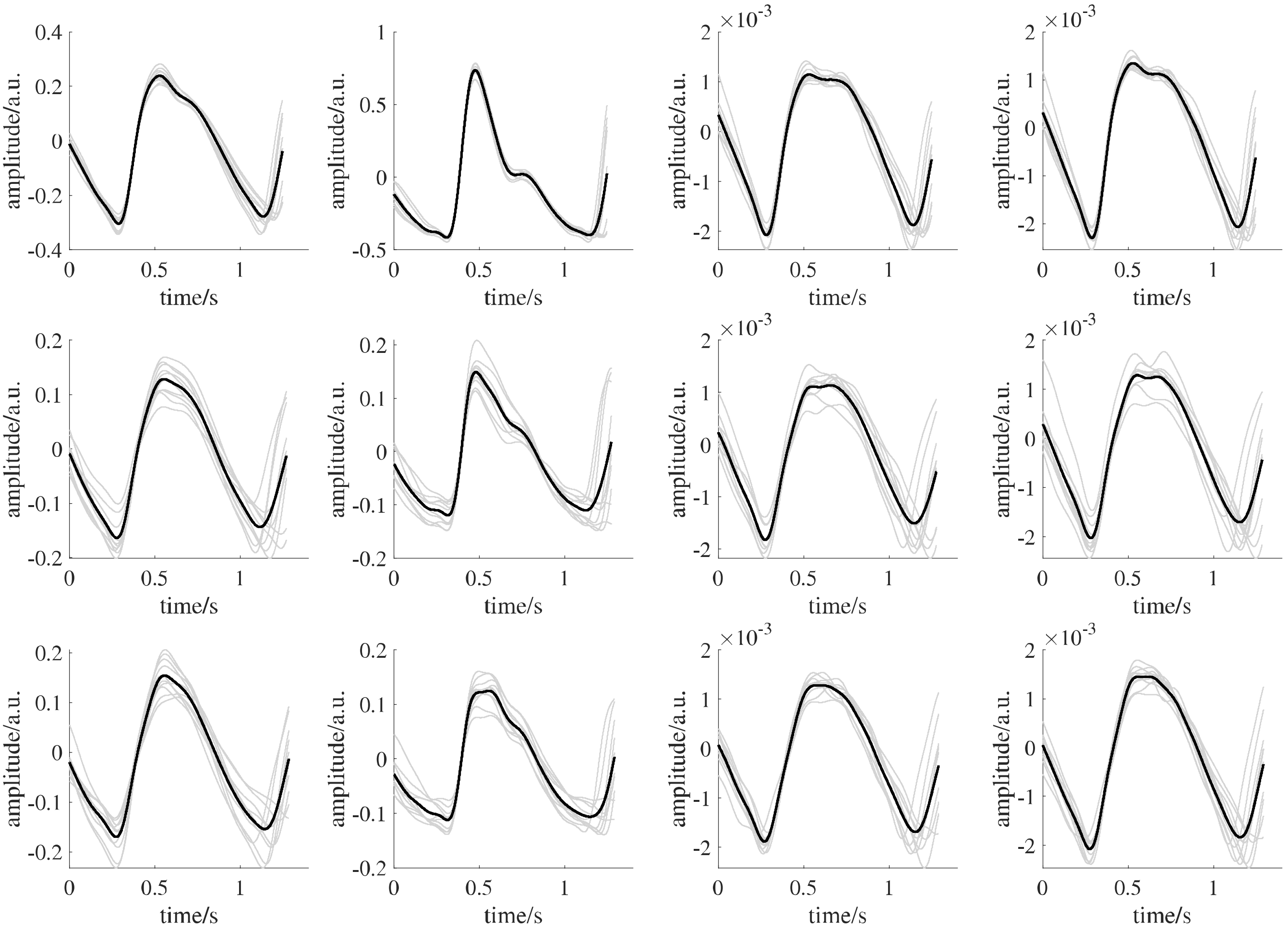

Figure S39. Template generation of one subject. From left to right: earlobe PPG (BL: 10 beats, ST1: 10 beats, ST2: 10 beats), finger PPG (BL: 10 beats, ST1: 10 beats, ST2: 10 beats), forehead iPPG (BL: 10 beats, ST1: 10 beats, ST2: 10 beats), super ROI iPPG (BL: 10 beats, ST1: 10 beats, ST2: 10 beats). The upper row shows signals during BL. The middle row shows signals during ST1. The lower row shows signals during ST2. Black lines indicate mean beat templates; gray lines indicate the corresponding beat segments.
